# Factor structure and reliability of the Family Resilience Scale (FRAS): adaptation with Colombian families exposed to stressful events

**DOI:** 10.3389/fpsyg.2025.1568139

**Published:** 2025-09-24

**Authors:** Paula Andrea Valencia Londoño, Sandra Patricia Trujillo Orrego, Luisa Fernanda Duque Monsalve, Luz Stella Giraldo Cardona

**Affiliations:** ^1^Facultad de Ciencias Sociales, Universidad de Medellín, Medellín, Colombia; ^2^Facultad de Jurisprudencia, Universidad del Rosario, Bogotá, Colombia; ^3^Facultad de Psicología, Universidad de San Buenaventura Medellín, Medellín, Colombia; ^4^Facultad de Ciencias de la Salud, Fundación Universitaria San Martín, Medellín, Colombia

**Keywords:** resilience, family, adaptation, coping, stress, communication, problem solving

## Abstract

**Antecedents:**

Resilience is the ability to face adversity and transform adversity positively. This concept has been transposed from the individual to the family context. Elements like assertive communication, cooperation, optimism, and support networks can dynamize the interaction and communication between family members and strengthen a family’s resilience and ability to overcome problems in contexts of social or environmental risk.

**Objective:**

To evaluate the psychometric properties, specifically the factor structure and internal consistency, of a Spanish-language adapted version of the Family Resilience Assessment Scale (FRAS) for use with Colombian families exposed to stressful events.

**Method:**

A Spanish-language adaptation of the Family Resilience Assessment Scale (FRAS) was applied to a sample of 303 Colombian families living in the municipalities of Salgar (*n* = 120) and Barbosa (n = 183), Antioquia and affected by different stressful events (risk of natural disaster or armed conflict). Of the total sample, 227 were women (75.9%) and 76 were men (25.1%). The highest percentage, 129 people, were in the age range of 26 to 45 years (42.6%) followed by 86 people (27.4%) who were in the age range of 46 to 60 years. Most respondents (120 people - 39.6%) gave no information about their schooling, followed by 17.5% (53 people) who reported to have Secondary Complete.

**Results:**

The confirmatory factor analysis revealed an acceptable but borderline fit of the original six-factor model (e.g., CFI = 0.938, RMSEA = 0.084). Internal consistency was adequate for all dimensions (*Ω* ≥ 0.7). These findings suggest that while the theoretical structure holds up reasonably well in the Colombian sample, some dimensions may be related to the context that do not optimally capture all of its nuances. Invariance analysis supported the equivalence of the model across gender at all levels (configural, metric, scalar, and strict), suggesting that the scale performs consistently for both men and women.

**Conclusion:**

The results suggest that the adaptation of the FRAS is reliable for the evaluation of family resilience in the Colombian population.

## 1 Introduction

In the last two decades, families worldwide have faced increasingly adverse conditions due to climate change and social issues such as unemployment, violence, migration, and limited access to education and healthcare services (Spring, 2016). Despite these challenges, many people have managed to confront and overcome adversity by leveraging personal and family resources, demonstrating a capacity known as resilience, defined from various perspectives as the ability to overcome adverse situations (Martin-Soelch and Schnyder, 2019).

Research on resilience began in the 1970s when Garmezy observed that some children exposed to extreme stress did not develop psychological problems but rather have a positive capacity for adaptation, marking a milestone in resilience studies (Garmezy, 1973), those capacities also reported in 1979 by Rutter, conceptualizing resilience as a person’s ability to confront, overcome, and positively transform adversity.

The concept of resilience has been applied to family and community contexts, adopting elements from systems theory that recognize internal dynamics such as assertive communication, cooperation, and optimism, which strengthen interaction among family members (Barcelata, 2018). Froma Walsh has contributed significantly, defining *family resilience* as adapting to adversity while maintaining family unity and improving the social environment (Walsh, 2016; Masten, 2018). Studies by Maurović et al. (2020) align with this perspective, identifying family resilience as a protective factor that reduces anxiety and depression, strengthens family bonds, and enhances interaction within the community. Cheng et al. (2024) emphasize that resilience is a dynamic process influenced by both individual and collective family systems. Moreover, Yang et al. (2023) argued that family resilience is not simply the sum of individual members’ resilience but a distinct construct shaped by shared beliefs, organizational patterns, and communal resources. Finally, Shao et al. (2023) highlighted those targeted programs focusing on communication, problem-solving, and emotional regulation can significantly strengthen family resilience.

Numerous studies have linked family functioning to the development of skills for decision-making and problem-solving in daily life. While some family resilience assessment methods use pathology-focused reports, there needs to be a consensus on the best instrument for measuring this capacity comprehensively and systematically (Prime et al., 2023). The lack of consensus on the definition and assessment of family resilience creates limitations in identifying key elements that foster resilience in the family setting (Hamilton and Carr, 2016).

Updating and improving assessment tools to better reflect current conditions and recognize the factors that foster resilience in the family context is crucial, addressing the elements that generate stress and critical events that affect family groups (Windle, 2011; Hamilton and Carr, 2016).

In Colombia, family resilience has been assessed using the Family Index of Regenerativity and Adaptation – General (FIRA-G; Rojas et al., 2021) and the Family Crisis Oriented Personal Evaluation Scales (F-COPES; Martín et al., 2007; Jiménez et al., 2012; Quintero et al., 2020), both grounded in the Family Adjustment and Adaptation Response (FAAR) model proposed by McCubbin et al. (1991, 2001). In other Latin American countries, additional instruments have been employed, such as the Family Resilience Scale (ERF) by Martínez et al. (2022), validated in Puerto Rico and also based on the FAAR model, and the Family Strengths Scale, originally developed by Olson and Larsen (1982) from a systemic perspective of family functioning, later adapted to the Spanish context by Sanz et al. (2002) and validated in Chile by Valenzuela and Rivadeneira (2021). To date, there is no known Spanish-language scale derived from Walsh (2016) Family Resilience Theory that assesses family resilience across diverse adversities. In the Latin American context, the only identified instrument based on this theoretical model is the Family Resilience Scale for Caregivers of People with Disabilities (ERF-PD), validated in Peru by Checcllo and Escudero (2023). However, this scale was developed to address a specific condition: disability.

These findings reveal a significant gap: there are no Spanish-language instruments, nor adaptations for the Latin American context, that assess family resilience based specifically on Walsh’s theoretical model. Most available scales are grounded in frameworks originally designed to examine family adaptation to crises over time—with either positive or negative outcomes—and only later incorporated concepts related to resilience (Patterson, 2002; McCubbin, 2013).

**Figure 1:**
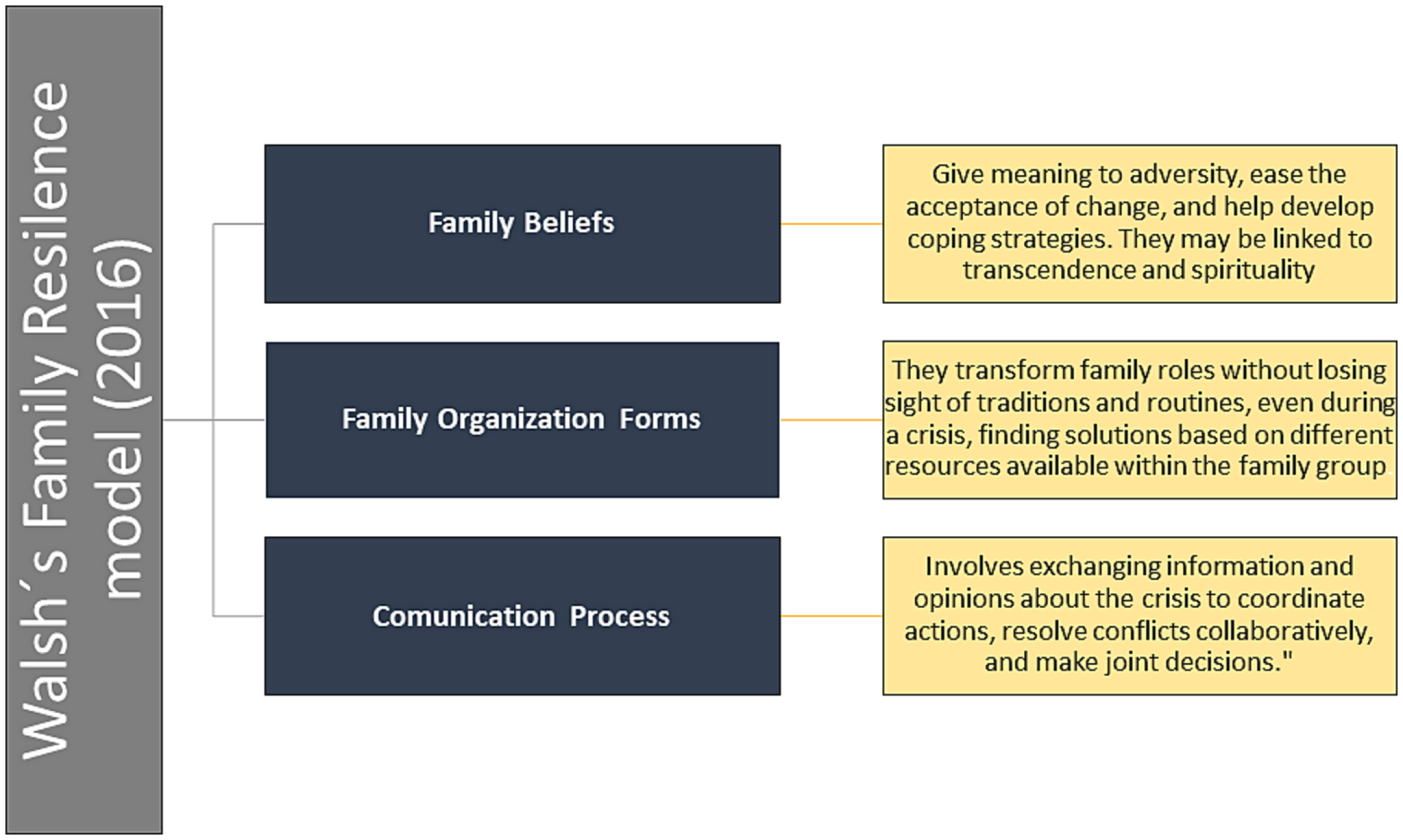
Dimensions of the family resilience model proposed by Walsh (2016).

Walsh (2002) Family Resilience Theory draws on an ecological perspective that acknowledges the recursive and synergistic interactions between individuals, families, and their broader social environments in shaping resilient responses. This theory adopts a strengths-based framework that views families as “challenged” rather than “damaged,” positing that even families experiencing dysfunction have the potential for growth and recovery (Walsh, 2003). Based on clinical work in family therapy and research conducted at the Chicago Center for Family Health (Walsh, 2016), the model outlines key family processes that promote resilience. These are organized into three major domains: (1) family belief systems (meaning-making, positive outlook, transcendence and spirituality); (2) organizational patterns (flexibility, connectedness, utilization of social and economic resources); and (3) communication and problem-solving processes (clear communication, emotional expression, collaborative problem-solving). Each domain comprises three specific sub-processes, yielding nine core dimensions of family resilience (see Figure 1).

In Walsh’s (2016) model, resilience is conceptualized as distinct from mere coping or survival. It implies positive adaptation and healthy family functioning in the face of adversity. The model is grounded in a systemic, ecological, and developmental approach that regards the family as a functional unit, considering its interactions with the social environment and its evolution over time. One of its strengths lies in its practical utility for clinical and community-based interventions, as it was explicitly designed to inform effective programs for families in crisis (Walsh, 2016).

A key feature of Walsh’s model is its emphasis on resilience as a dynamic, evolving process rather than a fixed trait or end state. Families may demonstrate resilience in some areas while remaining vulnerable in others, or fluctuate in their resilient responses over time. By focusing on dynamic processes—such as reconstructing family narratives or renegotiating routines—the model enables targeted interventions at any stage of a crisis (Walsh, 2016).

Given its theoretical robustness and clinical relevance, several scales have been developed based on Walsh’s model, including the Family Resilience Assessment Scale (FRAS; Sixbey, 2005), the Walsh Family Resilience Questionnaire (WFRQ; Walsh, 2016), the Family Resilience Assessment (FRA; Duncan Lane et al., 2016) for families affected by breast cancer, and the Family Resilience Questionnaire (FaRE; Faccio et al., 2019), designed for oncology settings.

For adaptation to the Colombian context, the FRAS (Sixbey, 2005) was selected due to several advantages. Its six-factor model offers greater differentiation among key resilience processes—for instance, distinguishing spirituality from a positive outlook—enabling more nuanced analysis in research and clinical settings. In contrast, the WFRQ evaluates only the three broad domains of the model. Compared to the WFRQ, FRA, and FaRE—which were designed for specific clinical populations—the FRAS offers a more generalizable approach. A noted limitation, however, is its length: 54 items compared to 32 in the WFRQ, 29 in the FRA, and 24 in the FaRE.

The decision to adapt the Family Resilience Assessment Scale (FRAS) instead of other family resilience scales was primarily based on its exceptional psychometric properties, which were highlighted in the systematic review conducted by Zhou et al. (2020). In their study, the authors evaluated the level of evidence for the quality of the measurement properties of 14 different family resilience scales applied across various global contexts. As a result, they recommended the FRAS as a particularly suitable tool for assessing family resilience to adversity in specific healthcare and social settings.

The FRAS has been extensively used internationally, facilitating comprehensive psychometric evaluations. A recent meta-analysis by Demir and Demircioğlu (2024), which synthesized data from 55 studies, reported strong generalized Cronbach’s alpha coefficients for the total score [*α* = 0.951; 95% CI (0.942, 0.958)] and for most subscales: 0.949 for Family Communication and Problem Solving, 0.792 for Utilizing Social and Economic Resources, 0.861 for Maintaining a Positive Outlook, 0.873 for Family Spirituality, 0.702 for Ability to Make Meaning of Adversity, and 0.635 for Family Connectedness—the latter showing comparatively lower reliability. The FRAS has been adapted into various languages across ten countries, as shown in Table 1.

**Table 1:** Summary of adaptation and validation studies of the family resilience assessment scale (FRAS).

Study (Year)	Country/Population	Analysis method	Resulting factor structure	Internal consistency (α)
Sixbey (2005)	United States/General population	Factor Analysis	Original 6-factor model with 54 items.	Total *α* = 0.96. Subscales between 0.70 and 0.96.
Dimech (2014)	Malta/General population	Factor Analysis	6-factor model, but with items grouped differently than the original.	Total *α* = 0.86. The “Family Connection” subscale had a very low *α* (0.22).
Kaya and Arici (2012)	Turkey/University students	CFA	4-factor model with 44 items. Excludes “Family connectedness” and “Family spirituality.”	Total *α* = 0.92.
Ferić et al. (2016).	Croatia/Parents of high school students	Factor Analysis	6-factor model, similar to the original.	4 of 6 factors with α between 0.65 and 0.92. Two factors had low consistency (*α* = 0.58 & 0.60).
Chew and Haase (2016).	Singapore/Adolescents with epilepsy	EFA	7-factor model explaining 83% of the variance.	Total *α* = 0.92.
Chiu et al. (2019)	Taiwan/Families of children with developmental delay	CFA	The original 6-factor model was supported.	Total *α* = 0.96. Subscales between 0.68 and 0.96.
Gardiner et al. (2019)	Canada/Families of children with ASD	CFA, EFA	The 6-factor model did not fit. EFA yielded a 3-factor model with 51 items.	High reliability in the three factors (*α* = 0.96, 0.88, and 0.92).
Nadrowska et al. (2021)	Poland/General population	CFA	The original 6-factor model fit adequately.	Subscales with α between 0.63 and 0.95.
Chu et al. (2022)	Hong Kong/Chinese family caregivers	EFA, CFA	5-factor model with 42 items.	Subscales with α between 0.724 and 0.963.
Harper and Debb (2022)	United States/African American college students	CFA	The 6-factor model did not fit and could not be respecified.	Not applicable, the model was inadequate.
de Oliveira et al. (2025)	Brazil/General population and individuals with chronic kidney disease	EFA, CFA	EFA suggested a four-factor model. A reduced 16-item, 4-factor model showed the best fit in CFA.	Subscales of the reduced model (CFA) with α between 0.78 and 0.88.

FRAS, family resilience assessment scale; EFA, exploratory factor analysis; CFA, confirmatory factor analysis; *α*, Cronbach’s Alpha; ASD, autism spectrum disorder. The table summarizes the psychometric properties reported in different adaptation and validation studies of the instrument.

In summary, the FRAS stands out as one of the most comprehensive and psychometrically validated instruments for assessing family resilience. Its broad international use, cross-cultural adaptations, and empirical support make it a highly valuable tool for research. Scholars recommend it for its robust capacity to detect variations across resilience dimensions and predict health outcomes (Zhou et al., 2020).

In clinical practice, the FRAS can guide professionals working with families facing adversity by identifying strengths and coping patterns and providing direction for targeted interventions. Responses can inform in-depth therapeutic interviews and complement other tools recommended by Walsh (2016), such as the genogram. Given the dynamic nature of family resilience, applying the FRAS at multiple points throughout the therapeutic process allows for tracking changes, offering families tangible feedback, reinforcing hope, and highlighting progress in their adaptive functioning.

Colombia’s myriad social and environmental issues offer a unique context for studying family resilience. Research on Colombian families affected by armed conflict, particularly forced displacement, reveals the adaptability of female-headed households^1^ in shared housing arrangements with extended family members (Utria Utria et al., 2015). Teenagers in these families often enter the workforce early to support their family income (Galindo Madero and De Oro, 2017). Each family member, assuming roles in problem-solving and protection, contributes to establishing new functional and emotional bonds. Studies highlight the essential role of family cohesion, clear communication, and mutual support in overcoming adversity (Domínguez De la Ossa, 2018).

Perceptions of stressors—such as armed violence or unemployment—can be reframed positively through future aspirations, parental resilience, humor, spirituality, and shared beliefs. Relating to urban areas with greater educational and work opportunities for displaced families can lessen stress and foster hope (Romero-Cardenas and Evies-Ojeda, 2018). Communication skills like listening and dialogue have also been linked to higher family cohesion, facilitating democratic decision-making and conflict resolution through horizontal relationships (Galindo Gálvez, 2017). In forced displacement contexts, mothers often act as “privileged narrators,” retelling family history to reframe adversity and foster solidarity and humor. Humor, associated with creativity and resilience, has been linked to higher resilience scores in children. In that sense, Families aware of their strengths, weaknesses, and collective identity are better equipped to develop internal resources and regulate emotions. For displaced families, self-awareness aids in overcoming stigmas, earning positive recognition in host communities, and promoting growth (Domínguez De la Ossa, 2018).

All this together highlights the need to adapt tools that assess family resilience in vulnerable contexts that have experienced various types of impact. Despite these challenges, these families have developed strategies alongside their family groups to overcome adversity.

The purpose of this study is to adapt and evaluate the psychometric properties of the Family Resilience Assessment Scale (FRAS) in the Colombian context. This task is particularly relevant given the scarcity of culturally appropriate instruments in the region, a gap also evident in other countries in the region, such as Brazil (de Oliveira et al., 2025). This work is positioned as a foundational first stage of cross-cultural adaptation, following the guidelines of the International Test Commission (2017) and Muñiz et al. (2013). Specifically, the objectives are to (1) determine the fit of the original six-dimension factor model using a Confirmatory Factor Analysis, and (2) estimate the internal consistency of each subscale. Based on prior evidence in other cultural contexts (e.g., Gardiner et al., 2019; Nadrowska et al., 2021), it is hypothesized that the six-factor structure will show an acceptable fit and that all subscales will demonstrate satisfactory internal consistency (*Ω* ≥ 0.70).

The implications of this study are twofold. First, it addresses a critical need by providing Spanish-speaking researchers and clinicians with a psychometrically sound tool, adapted for populations that have faced severe adversities such as armed conflict and natural disasters. This not only enables reliable assessment of resilience to inform the design of evidence-based clinical interventions and public policies aimed at strengthening families but also advances the scientific understanding of the construct within the specific sociocultural context of Colombia and Latin America, thereby contributing to a field of study that has been underdeveloped in the region.

## 2 Materials and methods

### 2.1 Sample

A sample of 303 individuals representing 303 households participated in this study. They were all residents of the settlement “La Primavera,” in the municipality of Barbosa (*n* = 183) or the sectors “La Aldea,” “La Habana,” and “La Florida” in the municipality of Salgar (*n* = 120) in the Department of Antioquia. To participate in the study, respondents had to be residents of one of the aforementioned settlements, have reached the age of majority (up to 18 years), and their family must have faced an adverse situation of large scale (a natural disaster, sociopolitical violence, forces displacement or forced migration, among others). The exclusion criteria considered the age of minority and the existence of a neurodegenerative disease that prevented the understanding of the statement of the document. A non-probabilistic convenience sampling method was used. Participants were recruited based on accessibility and their residence in communities exposed to natural disasters or armed conflict. The final sample included individuals who voluntarily agreed to participate after being contacted in their homes by trained field surveyors. Table 2, in the Results section below, details the sociodemographic information regarding age, gender, and highest level of education of the participants in the sample.

**Table 2:** Presentation of the sociodemographic information of the persons evaluated with the FRAS scale.

Variable	*N*	%
Gender	303	
Female	227	75.9
Male	76	25.1
Age range		
Under_age	1	0.3
18_25	25	8.3
26_45	129	42.6
46_60	83	27.4
Over 60	65	21.5
Schooling*		
N/A	1	0.3
None	16	5.3
No write and read	2	0.7
Not available	120	39.6
Elementary incomplete	41	13.5
Elementary complete	23	7.6
Secondary incomplete	37	12.2
Secondary complete	53	17.5
Technician	6	2.0
Technical	3	1.0
Professional	1	0.3
Sector		
La_Primavera	183	60.4
Salgar	120	39.6

*Schooling corresponds to the level of education reported by the participants; this classification is aligned with the parameters of the Colombian national education ministry.

### 2.2 Instrument

Family Resilience Assessment Scale (FRAS).

The Family Resilience Assessment Scale (FRAS) is a scale that measures family resilience based on different family processes. The scale was proposed by Sixbey in 2005 and has 54 items that correspond to six dimensions: Family communication and problem solving, use of social and economic resources, maintaining a positive attitude, family connection, family spirituality, and the capacity to make sense of adversity (i.e., “we accept stressful situation as a part of life”).

Each item on the survey is a four-point Likert scale question, with responses ranging from “totally disagree” (1) to “totally agree” (4). The points 1 to 4 correspond to the level of resilience, where 1 is the lowest and 4 is the highest. Items 33, 37, 45, and 50, however, are written in a negative form. To assign a score, the point values of the items corresponding to each of the six dimensions are added. Additionally, a total score is calculated by adding the values from each item. The final value ranges between 54 and 216. Higher scores overall indicate higher levels of family resilience.

The instrument has been translated and adapted to various languages and applied to different populations, demonstrating a good internal coherence in the total scores and subscales (*α* = 0.70–0.96) (Chew, and y Haase, A. M., 2016; Chiu et al., 2019; Dimech, 2014; Gardiner et al., 2019; Nadrowska et al., 2021).

### 2.3 Procedure

To adapt the scale, we followed the guidelines proposed by the International Test Commission (ITC) for Adaptation of the Test and the parameters proposed by Muñiz et al. (2013) and Pedrosa et al. (2013) for the construction and adaptation of the test. Aditionally, the adaptations of the scale to other languages and in other countries and the validations thereof were reviewed (See Table 1).

First, Dr. Meggen Tucker Sixbey, who holds the intellectual property rights for the test, was contacted via email and the pertinent permissions for the adaptation were obtained. Then, compliance with Colombian Law 1,090 of 2005, which establishes the Deontological and Bioethical Code and other provisions that regulate the practice of psychology, was verified. In Chapter VI, articles 45 to 48, Law 1,090 establishes the guidelines for the process of adaptation of psychological instruments, sets the necessary measurements for reliability and validity, and clarifies the reach and limitations of the psychological instruments that can be applied to individuals and communities.

Upon completing the review of the existing translations and adaptations of the FRAS from around the world (Table 1), the process of adapting the FRAS for the Colombian context began. Three different members of the research team made independent translations of the scale from English into Spanish: a doctor (PhD) of psychology who is fluent in English as a second language, a university instructor and researcher who holds a bachelor’s in foreign languages, and a psychologist and professional translator who was familiar with the theories of resilience. Afterwards, a focus group was conducted that included a group of experts who made a line-by-line comparison of the three translations to select the one that, in agreement with the criteria and consensus, best represented the meaning of the original language and was adapted to the linguistic and cultural context of the population. Then, an independent expert, a professional in social sciences whose native language is English, made a retranslation from Spanish to English. Comparing the retranslation with the original, the expert and the research group made the necessary adjustments to the Spanish text of some of the items. In this process, the expert in conjunction with the researchers compared the original version in English with the translation in Spanish, following the guidelines and quality control requirements for the translation and adaptation of the items (Hambleton and Zenisky, 2011).

Prior to the application of the questionnaire, a pilot study was conducted with a group of 21 people that met the criteria for inclusion in the sample (age of majority, members of families that had lived through traumatic events or family crisis). The feedback received from the participants of the pilot study was used to adjust a few items and better adapt them to the cultural and linguistic characteristics of the population. The final version of the scale can be found in Supplementary material 1. The application of the scale assessment was done in-situ by Pronósticos S. A. S Investigación Especializada, a professional survey service that provides surveyors for the application of the census and psychological evaluations. They took charge of the process of going door to door, applying for the survey, and entering the information gathered from the participants’ responses.

### 2.4 Data analysis

Internal consistency was evaluated using McDonald’s Omega (*Ω*), which is considered more appropriate than Cronbach’s Alpha in the context of ordinal data and confirmatory factor analysis (CFA) models. Unlike Alpha, Omega does not assume tau-equivalence (equal item loadings) and accounts for item-specific variance and error (McDonald, 1999; Flora, 2020). As the model was estimated using the WLSMV estimator—recommended for ordinal Likert-type responses—standard errors and confidence intervals for Omega could not be computed, due to limitations in variance estimation under this method.

The analysis of CFA followed conventional criteria based on changes in CFI, RMSEA, and chi-square difference tests. This step was included to examine whether the latent structure of the FRAS operates equivalently for male and female participants, especially in light of the sample’s gender imbalance. The diagonal weighted least squares method (WLSMV) was used as an estimator to extract the factors of the dichotomous qualitative variables. The WLSMV is appropriate in CFAs because it treats Likert scales as ordinal variables representing technical advantages. Furthermore, this estimator does not require a normal distribution of the data (Muthén and Muthén, 2002). Model fit indices and estimates of factor loadings were obtained for each item. Several indices were used to estimate the goodness of fit of the model: chi-square, comparative fit index (CFI), incremental fit index (IFI), Tucker-Lewis coefficient (TLI), and error of approximation (RMSEA). These goodness-of-fit indices allow us to evaluate the accuracy of the model data to determine if it is correct by evaluating the model’s overall fit. The chi-square is expected to be non-significant (although this indicator should not be used to rule out a model due to its sensitivity to sample size). TLI, IFI, and CFI should be closest to 1, although they are always expected to be higher than 0.90 (Bentler and Dudgeon, 1996; Hu and Bentler, 1995). The RSMEA value should be less than the critical value of 0.08 (Beribisky and Cribbie, 2024).

Given the ordinal nature of the Likert-scale data, we did not assume multivariate normality. Therefore, we used the WLSMV (Weighted Least Squares Mean and Variance adjusted) estimator, which is suitable for ordinal data and does not require multivariate normality assumptions (Muthén and Muthén, 2002). No model specifications were performed; our goal was to evaluate the original six-factor structure proposed by Sixbey (2005) as a theoretical model. While some items exhibited borderline or low factor loadings, they were retained to preserve the theoretical coherence of the model. These limitations are acknowledged and discussed in the Discussion section, with suggestions for future refinement. As a secondary analysis, an exploratory factor analysis (EFA) was conducted to examine the suitability of an alternative model for representing the study population. Due to practical constraints, including limited sample availability and other operational considerations, the estimation was performed using the same sample as the CFA. Given these limitations, the results are presented in Supplementary material.

To assess the measurement invariance of the model across gender, we conducted a multi-group confirmatory factor analysis following a sequential approach: configural, metric, scalar, and strict invariance.

A strategy was developed to meet the sample criteria needed for each type of validation in this study. The present study followed the parameter of 5 individuals per item for the validation of instruments (De Vet et al., 2011; Fritz et al., 2012). The final sample included in the model contained 303 subjects.

This study used R v. 4.41 software with packages such as lavaan and semTools to perform confirmatory factor analyses (CFA), allowing for specification and estimation of the factor model. These packages provided tools to calculate model fit indices and assess scale internal consistency using the Omega coefficient. The choice of R was based on its flexibility in modeling complex factor structures and the availability of CFA-specific functions, such as those offered by lavaan.

## 3 Results

### 3.1 Sociodemographic information of the sample

As shown in Table 2, 75.9% of the participants in the evaluation were women. Their ages ranged between 26 and 45. The information regarding educational attainment is limited because the data is not available for the participants from the municipality of Salgar.

### 3.2 Confirmatory factor analysis (CFA)

The confirmatory factor analysis (CFA) model of the FRAS scale in its adaptation shows an acceptable fit based on the CFI (0.938) and TLI (0.935) indices, suggesting that the original factorial structure^2^ has a reasonably solid foundation in the adapted population. Some items or factors may not fully align with the experiences and perspectives of the target population, warranting further careful evaluation. The RMSEA (0.084) and its confidence interval (0.081–0.087) evidence fit. This finding reflects that theoretical model and empirical data are near to the expecting values. Furthermore, the SRMR (0.095) reinforces the presence of notable differences between the observed and predicted correlations, indicating that some model dimensions may require modifications or adjustments to improve both statistical and conceptual coherence in this new context. Table 3 presents the estimations and factor structure of Sixbey six dimensions model.

**Table 3:** Factor structure of FRAS scale.

D1	Estimate	Std. error	*z*-value	*p*
FRAS_1	0.679	0.037	18.323	0.000
FRAS_6	0.881	0.015	57.596	0.000
FRAS_7	0.902	0.015	60.335	0.000
FRAS_8	0.765	0.026	29.222	0.000
FRAS_9	0.528	0.035	14.901	0.000
FRAS_10	0.247	0.042	5.887	0.000
FRAS_14	0.867	0.018	49.281	0.000
FRAS_15	0.866	0.012	72.559	0.000
FRAS_16	0.930	0.010	90.245	0.000
FRAS_17	0.883	0.018	50.264	0.000
FRAS_18	0.839	0.018	46.906	0.000
FRAS_20	0.908	0.011	83.626	0.000
FRAS_23	0.190	0.049	3.859	0.000
FRAS_24	0.791	0.028	28.403	0.000
FRAS_25	0.926	0.015	62.649	0.000
FRAS_26	0.884	0.014	63.927	0.000
FRAS_27	0.141	0.052	2.694	0.000
FRAS_28	0.874	0.019	47.196	0.000
FRAS_29	0.934	0.011	81.796	0.000
FRAS_30	0.713	0.023	30.562	0.000
FRAS_40	0.632	0.031	20.422	0.000
FRAS_41	0.782	0.024	33.072	0.000
FRAS_46	0.667	0.036	18.419	0.000
FRAS_48	0.829	0.019	43.253	0.000
FRAS_52	0.833	0.017	47.796	0.000
FRAS_53	0.752	0.032	23.498	0.000
FRAS_54	0.679	0.028	23.834	0.000

D2				
FRAS_11	0.694	0.028	24.365	0.000
FRAS_19	0.849	0.017	50.053	0.000
FRAS_31	0.809	0.020	41.058	0.000
FRAS_32	0.498	0.047	10.525	0.000
FRAS_38	0.229	0.039	5.803	0.000
FRAS_39	0.424	0.050	8.495	0.000
FRAS_43	0.038	0.057	0.659	0.510
FRAS_49	0.822	0.023	36.355	0.000

D3				
FRAS_13	0.827	0.015	54.572	0.000
FRAS_21	0.860	0.016	53.868	0.000
FRAS_22	0.880	0.015	58.229	0.000
FRAS_34	0.067	0.046	1.459	0.145
FRAS_36	0.615	0.034	18.294	0.000
FRAS_51	0.018	0.069	0.255	0.799

D4				
FRAS_2	0.571	0.037	15.464	0.000
FRAS_33	−0.465	0.049	−9.480	0.000
FRAS_37	−0.801	0.026	−30.654	0.000
FRAS_45	−0.206	0.053	−3.871	0.000
FRAS_47	0.824	0.018	46.908	0.000
	−0.127	0.056	−2.282	0.023

**D5**				
FRAS_12	0.910	0.013	69.252	0.000
FRAS_35	0.884	0.016	53.848	0.000
FRAS_42	0.770	0.032	23.843	0.000
FRAS_44	0.366	0.046	8.027	0.000

**D6**				
FRAS_3	0.865	0.018	47.985	0.000
FRAS_4	0.929	0.014	65.573	0.000
FRAS_	0.972	0.017	57.218	0.000

Description of Sixbey six dimensions model: **D1** = Family communication & problem-solving, **D2** = Utilizing social and economic resources **D3** = Maintaining a positive outlook, **D4** = Family connectedness, **D5** = Family spirituality, **D6** = Ability to make meaning of adversity.

Examination of the standardized factor loadings revealed that several items presented values below the recommended threshold of 0.40. In particular, items [e.g., Item 33, 37, 45, 50] showed weak or even negative loadings. While these items were retained in the analysis to maintain alignment with the original six-factor model, their performance suggests potential misalignment with how certain constructs are interpreted in the Colombian context. A detailed table of all factor loadings is provided in the Supplementary material.

The exploratory factor analysis (EFA) using oblimin rotation identified three factors explaining 59% of the cumulative variance. Although most items showed strong loadings (≥0.40) on their respective factors, the model was not the most parsimonious, with some cross-loadings and a negative loading for FRAS_37. Given that the EFA was conducted as an additional exploratory step rather than the primary objective of the study, these results are provided in the Supplementary material.

Measurement invariance analysis demonstrated that the model met the criteria for configural, metric, scalar, and strict invariance across sex. This suggests that both the factor structure and item intercepts are comparable between men and women, allowing meaningful group comparisons. Despite the gender imbalance in the sample (75.9% women), these findings support the replicability of the scale and allow for valid group comparisons. The fit indices for each step of the analysis are presented in Supplementary material 3. Scalar invariance showed no significant deterioration in model fit (*p* > 0.05), supporting the robustness of the model across gender groups.

### 3.3 Reliability of the scale

To assess evaluate the internal consistency of the model, the Cronbach Alpha coefficient and McDonald Omega coefficient were calculated (McDonald, 1999) for both the entire model and each of the three factors individually. Table 4 shows values of these indices for each dimension. To ensure the validity of the comparisons and that they reflect the construct being measured rather than being biased by the gender variable, a scalar invariance analysis by gender was conducted to ensure that the scores are unbiased. Scalar invariance showed that there is a significant decrease in fit when restricting the intercepts between groups (*p* > 0.05*p* > 0.05*p* > 0.05). This implies that the model is acceptable, and mean scores can be compared between men and women. For more information on invariance, see Supplementary material 3.

**Table 4:** Reliability of each dimensions of FRAS scale.

Dimension	Omega (Ω)
**D1** = Family communication and problem-solving (27)	0.96
**D2** = Utilizing social and economic resources (8)	0.8
**D3** = Maintaining a positive outlook (6)	0.81
**D4** = Family connectedness (6)	0.71
**D5** = Family spirituality (4)	0.73
**D6** = Ability to make meaning of adversity (3)	0.83

## 4 Discussion

The purpose of this study was to adapt and evaluate the psychometric properties of the Family Resilience Assessment Scale (FRAS) in the Colombian families expose to stressful context, specifically testing the hypothesis that its original six-factor structure would adequately fit the data. Our findings partially support this hypothesis. On one hand, the scale demonstrated solid internal consistency, with omega coefficients (*Ω*) exceeding the 0.7 threshold for each of the six dimensions, confirming its reliability in the sample. On the other hand, the confirmatory factor analysis (CFA) indicated that the original six-factor model has an acceptable but borderline fit to the data (CFI = 0.938, RMSEA = 0.084). This suggests that while the theoretical structure is reasonably robust, it may not optimally capture all the nuances of the construct in this particular context.

Based on Walsh’s recent studies, the theoretical approach to family resilience was revised (Walsh, 2016). Our review of the literature demonstrated that the theory of family resilience can be useful when evaluating families living in Colombia because the map of key processes of family resilience includes factors that coincide with the findings from Colombian-based studies. The adaptation is particularly relevant due to the scarcity of culturally validated instruments for assessing family resilience in Latin America. Specialized literature highlights the importance of using culturally appropriate scales, emphasizing that the translation of psychometric instruments must be carried out with methodological rigor to ensure their reliability and validity.

In our study, the confirmatory factor analysis (CFA) was performed using R software, which offers a robust and flexible platform for this type of analysis. The internal consistency of the scale was assessed using McDonald’s Omega coefficient (Ω), a more accurate reliability measure than Cronbach’s Alpha in the context of CFA, as it considers the specific variance of each item and in this case, as observed in the results of our study for each dimension. In that sense, the model of the FRAS scale in its adaptation shows an acceptable fit according to the CFI (0.938) and TLI (0.935) indices. These results suggest that the original factorial structure has a solid basis in the adapted population. However, the obtained values do not reach the optimal threshold (≥ 0.95), which might reflect cultural or conceptual differences in interpreting the dimensions of family resilience. This indicates that certain items or factors may not fully align with the experiences and perspectives of the target population, warranting further evaluation.

It is worth noting that other adaptations of the FRAS in different countries have also identified variations in its factorial structure. For instance, in Gardiner et al. (2019), the original FRAS model with 54 items and six dimensions did not completely fit in CFA. Instead, an exploratory factor analysis was conducted using the iterated principal factors method with Promax rotation. This analysis retained 51 items grouped into three factors: Family Communication and problem-solving, Use of Social and Economic Resources, and Family Spirituality, explaining 52% of the total variance. In contrast, studies conducted in Poland (Nadrowska et al., 2021) and China (Chu et al., 2022) successfully validated the original six-factor model and reported good psychometric properties.

Despite its limitations, the six-factor model identified in this study offers clear advantages over the three-factor solution obtained from the exploratory factor analysis (EFA) conducted as a secondary analysis. In the EFA (see Supplementary material), Dimension 1 grouped items primarily related to Family Communication and Problem-Solving, with a proportion of explained variance of 84%, suggesting a unidimensional structure of the scale. In contrast, Dimensions 2 and 3 combined items from different domains, revealing inconsistencies with Walsh’s theoretical model of Family Resilience. This tendency toward one-dimensionality is inconsistent with previous adaptations of the scale, which have typically yielded solutions of three (Gardiner et al., 2019), four (Kaya and Arici, 2012; Dong et al., 2018; Faqurudheen et al., 2014), five (Chu et al., 2022), six (Chiu et al., 2019; Isaacs et al., 2018; Nadrowska et al., 2021; Fan et al., 2017; Dimech, 2014; Ferić et al., 2016; Radetić-Paić and Černe, 2019), and seven (Chew, and y Haase, A. M., 2016; Yang et al., 2023) factors. By contrast, the confirmed six-factor solution, which more closely aligns with Walsh’s original proposal, enhances the scale’s utility for clinical and community-based interventions by enabling a more nuanced assessment of key resilience processes at different stages of intervention, thereby providing more precise guidance for treatment.

Due to levels of educational attainment and vulnerability, some of the items listed on the scale did not generate the expected results. For example, the participants reported that some of the items were reiterative, which led to an increase in affirmative responses. Chew, and y Haase, A. M. (2016) reported the same situation in their study.

The measurement invariance analysis across gender supports the robustness and generalizability of the adapted FRAS scale. All levels of invariance—configural, metric, scalar, and strict—were met, indicating that the scale measures the latent construct of family resilience equivalently in male and female respondents. This is particularly relevant given the gender imbalance in our sample (75.9% women), and it enhances the interpretability of the findings. Invariance testing has been emphasized in cross-cultural adaptations as a critical step to validate comparisons across groups (Byrne, 2012). The attainment of scalar and strict measurement invariance by gender represents a notable strength of the adapted model. These results provide evidence that the FRAS measures the same latent constructs in a consistent manner for both male and female respondents. This finding reinforces the structural validity of the scale and its potential applicability in gender-diverse populations, even in contexts with sociodemographic asymmetries.

In our work, the confirmation of the six-factor structure is a significant finding, as it aligns the Colombian version with adaptations from countries such as Poland and Taiwan, while also distinguishing it from others that have proposed more parsimonious alternative models of three, four, or five factors in contexts like Canada, Brazil, and Hong Kong. The borderline fit of the six-factor structure in our study can be attributed to the particularities of the population, which has faced chronic and high-intensity adversities like forced displacement and sociopolitical violence. It is possible that certain items or dimensions, such as “Family Connectedness” or “Spirituality,” acquire different meanings and manifestations in a context where community ties and shared beliefs have been reconfigured by trauma. Therefore, while the FRAS proves to be a robust and reliable instrument in its adapted form, the results also underscore the need to interpret its dimensions with cultural sensitivity and to recognize that its factor structure may be context-sensitive.

Although the CFI (0.938) and TLI (0.935) values suggest an acceptable model fit, the RMSEA (0.084) and SRMR (0.095) fall within a borderline range that warrants a cautious interpretation. According to Hu and Bentler (1995), values of RMSEA above 0.08 and SRMR above 0.09 may indicate moderate to poor fit. Several factors could account for these results. First, the FRAS includes 54 items, which increases model complexity and the likelihood of local misfit. Second, some items—especially those reverse-coded—may not have been interpreted consistently by all respondents, potentially reducing the coherence of item-factor relationships. Finally, the vulnerable nature of the sample (e.g., low education levels, exposure to displacement or violence) may have influenced how items were understood or responded to. Despite these issues, the model retains theoretical relevance and showed acceptable internal consistency across subscales, but future adaptations may benefit from refining or shortening the instrument.

In future studies, scholars might investigate convergent validations with other measures of family resilience adapted to the Colombian or Latin-American context. Also, future studies explore, through Exploratory Factor Analysis (EFA), the consistency of each dimension in vulnerable communities, as evaluated in this study. These elements also constitute an important part of the concept of family resilience for these populations. The above skills, attitudes, and practices allow families to understand adverse or stressful events as challenges to overcome, promoting growth and the development of individuals, families, and society. The study of family resilience makes visible the importance of promoting the capabilities and abilities of the family group, as the family is an agent that generates resilience. To generate resilience, cooperation, and positive, assertive communication of emotions between family members, oriented toward the development of capacities and strategies for effective coping for problem-solving, is important, as is the constant interaction of the family group with the support networks they find in their environment (Galindo Galvez, 2017).

The systematic review by Zhou et al. (2020) on the measurement properties of family resilience questionnaires recommends using the FRAS scale due to its optimal psychometric properties for assessing family resilience to adversity in health and social settings. Although this scale has been adapted to more than seven languages (see Table 1), it still needs a Spanish version. In the Latin American context, several scales of individual resilience have been created and validated (de la Paz Elez and Mercado García, 2018; Moscoso-Escalante and Castañeda-Chang, 2018; Sanjuan-Meza et al., 2018), and even as Navea Martín and Tamayo Hernández (2018) point out, the most widely used scale in studies of family resilience in health is the Connor-Davidson scale (CD-RISC), which focuses on individual aspects of resilience. Therefore, this study helps to address the lack of linguistically and culturally adapted scales for the Latin American context that measure collective family processes (such as communication, mutual support, and shared beliefs) rather than the resilience of family members as individuals. However, it should be noted that the sample of this study included only the Colombian population, so future research should analyze the scale’s psychometric properties in other Spanish-speaking countries and make the relevant linguistic adaptations, considering the cultural differences between countries.

## 5 Limitations and future perspectives

The study focused on Colombian families living in vulnerable conditions, but future work could explore the scale’s psychometric properties in other settings. Despite its contributions, this study has several limitations that should be acknowledged. First, the use of a non-probabilistic convenience sampling strategy limits the representativeness of the sample and, consequently, the generalizability of the findings to other populations. This limitation is especially relevant given the cultural and contextual specificity of the communities included in the study.

Although some subscales showed marginally acceptable reliability values (*Ω* ≈ 0.70), these results should be interpreted considering the known sensitivity of reliability estimates in scales with few items per factor and reverse-coded items. The choice of Ω over *α* is consistent with current psychometric recommendations for ordinal instruments (Flora, 2020).

It is important to note that items 33, 37, 45, and 50 were originally phrased in a negative direction and were reverse-coded before the analysis, following the FRAS scoring guidelines. Despite this, they unexpectedly showed negative factor loadings in the CFA. This finding suggests potential semantic or cultural inconsistencies in how these items were understood by participants, particularly in a vulnerable population with low educational attainment. Such behavior has been reported in previous FRAS adaptations (Chew and Haase 2016; Gardiner et al., 2019), and points to the possibility that the content of these items may not align well with the conceptual structure of their respective dimensions. Future studies should consider rephrasing or removing these items to ensure clearer measurement and reduce cognitive burden on respondents. In addition, it is recommended to include a control group to compare the levels of family resilience between vulnerable families and those not facing such conditions.

Another aspect to consider is the reliance on self-reports, which can introduce biases in the responses. To address this limitation, future studies could implement data triangulation methods, combining various information collection techniques, allowing for a more comprehensive family resilience assessment. Although we did not respecify the model, we recognize that certain items with weaker factor loadings may require further evaluation in future studies. Their retention in this analysis was based on a commitment to preserving the integrity of the original six-factor theoretical model. Although the CFI and TLI values fall within the acceptable range (≥ 0.90), the RMSEA value (0.084) exceeds the optimal threshold of 0.06 and falls into the “mediocre” range according to Hu and Bentler (1995). Similarly, the SRMR value of 0.095 is above the conventional cutoff of 0.08, indicating notable residuals. These findings, in line with recommendations by Beribisky and Cribbie (2024), highlight the need for a cautious interpretation of model fit, as acceptable global indices may coexist with localized item misfit. Therefore, we do not claim “good fit” conclusively but rather describe the model as showing an acceptable yet borderline structure that requires further evaluation in future adaptations. Although the psychometric evaluation was primarily focused on the scale’s factor structure and internal consistency, which are fundamental first steps, future research should provide additional evidence of validity. This includes examining convergent validity by comparing the FRAS with other relevant instruments, such as the F-COPES scale, which assesses family coping, and conducting discriminant validity analyses.

Despite the limitations, adapting the FRAS to the Colombian context represents a significant advance in assessing family resilience in vulnerable populations. This adapted version is expected to contribute to research and clinical practice, facilitating a deeper understanding of the factors promoting family resilience in adverse contexts. Therefore, future studies must use larger samples to perform different factorial analyses and provide evidence of convergent and discriminant validity (International Test Commission, 2017).

We hope that future scholars will replicate the application of the assessment scale with other similar at-risk populations (i.e., natural disasters, armed conflict) and carry out an analysis of the model that confirms the factor structure developed in this adaptation and contrasts its validity with other family resilience assessment scales (Bravo and López, 2015; Navea Martín and Tamayo Hernández, 2018). For the Colombian case, convergent validity analysis can be performed with the F-COPES (Family Crisis Oriented Personal Evaluation Scales), which does not assess family resilience but does assess family coping behavior in stressful situations and whose psychometric quality has been well established (Cabrera-García et al., 2023). To reduce cultural bias, the professional who performed the back-translation and the researchers of the team compared the English version (original) with the Spanish translation, taking into account the quality control questions of the translation-adaptation of the items of Hambleton and Zenisky, 2011, in future research, it is recommended that this process be carried out by independent experts who confirm the linguistic and cultural equivalence between the original scale and the scale adapted to Spanish. Furthermore, we hope that future research will identify variations in the analysis associated with the level of educational attainment in the population. We hope this adaptation’s results will lead to the creation of a short scale for the evaluation of family resilience in these populations.

Despite these limitations, this study represents a significant advance in the assessment of family resilience in vulnerable Colombian populations. We hope future research will build upon this work by using larger and more diverse samples and expanding the scope of psychometric validation in other communities in Colombia and Latin-America.

## 6 Conclusion

This study concludes that the Spanish-language adaptation of the Family Resilience Assessment Scale (FRAS) is a reliable and valid instrument for use in the Colombian context. The confirmatory factor analysis (CFA) indicates that the original six-factor structure proposed by Sixbey (2005) has an acceptable fit to the data from Colombian families exposed to adversity. While some fit indices were borderline (RMSEA = 0.084, SRMR = 0.095), others were adequate (CFI = 0.938, TLI = 0.935), supporting that the original model has a reasonably solid foundation in this new population. Furthermore, the scale demonstrated good internal consistency, with each of its dimensions achieving an Omega coefficient of 0.70 or higher. The primary contribution of this work is addressing the scarcity of culturally validated instruments for assessing collective family resilience in Latin America. In this region, most available scales focus on individual traits. This adapted version of the FRAS provides a crucial tool for researchers and clinicians in Colombia to understand better and support families facing significant stressors, such as armed conflict and natural disasters. Although the original six-factor structure is upheld, the findings also suggest that cultural nuances may warrant future exploration of the scale’s structure to optimize its application further.

## Data Availability

The original contributions presented in the study are included in the article/Supplementary material, further inquiries can be directed to the corresponding author/s.

## Footnotes

^1^According to the Administrative Department of the Public Function in Colombia, in its Radicado No. 20226000034961 of January 24, 2022, a woman head of household is “a Woman Head of Household, who being single or married, exercises the female head of household and has under her charge, affectively, economically or socially, permanently, her own minor children or other persons unable or incapacitated to work, either by permanent absence or physical, sensory, psychic or moral incapacity of the spouse or permanent partner or substantial deficiency of help from the other members of the family nucleus” (Paragraph. 7).

^2^The original factorial structure refers to the initial theoretical model of the FRAS developed by Sixbey (2005), which proposes six latent dimensions that explain family resilience from a systemic perspective.
