# Supplementary material for: Factor structure and reliability of the Family Resilience Scale (FRAS): adaptation with Colombian families exposed to stressful events
Source: Front Psychol. 2025 Sep 24;16:1568139. doi: 10.3389/fpsyg.2025.1568139 (PMC12506929; doi:10.3389/fpsyg.2025.1568139)
Supplement: Supplementary file 1 [file Supplementary_file_1.docx]

Supplementary Material 1

Final Version of the adapted Version of Family Resilience Scale (FRAS)

Lee atentamente cada una de las afirmaciones. Decide en qué medida describe a tu familia, desde tu punto de vista. Tu "familia" puede incluir a todas las personas que desees.

|  | **Completamente de acuerdo** | **De acuerdo** | **En desacuerdo** | **Completamente en desacuerdo** |
| --- | --- | --- | --- | --- |
| 1. Nuestra organización familiar es flexible para afrontar los imprevistos |  |  |  |  |
| 1. Nuestros amigos nos valoran por lo que somos   Alternativa: Nuestros amigos nos valoran tal y como somos |  |  |  |  |
| 1. Nos sentimos parte de la familia cuando nos ayudamos entre nosotros |  |  |  |  |
| 1. Aceptamos las situaciones estresantes como parte de la vida |  |  |  |  |
| 1. Todos aportamos a las decisiones importantes de la familia |  |  |  |  |
| 1. Somos capaces de superar el dolor y ponernos de acuerdo |  |  |  |  |
| 1. Nos adaptamos a las exigencias que se nos imponen como familia. |  |  |  |  |
| 1. En nuestra familia nos comprendemos mutuamente |  |  |  |  |
| 1. Asistimos a la iglesia |  |  |  |  |
| 1. Participamos en actividades de la iglesia. |  |  |  |  |
| 1. Podemos desahogarnos en casa sin que nadie se moleste |  |  |  |  |
| 1. Podemos llegar a un acuerdo cuando surgen problemas |  |  |  |  |
| 1. Podemos lidiar con las diferencias familiares a la hora de aceptar una pérdida |  |  |  |  |
| 1. Podemos aclarar lo que decimos para evitar malentendidos |  |  |  |  |
| 1. Podemos sobrevivir si surge otro problema. |  |  |  |  |
| 1. Podemos superar las dificultades como familia |  |  |  |  |
| 1. Consultamos entre nosotros las decisiones |  |  |  |  |
| 1. Asumimos los problemas de manera positiva para resolverlos |  |  |  |  |
| 1. Discutimos los problemas y nos sentimos bien con las soluciones |  |  |  |  |
| 1. Discutimos las cosas hasta tomar una decisión |  |  |  |  |
| 1. En nuestra familia aceptamos que hay nuevas formas de hacer las cosas |  |  |  |  |
| 1. Aceptamos que los problemas se presentan de forma inesperada |  |  |  |  |
| 1. Pedimos ayuda y colaboración a los vecinos |  |  |  |  |
| 1. Creemos que podemos manejar nuestros problemas |  |  |  |  |
| 1. Podemos pedir aclaraciones si no nos entendemos entre nosotros. |  |  |  |  |
| 1. Podemos ser honestos y directos entre nosotros |  |  |  |  |
| 1. Podemos contar con las personas en esta comunidad |  |  |  |  |
| 1. Podemos resolver problemas importantes |  |  |  |  |
| 1. Podemos hablar de la forma en que nos comunicamos en nuestra familia. |  |  |  |  |
| 1. Nos sentimos libres de expresar nuestras opiniones |  |  |  |  |
| 1. Nos sentimos bien dedicando tiempo y energía a nuestra familia |  |  |  |  |
| 1. Sentimos que las personas de la comunidad están dispuestas a ayudar en una emergencia. |  |  |  |  |
| 1. Nos sentimos seguros viviendo en esta comunidad |  |  |  |  |
| 1. Nos sentimos menospreciados por los miembros de la familia |  |  |  |  |
| 1. Nos sentimos fuertes al enfrentarnos a grandes problemas |  |  |  |  |
| 1. Tenemos fe en un ser supremo |  |  |  |  |
| 1. Tenemos la fortaleza suficiente para solucionar nuestros problemas |  |  |  |  |
| 1. Nos reservamos nuestros sentimientos |  |  |  |  |
| 1. Sabemos que la comunidad nos ayudará si tenemos problemas. |  |  |  |  |
| 1. Sabemos que somos importantes para nuestros amigos |  |  |  |  |
| 1. Aprendemos de los errores de los demás |  |  |  |  |
| 1. No damos rodeos cuando hablamos entre nosotros |  |  |  |  |
| 1. Recibimos regalos y favores de los vecinos |  |  |  |  |
| 1. Buscamos el consejo de asesores religiosos |  |  |  |  |
| 1. Rara vez escuchamos las preocupaciones o problemas de los miembros de la familia. |  |  |  |  |
| 1. Todos compartimos las responsabilidades en la familia |  |  |  |  |
| 1. Nos esforzamos por garantizar que los miembros de la familia no sufran daños emocionales o físicos. |  |  |  |  |
| 1. Demostramos amor y afecto por los miembros de la familia. |  |  |  |  |
| 1. Le decimos a nuestros familiares que nos importan |  |  |  |  |
| 1. Creemos que esta es una buena comunidad para criar a los niños |  |  |  |  |
| 1. Creemos que no deberíamos involucrarnos demasiado con la gente de esta comunidad. |  |  |  |  |
| 1. Confiamos en que todo va a salir bien a pesar de las dificultades |  |  |  |  |
| 1. Intentamos nuevas formas de solucionar los problemas |  |  |  |  |
| 1. Entendemos lo que otros miembros de la familia nos quieren decir |  |  |  |  |
