# Supplementary material for: Factor structure and reliability of the Family Resilience Scale (FRAS): adaptation with Colombian families exposed to stressful events
Source: Front Psychol. 2025 Sep 24;16:1568139. doi: 10.3389/fpsyg.2025.1568139 (PMC12506929; doi:10.3389/fpsyg.2025.1568139)
Supplement: Supplementary file 2 [file Supplementary_file_2.docx]

Supplementary Material 2

**Mean and Standard Deviation of the 54 Items FRAS Version**

|  | **Valid** | **Missing** | **Mean** | **Std. Deviation** | **Minimum** | **Maximum** |
| --- | --- | --- | --- | --- | --- | --- |
|  |  |  |  |  |  |  |
| **FRAS_1** | 284 | 0 | 2.053 | 0.469 | 1.000 | 3.000 |
|  |  |  |  |  |  |  |
| **FRAS_2** | 284 | 0 | 2.120 | 0.518 | 1.000 | 4.000 |
|  |  |  |  |  |  |  |
| **FRAS_3** | 284 | 0 | 1.937 | 0.371 | 1.000 | 3.000 |
|  |  |  |  |  |  |  |
| **FRAS_4** | 284 | 0 | 1.944 | 0.372 | 1.000 | 3.000 |
|  |  |  |  |  |  |  |
| **FRAS_5** | 284 | 0 | 1.968 | 0.406 | 1.000 | 3.000 |
|  |  |  |  |  |  |  |
| **FRAS_6** | 284 | 0 | 1.923 | 0.404 | 1.000 | 3.000 |
|  |  |  |  |  |  |  |
| **FRAS_7** | 284 | 0 | 1.961 | 0.414 | 1.000 | 3.000 |
|  |  |  |  |  |  |  |
| **FRAS_8** | 284 | 0 | 1.996 | 0.479 | 1.000 | 4.000 |
|  |  |  |  |  |  |  |
| **FRAS_9** | 284 | 0 | 2.102 | 0.576 | 1.000 | 4.000 |
|  |  |  |  |  |  |  |
| **FRAS_10** | 284 | 0 | 2.317 | 0.671 | 1.000 | 4.000 |
|  |  |  |  |  |  |  |
| **FRAS_11** | 284 | 0 | 1.958 | 0.427 | 1.000 | 4.000 |
|  |  |  |  |  |  |  |
| **FRAS_12** | 284 | 0 | 1.937 | 0.398 | 1.000 | 3.000 |
|  |  |  |  |  |  |  |
| **FRAS_13** | 284 | 0 | 1.923 | 0.396 | 1.000 | 3.000 |
|  |  |  |  |  |  |  |
| **FRAS_14** | 284 | 0 | 1.898 | 0.357 | 1.000 | 3.000 |
|  |  |  |  |  |  |  |
| **FRAS_15** | 284 | 0 | 1.951 | 0.426 | 1.000 | 3.000 |
|  |  |  |  |  |  |  |
| **FRAS_16** | 284 | 0 | 1.905 | 0.378 | 1.000 | 3.000 |
|  |  |  |  |  |  |  |
| **FRAS_17** | 284 | 0 | 1.951 | 0.409 | 1.000 | 4.000 |
|  |  |  |  |  |  |  |
| **FRAS_18** | 284 | 0 | 1.926 | 0.409 | 1.000 | 4.000 |
|  |  |  |  |  |  |  |
| **FRAS_19** | 284 | 0 | 1.944 | 0.408 | 1.000 | 3.000 |
|  |  |  |  |  |  |  |
| **FRAS_20** | 284 | 0 | 1.933 | 0.393 | 1.000 | 3.000 |
|  |  |  |  |  |  |  |
| **FRAS_21** | 284 | 0 | 1.951 | 0.400 | 1.000 | 3.000 |
|  |  |  |  |  |  |  |
| **FRAS_22** | 284 | 0 | 1.891 | 0.374 | 1.000 | 3.000 |
|  |  |  |  |  |  |  |
| **FRAS_23** | 284 | 0 | 2.331 | 0.567 | 1.000 | 4.000 |
|  |  |  |  |  |  |  |
| **FRAS_24** | 284 | 0 | 1.933 | 0.375 | 1.000 | 3.000 |
|  |  |  |  |  |  |  |
| **FRAS_25** | 284 | 0 | 1.926 | 0.364 | 1.000 | 3.000 |
|  |  |  |  |  |  |  |
| **FRAS_26** | 284 | 0 | 1.937 | 0.380 | 1.000 | 3.000 |
|  |  |  |  |  |  |  |
| **FRAS_27** | 284 | 0 | 2.141 | 0.477 | 1.000 | 4.000 |
|  |  |  |  |  |  |  |
| **FRAS_28** | 284 | 0 | 1.930 | 0.360 | 1.000 | 3.000 |
|  |  |  |  |  |  |  |
| **FRAS_29** | 284 | 0 | 1.940 | 0.376 | 1.000 | 3.000 |
|  |  |  |  |  |  |  |
| **FRAS_30** | 284 | 0 | 1.923 | 0.430 | 1.000 | 3.000 |
|  |  |  |  |  |  |  |
| **FRAS_31** | 284 | 0 | 1.930 | 0.388 | 1.000 | 3.000 |
|  |  |  |  |  |  |  |
| **FRAS_32** | 284 | 0 | 2.025 | 0.448 | 1.000 | 4.000 |
|  |  |  |  |  |  |  |
| **FRAS_33** | 284 | 0 | 2.915 | 0.504 | 1.000 | 4.000 |
|  |  |  |  |  |  |  |
| **FRAS_34** | 284 | 0 | 2.711 | 0.595 | 1.000 | 4.000 |
|  |  |  |  |  |  |  |
| **FRAS_35** | 284 | 0 | 1.979 | 0.436 | 1.000 | 3.000 |
|  |  |  |  |  |  |  |
| **FRAS_36** | 284 | 0 | 1.884 | 0.442 | 1.000 | 4.000 |
|  |  |  |  |  |  |  |
| **FRAS_37** | 284 | 0 | 3.102 | 0.385 | 2.000 | 4.000 |
|  |  |  |  |  |  |  |
| **FRAS_38** | 284 | 0 | 2.475 | 0.653 | 1.000 | 4.000 |
|  |  |  |  |  |  |  |
| **FRAS_39** | 284 | 0 | 2.088 | 0.534 | 1.000 | 4.000 |
|  |  |  |  |  |  |  |
| **FRAS_40** | 284 | 0 | 2.088 | 0.486 | 1.000 | 4.000 |
|  |  |  |  |  |  |  |
| **FRAS_41** | 284 | 0 | 1.979 | 0.411 | 1.000 | 4.000 |
|  |  |  |  |  |  |  |
| **FRAS_42** | 284 | 0 | 1.996 | 0.424 | 1.000 | 3.000 |
|  |  |  |  |  |  |  |
| **FRAS_43** | 284 | 0 | 2.384 | 0.604 | 1.000 | 4.000 |
|  |  |  |  |  |  |  |
| **FRAS_44** | 284 | 0 | 2.215 | 0.564 | 1.000 | 4.000 |
|  |  |  |  |  |  |  |
| **FRAS_45** | 284 | 0 | 2.736 | 0.592 | 1.000 | 4.000 |
|  |  |  |  |  |  |  |
| **FRAS_46** | 284 | 0 | 1.993 | 0.420 | 1.000 | 3.000 |
|  |  |  |  |  |  |  |
| **FRAS_47** | 284 | 0 | 1.965 | 0.435 | 1.000 | 3.000 |
|  |  |  |  |  |  |  |
| **FRAS_48** | 284 | 0 | 1.930 | 0.360 | 1.000 | 3.000 |
|  |  |  |  |  |  |  |
| **FRAS_49** | 284 | 0 | 1.944 | 0.372 | 1.000 | 3.000 |
|  |  |  |  |  |  |  |
| **FRAS_50** | 284 | 0 | 2.771 | 0.552 | 1.000 | 4.000 |
|  |  |  |  |  |  |  |
| **FRAS_51** | 284 | 0 | 2.218 | 0.533 | 1.000 | 3.000 |
|  |  |  |  |  |  |  |
| **FRAS_52** | 284 | 0 | 1.859 | 0.378 | 1.000 | 3.000 |
|  |  |  |  |  |  |  |
| **FRAS_53** | 284 | 0 | 1.923 | 0.377 | 1.000 | 4.000 |
|  |  |  |  |  |  |  |
| **FRAS_54** | 284 | 0 | 1.954 | 0.378 | 1.000 | 4.000 |
|  |  |  |  |  |  |  |
