# Supplementary material for: Factor structure and reliability of the Family Resilience Scale (FRAS): adaptation with Colombian families exposed to stressful events
Source: Front Psychol. 2025 Sep 24;16:1568139. doi: 10.3389/fpsyg.2025.1568139 (PMC12506929; doi:10.3389/fpsyg.2025.1568139)
Supplement: Supplementary file 3 [file Supplementary_file_3.docx]

Supplementary Material 3

Invariance model by gender Family Resilience Scale (FRAS)

| Chi-Squared Difference Test | |  |  |  |  |
| --- | --- | --- | --- | --- | --- |
|  |  |  |  |  |  |
| Df AIC BIC Chisq Chisq diff RMSEA Df diff Pr(>Chisq) | | | | | |
| modelo_configural 2724 10795 12087 7732.4 | | | | | |
| modelo_metrico 2772 10813 11930 7846.4 114.026 0.098422 48 2.697e-07 *** | | | | | |
| modelo_escalar 2820 10772 11714 7901.8 55.404 0.032958 48 0.2156 | | | | | |
| modelo_estricto 2874 10956 11701 8194.0 292.199 0.176250 54 < 2.2e-16 *** | | | | | |
| --- |  |  |  |  |  |
| Signif. codes: 0 ‘***’ 0.001 ‘**’ 0.01 ‘*’ 0.05 ‘.’ 0.1 ‘ ’ 1 | | | |  |  |
| lavaan 0.6-19 ended normally after 487 iterations |  |  |  |  |  |
|  |  |  |  |  |  |
| stimator ML | | | |  |  |
| Optimization method NLMINB | | | |  |  |
| Number of model parameters 354 | | | |  |  |
|  |  |  |  |  |  |
| Number of observations per group: | | | |  |  |
| 1 216 | | | |  |  |
| 2 68 | | | |  |  |
|  |  |  |  |  |  |
| Model Test User Model: | |  |  |  |  |
|  | | | |  |  |
| Test statistic 7732.408 | | | |  |  |
| Degrees of freedom 2724 | | | |  |  |
| P-value (Chi-square) 0.000 | | | |  |  |
| Test statistic for each group: | | |  |  |  |
| 1 3779.025 | | | |  |  |
| 2 3953.383 | | | |  |  |
|  |  |  |  |  |  |
| Model Test Baseline Model: | |  |  |  |  |
|  |  |  |  |  |  |
| Test statistic 15694.224 | | | |  |  |
| Degrees of freedom 2862 | | | |  |  |
| P-value 0.000 | | | |  |  |
|  |  |  |  |  |  |
| User Model versus Baseline Model: | | |  |  |  |
|  |  |  |  |  |  |
| Comparative Fit Index (CFI) 0.610 | | | |  |  |
| Tucker-Lewis Index (TLI) 0.590 | | | |  |  |
|  |  |  |  |  |  |
| Loglikelihood and Information Criteria: | | |  |  |  |
|  |  |  |  |  |  |
| Loglikelihood user model (H0) -5043.424 | | | |  |  |
| Loglikelihood unrestricted model (H1) -1177.220 | | | |  |  |
|  | | | |  |  |
| Akaike (AIC) 10794.848 | | | |  |  |
| Bayesian (BIC) 12086.585 | | | |  |  |
| Sample-size adjusted Bayesian (SABIC) 10964.038 | | | |  |  |
|  |  |  |  |  |  |
| Root Mean Square Error of Approximation: | | |  |  |  |
|  |  |  |  |  |  |
| RMSEA 0.114 | | | |  |  |
| 90 Percent confidence interval - lower 0.111 | | | |  |  |
| 90 Percent confidence interval - upper 0.117 | | | |  |  |
| P-value H_0: RMSEA <= 0.050 0.000 | | | |  |  |
| P-value H_0: RMSEA >= 0.080 1.000 | | | |  |  |
|  |  |  |  |  |  |
| Standardized Root Mean Square Residual: | | |  |  |  |
|  |  |  |  |  |  |
| SRMR 0.082 | | | |  |  |
|  |  |  |  |  |  |
| Parameter Estimates: | |  |  |  |  |
|  |  |  |  |  |  |
| Standard errors Standard | | | |  |  |
| Information Expected | | | |  |  |
| Information saturated (h1) model Structured | | | |  |  |
|  |  |  |  |  |  |
|  |  |  |  |  |  |
| Group 1 [1]: |  |  |  |  |  |
|  |  |  |  |  |  |
| Latent Variables: | |  |  |  |  |
| Estimate Std.Err z-value P(>\|z\|) | | | |  |  |
| D1 =~ | | | |  |  |
| FRAS_1 1.000 | | | |  |  |
| FRAS_6 1.267 0.160 7.924 0.000 | | | |  |  |
| FRAS_7 1.259 0.158 7.972 0.000 | | | |  |  |
| FRAS_8 1.281 0.177 7.254 0.000 | | | |  |  |
| FRAS_9 0.912 0.177 5.164 0.000 | | | |  |  |
| FRAS_10 0.371 0.189 1.964 0.049 | | | |  |  |
| FRAS_14 1.158 0.143 8.117 0.000 | | | |  |  |
| FRAS_15 1.329 0.167 7.943 0.000 | | | |  |  |
| FRAS_16 1.276 0.152 8.393 0.000 | | | |  |  |
| FRAS_17 1.250 0.151 8.294 0.000 | | | |  |  |
| FRAS_18 1.248 0.157 7.941 0.000 | | | |  |  |
| FRAS_20 1.326 0.157 8.457 0.000 | | | |  |  |
| FRAS_23 0.070 0.154 0.451 0.652 | | | |  |  |
| FRAS_24 1.140 0.146 7.786 0.000 | | | |  |  |
| FRAS_25 1.181 0.145 8.164 0.000 | | | |  |  |
| FRAS_26 1.233 0.147 8.396 0.000 | | | |  |  |
| FRAS_27 -0.055 0.132 -0.416 0.678 | | | |  |  |
| FRAS_28 1.106 0.142 7.813 0.000 | | | |  |  |
| FRAS_29 1.275 0.155 8.248 0.000 | | | |  |  |
| FRAS_30 1.236 0.161 7.659 0.000 | | | |  |  |
| FRAS_40 1.019 0.159 6.423 0.000 | | | |  |  |
| FRAS_41 1.093 0.152 7.182 0.000 | | | |  |  |
| FRAS_46 1.067 0.152 7.015 0.000 | | | |  |  |
| FRAS_48 1.106 0.140 7.894 0.000 | | | |  |  |
| FRAS_52 1.030 0.135 7.647 0.000 | | | |  |  |
| FRAS_53 0.971 0.137 7.113 0.000 | | | |  |  |
| FRAS_54 1.032 0.132 7.840 0.000 | | | |  |  |
| D2 =~ | | | |  |  |
| FRAS_11 1.000 | | | |  |  |
| FRAS_19 1.068 0.103 10.387 0.000 | | | |  |  |
| FRAS_31 1.018 0.103 9.898 0.000 | | | |  |  |
| FRAS_32 0.570 0.107 5.352 0.000 | | | |  |  |
| FRAS_38 0.492 0.154 3.200 0.001 | | | |  |  |
| FRAS_39 0.529 0.126 4.188 0.000 | | | |  |  |
| FRAS_43 -0.211 0.143 -1.475 0.140 | | | |  |  |
| FRAS_49 0.918 0.096 9.545 0.000 | | | |  |  |
| D3 =~ | | | |  |  |
| FRAS_13 1.000 | | | |  |  |
| FRAS_21 1.007 0.084 11.943 0.000 | | | |  |  |
| FRAS_22 0.951 0.076 12.582 0.000 | | | |  |  |
| FRAS_34 0.073 0.128 0.570 0.569 | | | |  |  |
| FRAS_36 1.013 0.091 11.160 0.000 | | | |  |  |
| FRAS_51 0.116 0.113 1.026 0.305 | | | |  |  |
| D4 =~ | | | |  |  |
| FRAS_2 1.000 | | | |  |  |
| FRAS_33 -0.638 0.169 -3.778 0.000 | | | |  |  |
| FRAS_37 -1.383 0.208 -6.644 0.000 | | | |  |  |
| FRAS_45 -0.351 0.185 -1.896 0.058 | | | |  |  |
| FRAS_47 1.419 0.220 6.458 0.000 | | | |  |  |
| FRAS_50 -0.258 0.170 -1.515 0.130 | | | |  |  |
| D5 =~ | | | |  |  |
| FRAS_12 1.000 | | | |  |  |
| FRAS_35 0.991 0.081 12.192 0.000 | | | |  |  |
| FRAS_42 0.826 0.083 9.934 0.000 | | | |  |  |
| FRAS_44 0.483 0.114 4.250 0.000 | | | |  |  |
| D6 =~ | | | |  |  |
| FRAS_3 1.000 | | | |  |  |
| FRAS_4 1.054 0.069 15.263 0.000 | | | |  |  |
| FRAS_5 1.062 0.076 13.891 0.000 | | | |  |  |
|  |  |  |  |  |  |
| Covariances: |  |  |  |  |  |
| Estimate Std.Err z-value P(>\|z\|) | | | |  |  |
| D1 ~~ | | | |  |  |
| D2 0.071 0.012 5.979 0.000 | | | |  |  |
| D3 0.078 0.012 6.357 0.000 | | | |  |  |
| D4 0.055 0.011 4.856 0.000 | | | |  |  |
| D5 0.083 0.013 6.446 0.000 | | | |  |  |
| D6 0.067 0.011 6.270 0.000 | | | |  |  |
| D2 ~~ | | | |  |  |
| D3 0.092 0.013 7.288 0.000 | | | |  |  |
| D4 0.065 0.012 5.211 0.000 | | | |  |  |
| D5 0.091 0.013 7.198 0.000 | | | |  |  |
| D6 0.080 0.011 7.148 0.000 | | | |  |  |
| D3 ~~ | | | |  |  |
| D4 0.074 0.013 5.529 0.000 | | | |  |  |
| D5 0.105 0.013 8.120 0.000 | | | |  |  |
| D6 0.079 0.011 7.466 0.000 | | | |  |  |
| D4 ~~ | | | |  |  |
| D5 0.072 0.013 5.463 0.000 | | | |  |  |
| D6 0.057 0.011 5.259 0.000 | | | |  |  |
| D5 ~~ | | | |  |  |
| D6 0.085 0.011 7.601 0.000 | | | |  |  |
|  |  |  |  |  |  |
| Intercepts: |  |  |  |  |  |
| Estimate Std.Err z-value P(>\|z\|) | | | |  |  |
| .FRAS_1 2.037 0.032 63.705 0.000 | | | |  |  |
| .FRAS_6 1.931 0.029 67.683 0.000 | | | |  |  |
| .FRAS_7 1.958 0.028 69.896 0.000 | | | |  |  |
| .FRAS_8 1.995 0.034 59.205 0.000 | | | |  |  |
| .FRAS_9 2.116 0.039 54.580 0.000 | | | |  |  |
| .FRAS_10 2.301 0.046 50.349 0.000 | | | |  |  |
| .FRAS_14 1.880 0.025 75.561 0.000 | | | |  |  |
| .FRAS_15 1.944 0.030 65.328 0.000 | | | |  |  |
| .FRAS_16 1.894 0.026 73.997 0.000 | | | |  |  |
| .FRAS_17 1.926 0.026 74.940 0.000 | | | |  |  |
| .FRAS_18 1.917 0.028 68.526 0.000 | | | |  |  |
| .FRAS_20 1.931 0.026 73.764 0.000 | | | |  |  |
| .FRAS_23 2.319 0.038 61.226 0.000 | | | |  |  |
| .FRAS_24 1.926 0.027 72.620 0.000 | | | |  |  |
| .FRAS_25 1.912 0.025 76.261 0.000 | | | |  |  |
| .FRAS_26 1.917 0.025 77.549 0.000 | | | |  |  |
| .FRAS_27 2.148 0.032 66.111 0.000 | | | |  |  |
| .FRAS_28 1.917 0.026 74.964 0.000 | | | |  |  |
| .FRAS_29 1.926 0.027 72.620 0.000 | | | |  |  |
| .FRAS_30 1.903 0.030 64.220 0.000 | | | |  |  |
| .FRAS_40 2.074 0.032 64.121 0.000 | | | |  |  |
| .FRAS_41 1.972 0.029 67.497 0.000 | | | |  |  |
| .FRAS_46 1.995 0.030 67.315 0.000 | | | |  |  |
| .FRAS_48 1.912 0.025 76.261 0.000 | | | |  |  |
| .FRAS_52 1.856 0.025 75.047 0.000 | | | |  |  |
| .FRAS_53 1.917 0.026 72.620 0.000 | | | |  |  |
| .FRAS_54 1.940 0.024 81.831 0.000 | | | |  |  |
| .FRAS_11 1.958 0.030 64.790 0.000 | | | |  |  |
| .FRAS_19 1.940 0.027 71.629 0.000 | | | |  |  |
| .FRAS_31 1.926 0.027 70.504 0.000 | | | |  |  |
| .FRAS_32 2.019 0.030 67.336 0.000 | | | |  |  |
| .FRAS_38 2.505 0.044 57.028 0.000 | | | |  |  |
| .FRAS_39 2.069 0.036 57.727 0.000 | | | |  |  |
| .FRAS_43 2.375 0.041 57.854 0.000 | | | |  |  |
| .FRAS_49 1.926 0.026 74.940 0.000 | | | |  |  |
| .FRAS_13 1.907 0.028 68.526 0.000 | | | |  |  |
| .FRAS_21 1.954 0.028 68.878 0.000 | | | |  |  |
| .FRAS_22 1.894 0.026 73.996 0.000 | | | |  |  |
| .FRAS_34 2.708 0.041 66.830 0.000 | | | |  |  |
| .FRAS_36 1.880 0.030 62.008 0.000 | | | |  |  |
| .FRAS_51 2.259 0.036 63.274 0.000 | | | |  |  |
| .FRAS_2 2.120 0.036 58.704 0.000 | | | |  |  |
| .FRAS_33 2.907 0.034 85.343 0.000 | | | |  |  |
| .FRAS_37 3.116 0.027 115.238 0.000 | | | |  |  |
| .FRAS_45 2.685 0.041 65.424 0.000 | | | |  |  |
| .FRAS_47 1.949 0.030 64.624 0.000 | | | |  |  |
| .FRAS_50 2.736 0.038 71.691 0.000 | | | |  |  |
| .FRAS_12 1.935 0.028 68.633 0.000 | | | |  |  |
| .FRAS_35 1.981 0.030 66.100 0.000 | | | |  |  |
| .FRAS_42 1.995 0.030 67.315 0.000 | | | |  |  |
| .FRAS_44 2.167 0.038 56.753 0.000 | | | |  |  |
| .FRAS_3 1.921 0.025 76.199 0.000 | | | |  |  |
| .FRAS_4 1.926 0.026 74.940 0.000 | | | |  |  |
| .FRAS_5 1.963 0.028 70.959 0.000 | | | |  |  |
|  |  |  |  |  |  |
| Variances: |  |  |  |  |  |
| Estimate Std.Err z-value P(>\|z\|) | | | |  |  |
| .FRAS_1 0.159 0.015 10.318 0.000 | | | |  |  |
| .FRAS_6 0.077 0.008 10.145 0.000 | | | |  |  |
| .FRAS_7 0.072 0.007 10.132 0.000 | | | |  |  |
| .FRAS_8 0.145 0.014 10.258 0.000 | | | |  |  |
| .FRAS_9 0.274 0.026 10.357 0.000 | | | |  |  |
| .FRAS_10 0.443 0.043 10.389 0.000 | | | |  |  |
| .FRAS_14 0.051 0.005 10.082 0.000 | | | |  |  |
| .FRAS_15 0.083 0.008 10.140 0.000 | | | |  |  |
| .FRAS_16 0.041 0.004 9.923 0.000 | | | |  |  |
| .FRAS_17 0.047 0.005 9.994 0.000 | | | |  |  |
| .FRAS_18 0.073 0.007 10.140 0.000 | | | |  |  |
| .FRAS_20 0.040 0.004 9.864 0.000 | | | |  |  |
| .FRAS_23 0.310 0.030 10.392 0.000 | | | |  |  |
| .FRAS_24 0.072 0.007 10.179 0.000 | | | |  |  |
| .FRAS_25 0.050 0.005 10.062 0.000 | | | |  |  |
| .FRAS_26 0.038 0.004 9.920 0.000 | | | |  |  |
| .FRAS_27 0.228 0.022 10.392 0.000 | | | |  |  |
| .FRAS_28 0.066 0.006 10.172 0.000 | | | |  |  |
| .FRAS_29 0.052 0.005 10.020 0.000 | | | |  |  |
| .FRAS_30 0.096 0.009 10.203 0.000 | | | |  |  |
| .FRAS_40 0.162 0.016 10.317 0.000 | | | |  |  |
| .FRAS_41 0.111 0.011 10.265 0.000 | | | |  |  |
| .FRAS_46 0.120 0.012 10.280 0.000 | | | |  |  |
| .FRAS_48 0.061 0.006 10.153 0.000 | | | |  |  |
| .FRAS_52 0.067 0.007 10.205 0.000 | | | |  |  |
| .FRAS_53 0.092 0.009 10.272 0.000 | | | |  |  |
| .FRAS_54 0.056 0.006 10.166 0.000 | | | |  |  |
| .FRAS_11 0.112 0.011 10.034 0.000 | | | |  |  |
| .FRAS_19 0.061 0.006 9.404 0.000 | | | |  |  |
| .FRAS_31 0.073 0.007 9.721 0.000 | | | |  |  |
| .FRAS_32 0.166 0.016 10.333 0.000 | | | |  |  |
| .FRAS_38 0.396 0.038 10.375 0.000 | | | |  |  |
| .FRAS_39 0.254 0.024 10.360 0.000 | | | |  |  |
| .FRAS_43 0.360 0.035 10.389 0.000 | | | |  |  |
| .FRAS_49 0.071 0.007 9.867 0.000 | | | |  |  |
| .FRAS_13 0.073 0.007 10.445 0.000 | | | |  |  |
| .FRAS_21 0.078 0.007 10.472 0.000 | | | |  |  |
| .FRAS_22 0.056 0.005 10.343 0.000 | | | |  |  |
| .FRAS_34 0.354 0.034 10.393 0.000 | | | |  |  |
| .FRAS_36 0.102 0.010 10.552 0.000 | | | |  |  |
| .FRAS_51 0.274 0.026 10.395 0.000 | | | |  |  |
| .FRAS_2 0.226 0.022 10.193 0.000 | | | |  |  |
| .FRAS_33 0.228 0.022 10.316 0.000 | | | |  |  |
| .FRAS_37 0.051 0.007 7.730 0.000 | | | |  |  |
| .FRAS_45 0.357 0.034 10.378 0.000 | | | |  |  |
| .FRAS_47 0.084 0.009 8.933 0.000 | | | |  |  |
| .FRAS_50 0.311 0.030 10.383 0.000 | | | |  |  |
| .FRAS_12 0.066 0.007 9.256 0.000 | | | |  |  |
| .FRAS_35 0.091 0.009 9.808 0.000 | | | |  |  |
| .FRAS_42 0.118 0.011 10.297 0.000 | | | |  |  |
| .FRAS_44 0.290 0.028 10.418 0.000 | | | |  |  |
| .FRAS_3 0.042 0.005 7.976 0.000 | | | |  |  |
| .FRAS_4 0.036 0.005 7.300 0.000 | | | |  |  |
| .FRAS_5 0.057 0.007 8.427 0.000 | | | |  |  |
| D1 0.061 0.015 4.145 0.000 | | | |  |  |
| D2 0.085 0.016 5.369 0.000 | | | |  |  |
| D3 0.094 0.014 6.509 0.000 | | | |  |  |
| D4 0.056 0.017 3.325 0.001 | | | |  |  |
| D5 0.105 0.016 6.765 0.000 | | | |  |  |
| D6 0.096 0.013 7.347 0.000 | | | |  |  |
|  |  |  |  |  |  |
|  |  |  |  |  |  |
| Group 2 [2]: |  |  |  |  |  |
|  |  |  |  |  |  |
| Latent Variables: | |  |  |  |  |
| Estimate Std.Err z-value P(>\|z\|) | | | |  |  |
| D1 =~ | | | |  |  |
| FRAS_1 1.000 | | | |  |  |
| FRAS_6 0.748 0.180 4.163 0.000 | | | |  |  |
| FRAS_7 1.111 0.228 4.865 0.000 | | | |  |  |
| FRAS_8 0.882 0.215 4.099 0.000 | | | |  |  |
| FRAS_9 1.040 0.293 3.555 0.000 | | | |  |  |
| FRAS_10 0.394 0.303 1.304 0.192 | | | |  |  |
| FRAS_14 1.037 0.187 5.556 0.000 | | | |  |  |
| FRAS_15 1.027 0.209 4.908 0.000 | | | |  |  |
| FRAS_16 1.246 0.223 5.582 0.000 | | | |  |  |
| FRAS_17 1.060 0.250 4.233 0.000 | | | |  |  |
| FRAS_18 1.256 0.231 5.430 0.000 | | | |  |  |
| FRAS_20 1.218 0.234 5.198 0.000 | | | |  |  |
| FRAS_23 0.632 0.277 2.281 0.023 | | | |  |  |
| FRAS_24 0.832 0.173 4.824 0.000 | | | |  |  |
| FRAS_25 1.009 0.193 5.223 0.000 | | | |  |  |
| FRAS_26 1.068 0.226 4.724 0.000 | | | |  |  |
| FRAS_27 0.636 0.225 2.829 0.005 | | | |  |  |
| FRAS_28 0.798 0.162 4.926 0.000 | | | |  |  |
| FRAS_29 1.015 0.186 5.457 0.000 | | | |  |  |
| FRAS_30 0.605 0.194 3.113 0.002 | | | |  |  |
| FRAS_40 0.751 0.247 3.042 0.002 | | | |  |  |
| FRAS_41 0.569 0.168 3.381 0.001 | | | |  |  |
| FRAS_46 0.602 0.178 3.376 0.001 | | | |  |  |
| FRAS_48 0.696 0.165 4.207 0.000 | | | |  |  |
| FRAS_52 1.126 0.229 4.929 0.000 | | | |  |  |
| FRAS_53 1.082 0.197 5.492 0.000 | | | |  |  |
| FRAS_54 0.577 0.215 2.677 0.007 | | | |  |  |
| D2 =~ | | | |  |  |
| FRAS_11 1.000 | | | |  |  |
| FRAS_19 1.638 0.324 5.051 0.000 | | | |  |  |
| FRAS_31 1.322 0.256 5.159 0.000 | | | |  |  |
| FRAS_32 0.857 0.284 3.015 0.003 | | | |  |  |
| FRAS_38 0.660 0.381 1.734 0.083 | | | |  |  |
| FRAS_39 0.739 0.322 2.297 0.022 | | | |  |  |
| FRAS_43 0.740 0.349 2.123 0.034 | | | |  |  |
| FRAS_49 0.984 0.231 4.260 0.000 | | | |  |  |
| D3 =~ | | | |  |  |
| FRAS_13 1.000 | | | |  |  |
| FRAS_21 1.516 0.229 6.615 0.000 | | | |  |  |
| FRAS_22 1.305 0.230 5.679 0.000 | | | |  |  |
| FRAS_34 0.016 0.331 0.048 0.962 | | | |  |  |
| FRAS_36 0.335 0.239 1.399 0.162 | | | |  |  |
| FRAS_51 -0.515 0.302 -1.703 0.089 | | | |  |  |
| D4 =~ | | | |  |  |
| FRAS_2 1.000 | | | |  |  |
| FRAS_33 -2.019 0.779 -2.593 0.010 | | | |  |  |
| FRAS_37 -0.955 0.412 -2.320 0.020 | | | |  |  |
| FRAS_45 -0.415 0.426 -0.975 0.330 | | | |  |  |
| FRAS_47 1.761 0.662 2.658 0.008 | | | |  |  |
| FRAS_50 -0.131 0.392 -0.333 0.739 | | | |  |  |
| D5 =~ | | | |  |  |
| FRAS_12 1.000 | | | |  |  |
| FRAS_35 1.013 0.135 7.517 0.000 | | | |  |  |
| FRAS_42 1.017 0.116 8.758 0.000 | | | |  |  |
| FRAS_44 0.325 0.215 1.514 0.130 | | | |  |  |
| D6 =~ | | | |  |  |
| FRAS_3 1.000 | | | |  |  |
| FRAS_4 1.141 0.206 5.531 0.000 | | | |  |  |
| FRAS_5 1.313 0.241 5.453 0.000 | | | |  |  |
|  |  |  |  |  |  |
| Covariances: |  |  |  |  |  |
| Estimate Std.Err z-value P(>\|z\|) | | | |  |  |
| D1 ~~ | | | |  |  |
| D2 0.057 0.017 3.428 0.001 | | | |  |  |
| D3 0.055 0.016 3.484 0.000 | | | |  |  |
| D4 0.040 0.017 2.360 0.018 | | | |  |  |
| D5 0.082 0.020 4.046 0.000 | | | |  |  |
| D6 0.058 0.017 3.471 0.001 | | | |  |  |
| D2 ~~ | | | |  |  |
| D3 0.039 0.012 3.419 0.001 | | | |  |  |
| D4 0.034 0.014 2.378 0.017 | | | |  |  |
| D5 0.063 0.016 4.032 0.000 | | | |  |  |
| D6 0.042 0.012 3.387 0.001 | | | |  |  |
| D3 ~~ | | | |  |  |
| D4 0.028 0.012 2.346 0.019 | | | |  |  |
| D5 0.059 0.014 4.095 0.000 | | | |  |  |
| D6 0.047 0.013 3.601 0.000 | | | |  |  |
| D4 ~~ | | | |  |  |
| D5 0.044 0.017 2.520 0.012 | | | |  |  |
| D6 0.034 0.014 2.370 0.018 | | | |  |  |
| D5 ~~ | | | |  |  |
| D6 0.058 0.015 3.893 0.000 | | | |  |  |
|  |  |  |  |  |  |
| Intercepts: |  |  |  |  |  |
| Estimate Std.Err z-value P(>\|z\|) | | | |  |  |
| .FRAS_1 2.103 0.056 37.843 0.000 | | | |  |  |
| .FRAS_6 1.897 0.042 44.832 0.000 | | | |  |  |
| .FRAS_7 1.971 0.051 38.778 0.000 | | | |  |  |
| .FRAS_8 2.000 0.051 39.260 0.000 | | | |  |  |
| .FRAS_9 2.059 0.072 28.719 0.000 | | | |  |  |
| .FRAS_10 2.368 0.080 29.489 0.000 | | | |  |  |
| .FRAS_14 1.956 0.039 50.751 0.000 | | | |  |  |
| .FRAS_15 1.971 0.046 42.500 0.000 | | | |  |  |
| .FRAS_16 1.941 0.046 42.242 0.000 | | | |  |  |
| .FRAS_17 2.029 0.059 34.564 0.000 | | | |  |  |
| .FRAS_18 1.956 0.048 40.344 0.000 | | | |  |  |
| .FRAS_20 1.941 0.050 38.484 0.000 | | | |  |  |
| .FRAS_23 2.368 0.072 32.996 0.000 | | | |  |  |
| .FRAS_24 1.956 0.039 50.751 0.000 | | | |  |  |
| .FRAS_25 1.971 0.041 47.551 0.000 | | | |  |  |
| .FRAS_26 2.000 0.051 39.260 0.000 | | | |  |  |
| .FRAS_27 2.118 0.057 37.107 0.000 | | | |  |  |
| .FRAS_28 1.971 0.036 54.975 0.000 | | | |  |  |
| .FRAS_29 1.985 0.039 51.079 0.000 | | | |  |  |
| .FRAS_30 1.985 0.049 40.731 0.000 | | | |  |  |
| .FRAS_40 2.132 0.062 34.359 0.000 | | | |  |  |
| .FRAS_41 2.000 0.042 48.083 0.000 | | | |  |  |
| .FRAS_46 1.985 0.044 45.037 0.000 | | | |  |  |
| .FRAS_48 1.985 0.039 51.079 0.000 | | | |  |  |
| .FRAS_52 1.868 0.051 36.957 0.000 | | | |  |  |
| .FRAS_53 1.941 0.041 47.371 0.000 | | | |  |  |
| .FRAS_54 2.000 0.055 36.347 0.000 | | | |  |  |
| .FRAS_11 1.956 0.044 44.663 0.000 | | | |  |  |
| .FRAS_19 1.956 0.053 37.077 0.000 | | | |  |  |
| .FRAS_31 1.941 0.041 47.371 0.000 | | | |  |  |
| .FRAS_32 2.044 0.057 36.049 0.000 | | | |  |  |
| .FRAS_38 2.382 0.081 29.548 0.000 | | | |  |  |
| .FRAS_39 2.147 0.067 32.223 0.000 | | | |  |  |
| .FRAS_43 2.412 0.073 33.152 0.000 | | | |  |  |
| .FRAS_49 2.000 0.042 48.083 0.000 | | | |  |  |
| .FRAS_13 1.971 0.041 47.551 0.000 | | | |  |  |
| .FRAS_21 1.941 0.041 47.371 0.000 | | | |  |  |
| .FRAS_22 1.882 0.044 42.528 0.000 | | | |  |  |
| .FRAS_34 2.721 0.072 38.008 0.000 | | | |  |  |
| .FRAS_36 1.897 0.052 36.813 0.000 | | | |  |  |
| .FRAS_51 2.088 0.065 32.181 0.000 | | | |  |  |
| .FRAS_2 2.118 0.057 37.108 0.000 | | | |  |  |
| .FRAS_33 2.941 0.062 47.452 0.000 | | | |  |  |
| .FRAS_37 3.059 0.041 74.644 0.000 | | | |  |  |
| .FRAS_45 2.897 0.063 46.077 0.000 | | | |  |  |
| .FRAS_47 2.015 0.049 41.335 0.000 | | | |  |  |
| .FRAS_50 2.882 0.061 47.455 0.000 | | | |  |  |
| .FRAS_12 1.941 0.041 47.371 0.000 | | | |  |  |
| .FRAS_35 1.971 0.051 38.778 0.000 | | | |  |  |
| .FRAS_42 2.000 0.047 43.007 0.000 | | | |  |  |
| .FRAS_44 2.368 0.065 36.174 0.000 | | | |  |  |
| .FRAS_3 1.985 0.044 45.037 0.000 | | | |  |  |
| .FRAS_4 2.000 0.042 48.083 0.000 | | | |  |  |
| .FRAS_5 1.985 0.049 40.731 0.000 | | | |  |  |
|  |  |  |  |  |  |
| Variances: |  |  |  |  |  |
| Estimate Std.Err z-value P(>\|z\|) | | | |  |  |
| .FRAS_1 0.137 0.024 5.806 0.000 | | | |  |  |
| .FRAS_6 0.081 0.014 5.807 0.000 | | | |  |  |
| .FRAS_7 0.086 0.015 5.781 0.000 | | | |  |  |
| .FRAS_8 0.120 0.021 5.809 0.000 | | | |  |  |
| .FRAS_9 0.271 0.047 5.817 0.000 | | | |  |  |
| .FRAS_10 0.427 0.073 5.830 0.000 | | | |  |  |
| .FRAS_14 0.023 0.004 5.649 0.000 | | | |  |  |
| .FRAS_15 0.070 0.012 5.778 0.000 | | | |  |  |
| .FRAS_16 0.031 0.005 5.633 0.000 | | | |  |  |
| .FRAS_17 0.153 0.026 5.806 0.000 | | | |  |  |
| .FRAS_18 0.045 0.008 5.701 0.000 | | | |  |  |
| .FRAS_20 0.065 0.011 5.749 0.000 | | | |  |  |
| .FRAS_23 0.321 0.055 5.827 0.000 | | | |  |  |
| .FRAS_24 0.051 0.009 5.783 0.000 | | | |  |  |
| .FRAS_25 0.043 0.007 5.745 0.000 | | | |  |  |
| .FRAS_26 0.094 0.016 5.789 0.000 | | | |  |  |
| .FRAS_27 0.192 0.033 5.824 0.000 | | | |  |  |
| .FRAS_28 0.041 0.007 5.776 0.000 | | | |  |  |
| .FRAS_29 0.028 0.005 5.692 0.000 | | | |  |  |
| .FRAS_30 0.135 0.023 5.822 0.000 | | | |  |  |
| .FRAS_40 0.221 0.038 5.822 0.000 | | | |  |  |
| .FRAS_41 0.094 0.016 5.819 0.000 | | | |  |  |
| .FRAS_46 0.106 0.018 5.819 0.000 | | | |  |  |
| .FRAS_48 0.068 0.012 5.806 0.000 | | | |  |  |
| .FRAS_52 0.082 0.014 5.776 0.000 | | | |  |  |
| .FRAS_53 0.029 0.005 5.679 0.000 | | | |  |  |
| .FRAS_54 0.182 0.031 5.825 0.000 | | | |  |  |
| .FRAS_11 0.091 0.015 6.106 0.000 | | | |  |  |
| .FRAS_19 0.083 0.014 6.114 0.000 | | | |  |  |
| .FRAS_31 0.045 0.007 6.051 0.000 | | | |  |  |
| .FRAS_32 0.190 0.032 5.959 0.000 | | | |  |  |
| .FRAS_38 0.425 0.072 5.868 0.000 | | | |  |  |
| .FRAS_39 0.280 0.048 5.900 0.000 | | | |  |  |
| .FRAS_43 0.338 0.057 5.889 0.000 | | | |  |  |
| .FRAS_49 0.079 0.013 6.121 0.000 | | | |  |  |
| .FRAS_13 0.069 0.012 5.705 0.000 | | | |  |  |
| .FRAS_21 0.004 0.004 0.908 0.364 | | | |  |  |
| .FRAS_22 0.051 0.009 5.477 0.000 | | | |  |  |
| .FRAS_34 0.348 0.060 5.831 0.000 | | | |  |  |
| .FRAS_36 0.175 0.030 5.826 0.000 | | | |  |  |
| .FRAS_51 0.274 0.047 5.824 0.000 | | | |  |  |
| .FRAS_2 0.201 0.034 5.882 0.000 | | | |  |  |
| .FRAS_33 0.176 0.031 5.608 0.000 | | | |  |  |
| .FRAS_37 0.095 0.016 5.871 0.000 | | | |  |  |
| .FRAS_45 0.265 0.045 5.842 0.000 | | | |  |  |
| .FRAS_47 0.097 0.018 5.338 0.000 | | | |  |  |
| .FRAS_50 0.251 0.043 5.832 0.000 | | | |  |  |
| .FRAS_12 0.026 0.005 4.943 0.000 | | | |  |  |
| .FRAS_35 0.085 0.015 5.796 0.000 | | | |  |  |
| .FRAS_42 0.056 0.010 5.643 0.000 | | | |  |  |
| .FRAS_44 0.282 0.048 5.836 0.000 | | | |  |  |
| .FRAS_3 0.078 0.014 5.489 0.000 | | | |  |  |
| .FRAS_4 0.047 0.010 4.877 0.000 | | | |  |  |
| .FRAS_5 0.067 0.014 4.987 0.000 | | | |  |  |
| D1 0.073 0.027 2.657 0.008 | | | |  |  |
| D2 0.040 0.015 2.575 0.010 | | | |  |  |
| D3 0.048 0.016 2.935 0.003 | | | |  |  |
| D4 0.021 0.015 1.350 0.177 | | | |  |  |
| D5 0.088 0.019 4.575 0.000 | | | |  |  |
| D6 0.055 0.019 2.880 0.004 | | | |  |  |
|  |  |  |  |  |  |
| lavaan 0.6-19 ended normally after 416 iterations | | | |  |  |
|  |  |  |  |  |  |
| Estimator ML | | | |  |  |
| Optimization method NLMINB | | | |  |  |
| Number of model parameters 354 | | | |  |  |
| Number of equality constraints 48 | | | |  |  |
|  |  |  |  |  |  |
| Number of observations per group: | | | |  |  |
| 1 216 | | | |  |  |
| 2 68 | | | |  |  |
|  |  |  |  |  |  |
| Model Test User Model: | |  |  |  |  |
|  | | | |  |  |
| Test statistic 7846.434 | | | |  |  |
| Degrees of freedom 2772 | | | |  |  |
| P-value (Chi-square) 0.000 | | | |  |  |
| Test statistic for each group: | | |  |  |  |
| 1 3801.970 | | | |  |  |
| 2 4044.464 | | | |  |  |
|  |  |  |  |  |  |
| Model Test Baseline Model: | |  |  |  |  |
|  |  |  |  |  |  |
| Test statistic 15694.224 | | | |  |  |
| Degrees of freedom 2862 | | | |  |  |
| P-value 0.000 | | | |  |  |
|  |  |  |  |  |  |
| User Model versus Baseline Model: | | |  |  |  |
|  |  |  |  |  |  |
| Comparative Fit Index (CFI) 0.605 | | | |  |  |
| Tucker-Lewis Index (TLI) 0.592 | | | |  |  |
|  |  |  |  |  |  |
| Loglikelihood and Information Criteria: | | |  |  |  |
|  |  |  |  |  |  |
| Loglikelihood user model (H0) -5100.437 | | | |  |  |
| Loglikelihood unrestricted model (H1) -1177.220 | | | |  |  |
|  | | | |  |  |
| Akaike (AIC) 10812.874 | | | |  |  |
| Bayesian (BIC) 11929.461 | | | |  |  |
| Sample-size adjusted Bayesian (SABIC) 10959.123 | | | |  |  |
|  |  |  |  |  |  |
| Root Mean Square Error of Approximation: | | |  |  |  |
|  |  |  |  |  |  |
| RMSEA 0.114 | | | |  |  |
| 90 Percent confidence interval - lower 0.111 | | | |  |  |
| 90 Percent confidence interval - upper 0.117 | | | |  |  |
| P-value H_0: RMSEA <= 0.050 0.000 | | | |  |  |
| P-value H_0: RMSEA >= 0.080 1.000 | | | |  |  |
|  |  |  |  |  |  |
| Standardized Root Mean Square Residual: | | |  |  |  |
|  |  |  |  |  |  |
| SRMR 0.098 | | | |  |  |
|  |  |  |  |  |  |
| Parameter Estimates: | |  |  |  |  |
|  |  |  |  |  |  |
| Standard errors Standard | | | |  |  |
| Information Expected | | | |  |  |
| Information saturated (h1) model Structured | | | |  |  |
|  |  |  |  |  |  |
|  |  |  |  |  |  |
| Group 1 [1]: |  |  |  |  |  |
|  |  |  |  |  |  |
| Latent Variables: | |  |  |  |  |
| Estimate Std.Err z-value P(>\|z\|) | | | |  |  |
| D1 =~ | | | |  |  |
| FRAS_1 1.000 | | | |  |  |
| FRAS_6 (.p2.) 1.148 0.125 9.146 0.000 | | | |  |  |
| FRAS_7 (.p3.) 1.222 0.130 9.365 0.000 | | | |  |  |
| FRAS_8 (.p4.) 1.177 0.140 8.389 0.000 | | | |  |  |
| FRAS_9 (.p5.) 0.941 0.151 6.217 0.000 | | | |  |  |
| FRAS_10 (.p6.) 0.382 0.160 2.381 0.017 | | | |  |  |
| FRAS_14 (.p7.) 1.133 0.116 9.790 0.000 | | | |  |  |
| FRAS_15 (.p8.) 1.248 0.134 9.329 0.000 | | | |  |  |
| FRAS_16 (.p9.) 1.270 0.127 10.005 0.000 | | | |  |  |
| FRAS_17 (.10.) 1.199 0.124 9.640 0.000 | | | |  |  |
| FRAS_18 (.11.) 1.263 0.132 9.564 0.000 | | | |  |  |
| FRAS_20 (.12.) 1.286 0.129 9.948 0.000 | | | |  |  |
| FRAS_23 (.13.) 0.193 0.134 1.442 0.149 | | | |  |  |
| FRAS_24 (.14.) 1.057 0.115 9.160 0.000 | | | |  |  |
| FRAS_25 (.15.) 1.137 0.118 9.661 0.000 | | | |  |  |
| FRAS_26 (.16.) 1.186 0.121 9.816 0.000 | | | |  |  |
| FRAS_27 (.17.) 0.120 0.113 1.064 0.287 | | | |  |  |
| FRAS_28 (.18.) 1.018 0.110 9.216 0.000 | | | |  |  |
| FRAS_29 (.19.) 1.202 0.122 9.841 0.000 | | | |  |  |
| FRAS_30 (.20.) 1.113 0.128 8.679 0.000 | | | |  |  |
| FRAS_40 (.21.) 0.961 0.132 7.258 0.000 | | | |  |  |
| FRAS_41 (.22.) 0.957 0.118 8.098 0.000 | | | |  |  |
| FRAS_46 (.23.) 0.952 0.120 7.946 0.000 | | | |  |  |
| FRAS_48 (.24.) 1.012 0.111 9.118 0.000 | | | |  |  |
| FRAS_52 (.25.) 1.037 0.115 9.025 0.000 | | | |  |  |
| FRAS_53 (.26.) 1.062 0.116 9.159 0.000 | | | |  |  |
| FRAS_54 (.27.) 0.965 0.108 8.915 0.000 | | | |  |  |
| D2 =~ | | | |  |  |
| FRAS_11 1.000 | | | |  |  |
| FRAS_19 (.29.) 1.170 0.102 11.477 0.000 | | | |  |  |
| FRAS_31 (.30.) 1.080 0.097 11.164 0.000 | | | |  |  |
| FRAS_32 (.31.) 0.606 0.101 5.985 0.000 | | | |  |  |
| FRAS_38 (.32.) 0.518 0.144 3.590 0.000 | | | |  |  |
| FRAS_39 (.33.) 0.563 0.119 4.729 0.000 | | | |  |  |
| FRAS_43 (.34.) -0.059 0.132 -0.443 0.658 | | | |  |  |
| FRAS_49 (.35.) 0.943 0.091 10.388 0.000 | | | |  |  |
| D3 =~ | | | |  |  |
| FRAS_13 1.000 | | | |  |  |
| FRAS_21 (.37.) 1.136 0.077 14.779 0.000 | | | |  |  |
| FRAS_22 (.38.) 1.000 0.072 13.907 0.000 | | | |  |  |
| FRAS_34 (.39.) 0.075 0.120 0.630 0.529 | | | |  |  |
| FRAS_36 (.40.) 0.969 0.086 11.301 0.000 | | | |  |  |
| FRAS_51 (.41.) 0.028 0.106 0.264 0.792 | | | |  |  |
| D4 =~ | | | |  |  |
| FRAS_2 1.000 | | | |  |  |
| FRAS_33 (.43.) -0.813 0.167 -4.863 0.000 | | | |  |  |
| FRAS_37 (.44.) -1.340 0.186 -7.194 0.000 | | | |  |  |
| FRAS_45 (.45.) -0.356 0.166 -2.145 0.032 | | | |  |  |
| FRAS_47 (.46.) 1.447 0.204 7.082 0.000 | | | |  |  |
| FRAS_50 (.47.) -0.210 0.152 -1.376 0.169 | | | |  |  |
| D5 =~ | | | |  |  |
| FRAS_12 1.000 | | | |  |  |
| FRAS_35 (.49.) 1.007 0.070 14.322 0.000 | | | |  |  |
| FRAS_42 (.50.) 0.899 0.068 13.139 0.000 | | | |  |  |
| FRAS_44 (.51.) 0.456 0.101 4.494 0.000 | | | |  |  |
| D6 =~ | | | |  |  |
| FRAS_3 1.000 | | | |  |  |
| FRAS_4 (.53.) 1.058 0.065 16.162 0.000 | | | |  |  |
| FRAS_5 (.54.) 1.101 0.073 15.102 0.000 | | | |  |  |
|  |  |  |  |  |  |
| Covariances: |  |  |  |  |  |
| Estimate Std.Err z-value P(>\|z\|) | | | |  |  |
| D1 ~~ | | | |  |  |
| D2 0.070 0.011 6.494 0.000 | | | |  |  |
| D3 0.078 0.011 6.937 0.000 | | | |  |  |
| D4 0.058 0.011 5.293 0.000 | | | |  |  |
| D5 0.085 0.012 7.063 0.000 | | | |  |  |
| D6 0.069 0.010 6.787 0.000 | | | |  |  |
| D2 ~~ | | | |  |  |
| D3 0.084 0.011 7.512 0.000 | | | |  |  |
| D4 0.062 0.011 5.510 0.000 | | | |  |  |
| D5 0.084 0.011 7.417 0.000 | | | |  |  |
| D6 0.075 0.010 7.300 0.000 | | | |  |  |
| D3 ~~ | | | |  |  |
| D4 0.071 0.012 5.848 0.000 | | | |  |  |
| D5 0.098 0.012 8.347 0.000 | | | |  |  |
| D6 0.075 0.010 7.597 0.000 | | | |  |  |
| D4 ~~ | | | |  |  |
| D5 0.071 0.012 5.781 0.000 | | | |  |  |
| D6 0.056 0.010 5.517 0.000 | | | |  |  |
| D5 ~~ | | | |  |  |
| D6 0.082 0.011 7.741 0.000 | | | |  |  |
|  |  |  |  |  |  |
| Intercepts: |  |  |  |  |  |
| Estimate Std.Err z-value P(>\|z\|) | | | |  |  |
| .FRAS_1 2.037 0.032 62.933 0.000 | | | |  |  |
| .FRAS_6 1.931 0.028 69.762 0.000 | | | |  |  |
| .FRAS_7 1.958 0.028 69.452 0.000 | | | |  |  |
| .FRAS_8 1.995 0.033 60.208 0.000 | | | |  |  |
| .FRAS_9 2.116 0.039 53.911 0.000 | | | |  |  |
| .FRAS_10 2.301 0.046 50.280 0.000 | | | |  |  |
| .FRAS_14 1.880 0.025 74.528 0.000 | | | |  |  |
| .FRAS_15 1.944 0.029 66.037 0.000 | | | |  |  |
| .FRAS_16 1.894 0.026 72.000 0.000 | | | |  |  |
| .FRAS_17 1.926 0.026 74.919 0.000 | | | |  |  |
| .FRAS_18 1.917 0.029 66.389 0.000 | | | |  |  |
| .FRAS_20 1.931 0.026 73.187 0.000 | | | |  |  |
| .FRAS_23 2.319 0.038 60.912 0.000 | | | |  |  |
| .FRAS_24 1.926 0.026 73.842 0.000 | | | |  |  |
| .FRAS_25 1.912 0.025 76.046 0.000 | | | |  |  |
| .FRAS_26 1.917 0.025 77.407 0.000 | | | |  |  |
| .FRAS_27 2.148 0.033 65.721 0.000 | | | |  |  |
| .FRAS_28 1.917 0.025 76.509 0.000 | | | |  |  |
| .FRAS_29 1.926 0.026 73.414 0.000 | | | |  |  |
| .FRAS_30 1.903 0.029 66.117 0.000 | | | |  |  |
| .FRAS_40 2.074 0.032 64.413 0.000 | | | |  |  |
| .FRAS_41 1.972 0.028 69.714 0.000 | | | |  |  |
| .FRAS_46 1.995 0.029 68.974 0.000 | | | |  |  |
| .FRAS_48 1.912 0.024 78.134 0.000 | | | |  |  |
| .FRAS_52 1.856 0.025 73.175 0.000 | | | |  |  |
| .FRAS_53 1.917 0.028 68.562 0.000 | | | |  |  |
| .FRAS_54 1.940 0.023 82.931 0.000 | | | |  |  |
| .FRAS_11 1.958 0.030 66.142 0.000 | | | |  |  |
| .FRAS_19 1.940 0.028 70.101 0.000 | | | |  |  |
| .FRAS_31 1.926 0.027 70.323 0.000 | | | |  |  |
| .FRAS_32 2.019 0.030 67.330 0.000 | | | |  |  |
| .FRAS_38 2.505 0.044 57.032 0.000 | | | |  |  |
| .FRAS_39 2.069 0.036 57.719 0.000 | | | |  |  |
| .FRAS_43 2.375 0.041 57.960 0.000 | | | |  |  |
| .FRAS_49 1.926 0.025 75.945 0.000 | | | |  |  |
| .FRAS_13 1.907 0.027 70.162 0.000 | | | |  |  |
| .FRAS_21 1.954 0.030 65.572 0.000 | | | |  |  |
| .FRAS_22 1.894 0.026 73.619 0.000 | | | |  |  |
| .FRAS_34 2.708 0.041 66.838 0.000 | | | |  |  |
| .FRAS_36 1.880 0.029 64.434 0.000 | | | |  |  |
| .FRAS_51 2.259 0.036 63.329 0.000 | | | |  |  |
| .FRAS_2 2.120 0.036 58.758 0.000 | | | |  |  |
| .FRAS_33 2.907 0.035 82.963 0.000 | | | |  |  |
| .FRAS_37 3.116 0.027 117.293 0.000 | | | |  |  |
| .FRAS_45 2.685 0.041 65.427 0.000 | | | |  |  |
| .FRAS_47 1.949 0.030 63.996 0.000 | | | |  |  |
| .FRAS_50 2.736 0.038 71.823 0.000 | | | |  |  |
| .FRAS_12 1.935 0.028 69.542 0.000 | | | |  |  |
| .FRAS_35 1.981 0.030 66.242 0.000 | | | |  |  |
| .FRAS_42 1.995 0.030 65.520 0.000 | | | |  |  |
| .FRAS_44 2.167 0.038 57.075 0.000 | | | |  |  |
| .FRAS_3 1.921 0.025 76.706 0.000 | | | |  |  |
| .FRAS_4 1.926 0.026 75.286 0.000 | | | |  |  |
| .FRAS_5 1.963 0.028 70.025 0.000 | | | |  |  |
|  |  |  |  |  |  |
| Variances: |  |  |  |  |  |
| Estimate Std.Err z-value P(>\|z\|) | | | |  |  |
| .FRAS_1 0.159 0.015 10.312 0.000 | | | |  |  |
| .FRAS_6 0.077 0.008 10.172 0.000 | | | |  |  |
| .FRAS_7 0.072 0.007 10.124 0.000 | | | |  |  |
| .FRAS_8 0.145 0.014 10.269 0.000 | | | |  |  |
| .FRAS_9 0.274 0.026 10.351 0.000 | | | |  |  |
| .FRAS_10 0.443 0.043 10.388 0.000 | | | |  |  |
| .FRAS_14 0.052 0.005 10.070 0.000 | | | |  |  |
| .FRAS_15 0.083 0.008 10.150 0.000 | | | |  |  |
| .FRAS_16 0.042 0.004 9.886 0.000 | | | |  |  |
| .FRAS_17 0.047 0.005 9.990 0.000 | | | |  |  |
| .FRAS_18 0.073 0.007 10.111 0.000 | | | |  |  |
| .FRAS_20 0.040 0.004 9.849 0.000 | | | |  |  |
| .FRAS_23 0.311 0.030 10.391 0.000 | | | |  |  |
| .FRAS_24 0.072 0.007 10.193 0.000 | | | |  |  |
| .FRAS_25 0.050 0.005 10.059 0.000 | | | |  |  |
| .FRAS_26 0.038 0.004 9.914 0.000 | | | |  |  |
| .FRAS_27 0.230 0.022 10.392 0.000 | | | |  |  |
| .FRAS_28 0.066 0.007 10.191 0.000 | | | |  |  |
| .FRAS_29 0.052 0.005 10.033 0.000 | | | |  |  |
| .FRAS_30 0.096 0.009 10.226 0.000 | | | |  |  |
| .FRAS_40 0.162 0.016 10.319 0.000 | | | |  |  |
| .FRAS_41 0.112 0.011 10.287 0.000 | | | |  |  |
| .FRAS_46 0.120 0.012 10.295 0.000 | | | |  |  |
| .FRAS_48 0.061 0.006 10.175 0.000 | | | |  |  |
| .FRAS_52 0.067 0.007 10.185 0.000 | | | |  |  |
| .FRAS_53 0.093 0.009 10.237 0.000 | | | |  |  |
| .FRAS_54 0.056 0.006 10.177 0.000 | | | |  |  |
| .FRAS_11 0.113 0.011 10.061 0.000 | | | |  |  |
| .FRAS_19 0.060 0.007 9.228 0.000 | | | |  |  |
| .FRAS_31 0.072 0.007 9.672 0.000 | | | |  |  |
| .FRAS_32 0.166 0.016 10.325 0.000 | | | |  |  |
| .FRAS_38 0.396 0.038 10.373 0.000 | | | |  |  |
| .FRAS_39 0.253 0.024 10.356 0.000 | | | |  |  |
| .FRAS_43 0.362 0.035 10.392 0.000 | | | |  |  |
| .FRAS_49 0.071 0.007 9.872 0.000 | | | |  |  |
| .FRAS_13 0.073 0.007 10.465 0.000 | | | |  |  |
| .FRAS_21 0.080 0.008 10.377 0.000 | | | |  |  |
| .FRAS_22 0.056 0.005 10.301 0.000 | | | |  |  |
| .FRAS_34 0.354 0.034 10.393 0.000 | | | |  |  |
| .FRAS_36 0.102 0.010 10.566 0.000 | | | |  |  |
| .FRAS_51 0.275 0.026 10.392 0.000 | | | |  |  |
| .FRAS_2 0.226 0.022 10.197 0.000 | | | |  |  |
| .FRAS_33 0.229 0.022 10.269 0.000 | | | |  |  |
| .FRAS_37 0.053 0.007 8.041 0.000 | | | |  |  |
| .FRAS_45 0.357 0.034 10.378 0.000 | | | |  |  |
| .FRAS_47 0.084 0.010 8.855 0.000 | | | |  |  |
| .FRAS_50 0.311 0.030 10.387 0.000 | | | |  |  |
| .FRAS_12 0.066 0.007 9.335 0.000 | | | |  |  |
| .FRAS_35 0.091 0.009 9.823 0.000 | | | |  |  |
| .FRAS_42 0.118 0.012 10.241 0.000 | | | |  |  |
| .FRAS_44 0.290 0.028 10.414 0.000 | | | |  |  |
| .FRAS_3 0.042 0.005 8.082 0.000 | | | |  |  |
| .FRAS_4 0.037 0.005 7.412 0.000 | | | |  |  |
| .FRAS_5 0.057 0.007 8.322 0.000 | | | |  |  |
| D1 0.067 0.014 4.772 0.000 | | | |  |  |
| D2 0.077 0.014 5.608 0.000 | | | |  |  |
| D3 0.087 0.013 6.728 0.000 | | | |  |  |
| D4 0.055 0.015 3.577 0.000 | | | |  |  |
| D5 0.101 0.014 7.058 0.000 | | | |  |  |
| D6 0.093 0.013 7.456 0.000 | | | |  |  |
|  |  |  |  |  |  |
|  |  |  |  |  |  |
| Group 2 [2]: |  |  |  |  |  |
|  |  |  |  |  |  |
| Latent Variables: | |  |  |  |  |
| Estimate Std.Err z-value P(>\|z\|) | | | |  |  |
| D1 =~ | | | |  |  |
| FRAS_1 1.000 | | | |  |  |
| FRAS_6 (.p2.) 1.148 0.125 9.146 0.000 | | | |  |  |
| FRAS_7 (.p3.) 1.222 0.130 9.365 0.000 | | | |  |  |
| FRAS_8 (.p4.) 1.177 0.140 8.389 0.000 | | | |  |  |
| FRAS_9 (.p5.) 0.941 0.151 6.217 0.000 | | | |  |  |
| FRAS_10 (.p6.) 0.382 0.160 2.381 0.017 | | | |  |  |
| FRAS_14 (.p7.) 1.133 0.116 9.790 0.000 | | | |  |  |
| FRAS_15 (.p8.) 1.248 0.134 9.329 0.000 | | | |  |  |
| FRAS_16 (.p9.) 1.270 0.127 10.005 0.000 | | | |  |  |
| FRAS_17 (.10.) 1.199 0.124 9.640 0.000 | | | |  |  |
| FRAS_18 (.11.) 1.263 0.132 9.564 0.000 | | | |  |  |
| FRAS_20 (.12.) 1.286 0.129 9.948 0.000 | | | |  |  |
| FRAS_23 (.13.) 0.193 0.134 1.442 0.149 | | | |  |  |
| FRAS_24 (.14.) 1.057 0.115 9.160 0.000 | | | |  |  |
| FRAS_25 (.15.) 1.137 0.118 9.661 0.000 | | | |  |  |
| FRAS_26 (.16.) 1.186 0.121 9.816 0.000 | | | |  |  |
| FRAS_27 (.17.) 0.120 0.113 1.064 0.287 | | | |  |  |
| FRAS_28 (.18.) 1.018 0.110 9.216 0.000 | | | |  |  |
| FRAS_29 (.19.) 1.202 0.122 9.841 0.000 | | | |  |  |
| FRAS_30 (.20.) 1.113 0.128 8.679 0.000 | | | |  |  |
| FRAS_40 (.21.) 0.961 0.132 7.258 0.000 | | | |  |  |
| FRAS_41 (.22.) 0.957 0.118 8.098 0.000 | | | |  |  |
| FRAS_46 (.23.) 0.952 0.120 7.946 0.000 | | | |  |  |
| FRAS_48 (.24.) 1.012 0.111 9.118 0.000 | | | |  |  |
| FRAS_52 (.25.) 1.037 0.115 9.025 0.000 | | | |  |  |
| FRAS_53 (.26.) 1.062 0.116 9.159 0.000 | | | |  |  |
| FRAS_54 (.27.) 0.965 0.108 8.915 0.000 | | | |  |  |
| D2 =~ | | | |  |  |
| FRAS_11 1.000 | | | |  |  |
| FRAS_19 (.29.) 1.170 0.102 11.477 0.000 | | | |  |  |
| FRAS_31 (.30.) 1.080 0.097 11.164 0.000 | | | |  |  |
| FRAS_32 (.31.) 0.606 0.101 5.985 0.000 | | | |  |  |
| FRAS_38 (.32.) 0.518 0.144 3.590 0.000 | | | |  |  |
| FRAS_39 (.33.) 0.563 0.119 4.729 0.000 | | | |  |  |
| FRAS_43 (.34.) -0.059 0.132 -0.443 0.658 | | | |  |  |
| FRAS_49 (.35.) 0.943 0.091 10.388 0.000 | | | |  |  |
| D3 =~ | | | |  |  |
| FRAS_13 1.000 | | | |  |  |
| FRAS_21 (.37.) 1.136 0.077 14.779 0.000 | | | |  |  |
| FRAS_22 (.38.) 1.000 0.072 13.907 0.000 | | | |  |  |
| FRAS_34 (.39.) 0.075 0.120 0.630 0.529 | | | |  |  |
| FRAS_36 (.40.) 0.969 0.086 11.301 0.000 | | | |  |  |
| FRAS_51 (.41.) 0.028 0.106 0.264 0.792 | | | |  |  |
| D4 =~ | | | |  |  |
| FRAS_2 1.000 | | | |  |  |
| FRAS_33 (.43.) -0.813 0.167 -4.863 0.000 | | | |  |  |
| FRAS_37 (.44.) -1.340 0.186 -7.194 0.000 | | | |  |  |
| FRAS_45 (.45.) -0.356 0.166 -2.145 0.032 | | | |  |  |
| FRAS_47 (.46.) 1.447 0.204 7.082 0.000 | | | |  |  |
| FRAS_50 (.47.) -0.210 0.152 -1.376 0.169 | | | |  |  |
| D5 =~ | | | |  |  |
| FRAS_12 1.000 | | | |  |  |
| FRAS_35 (.49.) 1.007 0.070 14.322 0.000 | | | |  |  |
| FRAS_42 (.50.) 0.899 0.068 13.139 0.000 | | | |  |  |
| FRAS_44 (.51.) 0.456 0.101 4.494 0.000 | | | |  |  |
| D6 =~ | | | |  |  |
| FRAS_3 1.000 | | | |  |  |
| FRAS_4 (.53.) 1.058 0.065 16.162 0.000 | | | |  |  |
| FRAS_5 (.54.) 1.101 0.073 15.102 0.000 | | | |  |  |
|  |  |  |  |  |  |
| Covariances: |  |  |  |  |  |
| Estimate Std.Err z-value P(>\|z\|) | | | |  |  |
| D1 ~~ | | | |  |  |
| D2 0.060 0.013 4.744 0.000 | | | |  |  |
| D3 0.061 0.013 4.720 0.000 | | | |  |  |
| D4 0.040 0.010 4.041 0.000 | | | |  |  |
| D5 0.073 0.015 4.936 0.000 | | | |  |  |
| D6 0.056 0.012 4.573 0.000 | | | |  |  |
| D2 ~~ | | | |  |  |
| D3 0.061 0.013 4.704 0.000 | | | |  |  |
| D4 0.043 0.010 4.149 0.000 | | | |  |  |
| D5 0.078 0.015 5.097 0.000 | | | |  |  |
| D6 0.055 0.012 4.479 0.000 | | | |  |  |
| D3 ~~ | | | |  |  |
| D4 0.041 0.010 3.977 0.000 | | | |  |  |
| D5 0.079 0.016 5.049 0.000 | | | |  |  |
| D6 0.065 0.014 4.789 0.000 | | | |  |  |
| D4 ~~ | | | |  |  |
| D5 0.050 0.012 4.155 0.000 | | | |  |  |
| D6 0.043 0.011 4.068 0.000 | | | |  |  |
| D5 ~~ | | | |  |  |
| D6 0.065 0.014 4.526 0.000 | | | |  |  |
|  |  |  |  |  |  |
| Intercepts: |  |  |  |  |  |
| Estimate Std.Err z-value P(>\|z\|) | | | |  |  |
| .FRAS_1 2.103 0.054 39.281 0.000 | | | |  |  |
| .FRAS_6 1.897 0.048 39.461 0.000 | | | |  |  |
| .FRAS_7 1.971 0.050 39.701 0.000 | | | |  |  |
| .FRAS_8 2.000 0.054 37.070 0.000 | | | |  |  |
| .FRAS_9 2.059 0.069 29.843 0.000 | | | |  |  |
| .FRAS_10 2.368 0.080 29.616 0.000 | | | |  |  |
| .FRAS_14 1.956 0.037 52.184 0.000 | | | |  |  |
| .FRAS_15 1.971 0.048 40.993 0.000 | | | |  |  |
| .FRAS_16 1.941 0.043 45.572 0.000 | | | |  |  |
| .FRAS_17 2.029 0.059 34.624 0.000 | | | |  |  |
| .FRAS_18 1.956 0.045 43.584 0.000 | | | |  |  |
| .FRAS_20 1.941 0.049 39.981 0.000 | | | |  |  |
| .FRAS_23 2.368 0.071 33.529 0.000 | | | |  |  |
| .FRAS_24 1.956 0.041 48.231 0.000 | | | |  |  |
| .FRAS_25 1.971 0.041 47.979 0.000 | | | |  |  |
| .FRAS_26 2.000 0.050 39.687 0.000 | | | |  |  |
| .FRAS_27 2.118 0.056 37.795 0.000 | | | |  |  |
| .FRAS_28 1.971 0.038 51.826 0.000 | | | |  |  |
| .FRAS_29 1.985 0.040 49.768 0.000 | | | |  |  |
| .FRAS_30 1.985 0.056 35.535 0.000 | | | |  |  |
| .FRAS_40 2.132 0.063 33.797 0.000 | | | |  |  |
| .FRAS_41 2.000 0.047 42.561 0.000 | | | |  |  |
| .FRAS_46 1.985 0.048 41.230 0.000 | | | |  |  |
| .FRAS_48 1.985 0.043 46.178 0.000 | | | |  |  |
| .FRAS_52 1.868 0.046 40.326 0.000 | | | |  |  |
| .FRAS_53 1.941 0.037 52.061 0.000 | | | |  |  |
| .FRAS_54 2.000 0.059 33.666 0.000 | | | |  |  |
| .FRAS_11 1.956 0.047 41.360 0.000 | | | |  |  |
| .FRAS_19 1.956 0.048 40.457 0.000 | | | |  |  |
| .FRAS_31 1.941 0.041 47.731 0.000 | | | |  |  |
| .FRAS_32 2.044 0.057 36.059 0.000 | | | |  |  |
| .FRAS_38 2.382 0.081 29.540 0.000 | | | |  |  |
| .FRAS_39 2.147 0.067 32.239 0.000 | | | |  |  |
| .FRAS_43 2.412 0.073 32.959 0.000 | | | |  |  |
| .FRAS_49 2.000 0.044 45.493 0.000 | | | |  |  |
| .FRAS_13 1.971 0.045 43.680 0.000 | | | |  |  |
| .FRAS_21 1.941 0.040 49.062 0.000 | | | |  |  |
| .FRAS_22 1.882 0.044 43.264 0.000 | | | |  |  |
| .FRAS_34 2.721 0.072 37.993 0.000 | | | |  |  |
| .FRAS_36 1.897 0.063 29.920 0.000 | | | |  |  |
| .FRAS_51 2.088 0.065 32.093 0.000 | | | |  |  |
| .FRAS_2 2.118 0.057 36.984 0.000 | | | |  |  |
| .FRAS_33 2.941 0.057 51.748 0.000 | | | |  |  |
| .FRAS_37 3.059 0.047 64.941 0.000 | | | |  |  |
| .FRAS_45 2.897 0.063 46.070 0.000 | | | |  |  |
| .FRAS_47 2.015 0.046 43.357 0.000 | | | |  |  |
| .FRAS_50 2.882 0.061 47.177 0.000 | | | |  |  |
| .FRAS_12 1.941 0.042 46.183 0.000 | | | |  |  |
| .FRAS_35 1.971 0.051 38.509 0.000 | | | |  |  |
| .FRAS_42 2.000 0.044 45.547 0.000 | | | |  |  |
| .FRAS_44 2.368 0.067 35.513 0.000 | | | |  |  |
| .FRAS_3 1.985 0.046 43.356 0.000 | | | |  |  |
| .FRAS_4 2.000 0.043 46.987 0.000 | | | |  |  |
| .FRAS_5 1.985 0.046 42.949 0.000 | | | |  |  |
|  |  |  |  |  |  |
| Variances: |  |  |  |  |  |
| Estimate Std.Err z-value P(>\|z\|) | | | |  |  |
| .FRAS_1 0.139 0.024 5.811 0.000 | | | |  |  |
| .FRAS_6 0.084 0.014 5.786 0.000 | | | |  |  |
| .FRAS_7 0.084 0.015 5.780 0.000 | | | |  |  |
| .FRAS_8 0.121 0.021 5.799 0.000 | | | |  |  |
| .FRAS_9 0.274 0.047 5.822 0.000 | | | |  |  |
| .FRAS_10 0.426 0.073 5.830 0.000 | | | |  |  |
| .FRAS_14 0.024 0.004 5.661 0.000 | | | |  |  |
| .FRAS_15 0.070 0.012 5.767 0.000 | | | |  |  |
| .FRAS_16 0.033 0.006 5.681 0.000 | | | |  |  |
| .FRAS_17 0.153 0.026 5.805 0.000 | | | |  |  |
| .FRAS_18 0.048 0.008 5.731 0.000 | | | |  |  |
| .FRAS_20 0.068 0.012 5.760 0.000 | | | |  |  |
| .FRAS_23 0.337 0.058 5.831 0.000 | | | |  |  |
| .FRAS_24 0.049 0.009 5.765 0.000 | | | |  |  |
| .FRAS_25 0.043 0.007 5.741 0.000 | | | |  |  |
| .FRAS_26 0.094 0.016 5.789 0.000 | | | |  |  |
| .FRAS_27 0.213 0.036 5.831 0.000 | | | |  |  |
| .FRAS_28 0.040 0.007 5.756 0.000 | | | |  |  |
| .FRAS_29 0.028 0.005 5.667 0.000 | | | |  |  |
| .FRAS_30 0.143 0.025 5.807 0.000 | | | |  |  |
| .FRAS_40 0.219 0.038 5.819 0.000 | | | |  |  |
| .FRAS_41 0.099 0.017 5.805 0.000 | | | |  |  |
| .FRAS_46 0.107 0.018 5.807 0.000 | | | |  |  |
| .FRAS_48 0.069 0.012 5.789 0.000 | | | |  |  |
| .FRAS_52 0.086 0.015 5.796 0.000 | | | |  |  |
| .FRAS_53 0.031 0.006 5.723 0.000 | | | |  |  |
| .FRAS_54 0.188 0.032 5.817 0.000 | | | |  |  |
| .FRAS_11 0.096 0.015 6.284 0.000 | | | |  |  |
| .FRAS_19 0.082 0.013 6.334 0.000 | | | |  |  |
| .FRAS_31 0.047 0.008 6.266 0.000 | | | |  |  |
| .FRAS_32 0.198 0.033 5.964 0.000 | | | |  |  |
| .FRAS_38 0.427 0.073 5.879 0.000 | | | |  |  |
| .FRAS_39 0.284 0.048 5.914 0.000 | | | |  |  |
| .FRAS_43 0.364 0.062 5.832 0.000 | | | |  |  |
| .FRAS_49 0.082 0.013 6.296 0.000 | | | |  |  |
| .FRAS_13 0.064 0.012 5.511 0.000 | | | |  |  |
| .FRAS_21 0.011 0.004 2.774 0.006 | | | |  |  |
| .FRAS_22 0.055 0.010 5.453 0.000 | | | |  |  |
| .FRAS_34 0.348 0.060 5.831 0.000 | | | |  |  |
| .FRAS_36 0.204 0.036 5.738 0.000 | | | |  |  |
| .FRAS_51 0.288 0.049 5.831 0.000 | | | |  |  |
| .FRAS_2 0.201 0.034 5.980 0.000 | | | |  |  |
| .FRAS_33 0.205 0.034 5.953 0.000 | | | |  |  |
| .FRAS_37 0.111 0.019 5.747 0.000 | | | |  |  |
| .FRAS_45 0.266 0.045 5.855 0.000 | | | |  |  |
| .FRAS_47 0.101 0.018 5.553 0.000 | | | |  |  |
| .FRAS_50 0.253 0.043 5.840 0.000 | | | |  |  |
| .FRAS_12 0.028 0.006 4.882 0.000 | | | |  |  |
| .FRAS_35 0.084 0.015 5.769 0.000 | | | |  |  |
| .FRAS_42 0.056 0.010 5.710 0.000 | | | |  |  |
| .FRAS_44 0.283 0.048 5.841 0.000 | | | |  |  |
| .FRAS_3 0.076 0.014 5.383 0.000 | | | |  |  |
| .FRAS_4 0.049 0.010 4.891 0.000 | | | |  |  |
| .FRAS_5 0.065 0.013 5.116 0.000 | | | |  |  |
| D1 0.056 0.014 3.903 0.000 | | | |  |  |
| D2 0.056 0.014 3.896 0.000 | | | |  |  |
| D3 0.074 0.016 4.560 0.000 | | | |  |  |
| D4 0.022 0.010 2.287 0.022 | | | |  |  |
| D5 0.092 0.019 4.796 0.000 | | | |  |  |
| D6 0.066 0.016 4.211 0.000 | | | |  |  |
|  |  |  |  |  |  |
| lavaan 0.6-19 ended normally after 502 iterations | | | |  |  |
|  |  |  |  |  |  |
| Estimator ML | | | |  |  |
| Optimization method NLMINB | | | |  |  |
| Number of model parameters 360 | | | |  |  |
| Number of equality constraints 102 | | | |  |  |
|  |  |  |  |  |  |
| Number of observations per group: | | | |  |  |
| 1 216 | | | |  |  |
| 2 68 | | | |  |  |
|  |  |  |  |  |  |
| Model Test User Model: | |  |  |  |  |
|  | | | |  |  |
| Test statistic 7901.838 | | | |  |  |
| Degrees of freedom 2820 | | | |  |  |
| P-value (Chi-square) 0.000 | | | |  |  |
| Test statistic for each group: | | |  |  |  |
| 1 3814.975 | | | |  |  |
| 2 4086.863 | | | |  |  |
|  |  |  |  |  |  |
| Model Test Baseline Model: | |  |  |  |  |
|  |  |  |  |  |  |
| Test statistic 15694.224 | | | |  |  |
| Degrees of freedom 2862 | | | |  |  |
| P-value 0.000 | | | |  |  |
|  |  |  |  |  |  |
| User Model versus Baseline Model: | | |  |  |  |
|  |  |  |  |  |  |
| Comparative Fit Index (CFI) 0.604 | | | |  |  |
| Tucker-Lewis Index (TLI) 0.598 | | | |  |  |
|  |  |  |  |  |  |
| Loglikelihood and Information Criteria: | | |  |  |  |
|  |  |  |  |  |  |
| Loglikelihood user model (H0) -5128.139 | | | |  |  |
| Loglikelihood unrestricted model (H1) -1177.220 | | | |  |  |
|  | | | |  |  |
| Akaike (AIC) 10772.278 | | | |  |  |
| Bayesian (BIC) 11713.713 | | | |  |  |
| Sample-size adjusted Bayesian (SABIC) 10895.586 | | | |  |  |
|  |  |  |  |  |  |
| Root Mean Square Error of Approximation: | | |  |  |  |
|  |  |  |  |  |  |
| RMSEA 0.113 | | | |  |  |
| 90 Percent confidence interval - lower 0.110 | | | |  |  |
| 90 Percent confidence interval - upper 0.116 | | | |  |  |
| P-value H_0: RMSEA <= 0.050 0.000 | | | |  |  |
| P-value H_0: RMSEA >= 0.080 1.000 | | | |  |  |
|  |  |  |  |  |  |
| Standardized Root Mean Square Residual: | | |  |  |  |
|  |  |  |  |  |  |
| SRMR 0.098 | | | |  |  |
|  |  |  |  |  |  |
| Parameter Estimates: | |  |  |  |  |
|  |  |  |  |  |  |
| Standard errors Standard | | | |  |  |
| Information Expected | | | |  |  |
| Information saturated (h1) model Structured | | | |  |  |
|  |  |  |  |  |  |
|  |  |  |  |  |  |
| Group 1 [1]: |  |  |  |  |  |
|  |  |  |  |  |  |
| Latent Variables: | |  |  |  |  |
| Estimate Std.Err z-value P(>\|z\|) | | | |  |  |
| D1 =~ | | | |  |  |
| FRAS_1 1.000 | | | |  |  |
| FRAS_6 (.p2.) 1.138 0.124 9.154 0.000 | | | |  |  |
| FRAS_7 (.p3.) 1.214 0.129 9.394 0.000 | | | |  |  |
| FRAS_8 (.p4.) 1.168 0.139 8.402 0.000 | | | |  |  |
| FRAS_9 (.p5.) 0.926 0.150 6.175 0.000 | | | |  |  |
| FRAS_10 (.p6.) 0.386 0.160 2.415 0.016 | | | |  |  |
| FRAS_14 (.p7.) 1.135 0.115 9.836 0.000 | | | |  |  |
| FRAS_15 (.p8.) 1.242 0.133 9.363 0.000 | | | |  |  |
| FRAS_16 (.p9.) 1.267 0.126 10.053 0.000 | | | |  |  |
| FRAS_17 (.10.) 1.198 0.124 9.683 0.000 | | | |  |  |
| FRAS_18 (.11.) 1.258 0.131 9.604 0.000 | | | |  |  |
| FRAS_20 (.12.) 1.278 0.128 9.987 0.000 | | | |  |  |
| FRAS_23 (.13.) 0.196 0.133 1.471 0.141 | | | |  |  |
| FRAS_24 (.14.) 1.052 0.114 9.196 0.000 | | | |  |  |
| FRAS_25 (.15.) 1.135 0.117 9.707 0.000 | | | |  |  |
| FRAS_26 (.16.) 1.184 0.120 9.861 0.000 | | | |  |  |
| FRAS_27 (.17.) 0.115 0.112 1.025 0.305 | | | |  |  |
| FRAS_28 (.18.) 1.017 0.110 9.260 0.000 | | | |  |  |
| FRAS_29 (.19.) 1.200 0.121 9.888 0.000 | | | |  |  |
| FRAS_30 (.20.) 1.113 0.128 8.720 0.000 | | | |  |  |
| FRAS_40 (.21.) 0.960 0.132 7.288 0.000 | | | |  |  |
| FRAS_41 (.22.) 0.953 0.117 8.124 0.000 | | | |  |  |
| FRAS_46 (.23.) 0.944 0.119 7.949 0.000 | | | |  |  |
| FRAS_48 (.24.) 1.012 0.110 9.163 0.000 | | | |  |  |
| FRAS_52 (.25.) 1.031 0.114 9.052 0.000 | | | |  |  |
| FRAS_53 (.26.) 1.056 0.115 9.191 0.000 | | | |  |  |
| FRAS_54 (.27.) 0.963 0.108 8.952 0.000 | | | |  |  |
| D2 =~ | | | |  |  |
| FRAS_11 1.000 | | | |  |  |
| FRAS_19 (.29.) 1.171 0.102 11.463 0.000 | | | |  |  |
| FRAS_31 (.30.) 1.081 0.097 11.152 0.000 | | | |  |  |
| FRAS_32 (.31.) 0.607 0.101 5.989 0.000 | | | |  |  |
| FRAS_38 (.32.) 0.510 0.145 3.527 0.000 | | | |  |  |
| FRAS_39 (.33.) 0.567 0.119 4.746 0.000 | | | |  |  |
| FRAS_43 (.34.) -0.057 0.132 -0.432 0.666 | | | |  |  |
| FRAS_49 (.35.) 0.949 0.091 10.388 0.000 | | | |  |  |
| D3 =~ | | | |  |  |
| FRAS_13 1.000 | | | |  |  |
| FRAS_21 (.37.) 1.135 0.077 14.720 0.000 | | | |  |  |
| FRAS_22 (.38.) 0.999 0.072 13.869 0.000 | | | |  |  |
| FRAS_34 (.39.) 0.075 0.119 0.624 0.533 | | | |  |  |
| FRAS_36 (.40.) 0.969 0.086 11.286 0.000 | | | |  |  |
| FRAS_51 (.41.) 0.030 0.107 0.283 0.777 | | | |  |  |
| D4 =~ | | | |  |  |
| FRAS_2 1.000 | | | |  |  |
| FRAS_33 (.43.) -0.809 0.168 -4.831 0.000 | | | |  |  |
| FRAS_37 (.44.) -1.350 0.188 -7.172 0.000 | | | |  |  |
| FRAS_45 (.45.) -0.330 0.167 -1.973 0.048 | | | |  |  |
| FRAS_47 (.46.) 1.456 0.206 7.060 0.000 | | | |  |  |
| FRAS_50 (.47.) -0.194 0.153 -1.265 0.206 | | | |  |  |
| D5 =~ | | | |  |  |
| FRAS_12 1.000 | | | |  |  |
| FRAS_35 (.49.) 1.006 0.070 14.316 0.000 | | | |  |  |
| FRAS_42 (.50.) 0.899 0.068 13.139 0.000 | | | |  |  |
| FRAS_44 (.51.) 0.461 0.103 4.488 0.000 | | | |  |  |
| D6 =~ | | | |  |  |
| FRAS_3 1.000 | | | |  |  |
| FRAS_4 (.53.) 1.059 0.065 16.238 0.000 | | | |  |  |
| FRAS_5 (.54.) 1.094 0.072 15.096 0.000 | | | |  |  |
|  |  |  |  |  |  |
| Covariances: |  |  |  |  |  |
| Estimate Std.Err z-value P(>\|z\|) | | | |  |  |
| D1 ~~ | | | |  |  |
| D2 0.070 0.011 6.503 0.000 | | | |  |  |
| D3 0.078 0.011 6.949 0.000 | | | |  |  |
| D4 0.057 0.011 5.289 0.000 | | | |  |  |
| D5 0.085 0.012 7.078 0.000 | | | |  |  |
| D6 0.070 0.010 6.801 0.000 | | | |  |  |
| D2 ~~ | | | |  |  |
| D3 0.084 0.011 7.504 0.000 | | | |  |  |
| D4 0.061 0.011 5.497 0.000 | | | |  |  |
| D5 0.084 0.011 7.413 0.000 | | | |  |  |
| D6 0.075 0.010 7.298 0.000 | | | |  |  |
| D3 ~~ | | | |  |  |
| D4 0.071 0.012 5.832 0.000 | | | |  |  |
| D5 0.098 0.012 8.341 0.000 | | | |  |  |
| D6 0.075 0.010 7.595 0.000 | | | |  |  |
| D4 ~~ | | | |  |  |
| D5 0.071 0.012 5.767 0.000 | | | |  |  |
| D6 0.056 0.010 5.504 0.000 | | | |  |  |
| D5 ~~ | | | |  |  |
| D6 0.082 0.011 7.743 0.000 | | | |  |  |
|  |  |  |  |  |  |
| Intercepts: |  |  |  |  |  |
| Estimate Std.Err z-value P(>\|z\|) | | | |  |  |
| .FRAS_1 (.130) 2.044 0.029 69.787 0.000 | | | |  |  |
| .FRAS_6 (.131) 1.914 0.026 73.005 0.000 | | | |  |  |
| .FRAS_7 (.132) 1.951 0.027 72.447 0.000 | | | |  |  |
| .FRAS_8 (.133) 1.985 0.030 65.507 0.000 | | | |  |  |
| .FRAS_9 (.134) 2.094 0.035 59.470 0.000 | | | |  |  |
| .FRAS_10 (.135) 2.314 0.040 57.931 0.000 | | | |  |  |
| .FRAS_14 (.136) 1.893 0.024 80.480 0.000 | | | |  |  |
| .FRAS_15 (.137) 1.939 0.028 70.044 0.000 | | | |  |  |
| .FRAS_16 (.138) 1.893 0.025 74.660 0.000 | | | |  |  |
| .FRAS_17 (.139) 1.931 0.025 76.044 0.000 | | | |  |  |
| .FRAS_18 (.140) 1.914 0.027 70.852 0.000 | | | |  |  |
| .FRAS_20 (.141) 1.925 0.026 74.531 0.000 | | | |  |  |
| .FRAS_23 (.142) 2.329 0.034 69.296 0.000 | | | |  |  |
| .FRAS_24 (.143) 1.923 0.024 79.891 0.000 | | | |  |  |
| .FRAS_25 (.144) 1.916 0.024 79.912 0.000 | | | |  |  |
| .FRAS_26 (.145) 1.921 0.024 78.716 0.000 | | | |  |  |
| .FRAS_27 (.146) 2.139 0.028 75.635 0.000 | | | |  |  |
| .FRAS_28 (.147) 1.922 0.023 83.451 0.000 | | | |  |  |
| .FRAS_29 (.148) 1.931 0.025 78.260 0.000 | | | |  |  |
| .FRAS_30 (.149) 1.910 0.027 69.471 0.000 | | | |  |  |
| .FRAS_40 (.150) 2.078 0.030 69.348 0.000 | | | |  |  |
| .FRAS_41 (.151) 1.970 0.026 76.248 0.000 | | | |  |  |
| .FRAS_46 (.152) 1.983 0.026 75.324 0.000 | | | |  |  |
| .FRAS_48 (.153) 1.920 0.023 82.412 0.000 | | | |  |  |
| .FRAS_52 (.154) 1.851 0.024 76.680 0.000 | | | |  |  |
| .FRAS_53 (.155) 1.909 0.024 79.017 0.000 | | | |  |  |
| .FRAS_54 (.156) 1.942 0.023 84.672 0.000 | | | |  |  |
| .FRAS_11 (.157) 1.951 0.028 70.905 0.000 | | | |  |  |
| .FRAS_19 (.158) 1.938 0.027 71.850 0.000 | | | |  |  |
| .FRAS_31 (.159) 1.923 0.026 73.867 0.000 | | | |  |  |
| .FRAS_32 (.160) 2.021 0.027 74.110 0.000 | | | |  |  |
| .FRAS_38 (.161) 2.475 0.039 63.343 0.000 | | | |  |  |
| .FRAS_39 (.162) 2.083 0.032 64.705 0.000 | | | |  |  |
| .FRAS_43 (.163) 2.384 0.036 66.637 0.000 | | | |  |  |
| .FRAS_49 (.164) 1.937 0.024 79.811 0.000 | | | |  |  |
| .FRAS_13 (.165) 1.923 0.026 74.357 0.000 | | | |  |  |
| .FRAS_21 (.166) 1.942 0.028 69.887 0.000 | | | |  |  |
| .FRAS_22 (.167) 1.890 0.025 76.438 0.000 | | | |  |  |
| .FRAS_34 (.168) 2.711 0.035 76.856 0.000 | | | |  |  |
| .FRAS_36 (.169) 1.882 0.028 66.933 0.000 | | | |  |  |
| .FRAS_51 (.170) 2.221 0.032 70.385 0.000 | | | |  |  |
| .FRAS_2 (.171) 2.113 0.032 65.022 0.000 | | | |  |  |
| .FRAS_33 (.172) 2.922 0.031 93.776 0.000 | | | |  |  |
| .FRAS_37 (.173) 3.113 0.026 118.718 0.000 | | | |  |  |
| .FRAS_45 (.174) 2.747 0.035 78.193 0.000 | | | |  |  |
| .FRAS_47 (.175) 1.955 0.030 65.722 0.000 | | | |  |  |
| .FRAS_50 (.176) 2.777 0.033 84.995 0.000 | | | |  |  |
| .FRAS_12 (.177) 1.934 0.027 72.438 0.000 | | | |  |  |
| .FRAS_35 (.178) 1.977 0.029 69.319 0.000 | | | |  |  |
| .FRAS_42 (.179) 1.994 0.028 72.456 0.000 | | | |  |  |
| .FRAS_44 (.180) 2.212 0.034 65.398 0.000 | | | |  |  |
| .FRAS_3 (.181) 1.923 0.025 78.053 0.000 | | | |  |  |
| .FRAS_4 (.182) 1.929 0.025 76.406 0.000 | | | |  |  |
| .FRAS_5 (.183) 1.956 0.027 71.655 0.000 | | | |  |  |
|  |  |  |  |  |  |
| Variances: |  |  |  |  |  |
| Estimate Std.Err z-value P(>\|z\|) | | | |  |  |
| .FRAS_1 0.160 0.015 10.311 0.000 | | | |  |  |
| .FRAS_6 0.078 0.008 10.176 0.000 | | | |  |  |
| .FRAS_7 0.072 0.007 10.126 0.000 | | | |  |  |
| .FRAS_8 0.145 0.014 10.270 0.000 | | | |  |  |
| .FRAS_9 0.274 0.026 10.352 0.000 | | | |  |  |
| .FRAS_10 0.443 0.043 10.388 0.000 | | | |  |  |
| .FRAS_14 0.052 0.005 10.068 0.000 | | | |  |  |
| .FRAS_15 0.083 0.008 10.151 0.000 | | | |  |  |
| .FRAS_16 0.042 0.004 9.885 0.000 | | | |  |  |
| .FRAS_17 0.047 0.005 9.988 0.000 | | | |  |  |
| .FRAS_18 0.073 0.007 10.112 0.000 | | | |  |  |
| .FRAS_20 0.040 0.004 9.852 0.000 | | | |  |  |
| .FRAS_23 0.311 0.030 10.391 0.000 | | | |  |  |
| .FRAS_24 0.072 0.007 10.193 0.000 | | | |  |  |
| .FRAS_25 0.050 0.005 10.057 0.000 | | | |  |  |
| .FRAS_26 0.038 0.004 9.912 0.000 | | | |  |  |
| .FRAS_27 0.230 0.022 10.392 0.000 | | | |  |  |
| .FRAS_28 0.066 0.007 10.190 0.000 | | | |  |  |
| .FRAS_29 0.052 0.005 10.032 0.000 | | | |  |  |
| .FRAS_30 0.096 0.009 10.225 0.000 | | | |  |  |
| .FRAS_40 0.162 0.016 10.319 0.000 | | | |  |  |
| .FRAS_41 0.112 0.011 10.287 0.000 | | | |  |  |
| .FRAS_46 0.120 0.012 10.296 0.000 | | | |  |  |
| .FRAS_48 0.061 0.006 10.174 0.000 | | | |  |  |
| .FRAS_52 0.067 0.007 10.186 0.000 | | | |  |  |
| .FRAS_53 0.093 0.009 10.238 0.000 | | | |  |  |
| .FRAS_54 0.056 0.006 10.176 0.000 | | | |  |  |
| .FRAS_11 0.113 0.011 10.063 0.000 | | | |  |  |
| .FRAS_19 0.060 0.007 9.231 0.000 | | | |  |  |
| .FRAS_31 0.072 0.007 9.673 0.000 | | | |  |  |
| .FRAS_32 0.166 0.016 10.325 0.000 | | | |  |  |
| .FRAS_38 0.397 0.038 10.374 0.000 | | | |  |  |
| .FRAS_39 0.253 0.024 10.355 0.000 | | | |  |  |
| .FRAS_43 0.363 0.035 10.392 0.000 | | | |  |  |
| .FRAS_49 0.071 0.007 9.869 0.000 | | | |  |  |
| .FRAS_13 0.073 0.007 10.469 0.000 | | | |  |  |
| .FRAS_21 0.080 0.008 10.382 0.000 | | | |  |  |
| .FRAS_22 0.056 0.005 10.306 0.000 | | | |  |  |
| .FRAS_34 0.354 0.034 10.393 0.000 | | | |  |  |
| .FRAS_36 0.102 0.010 10.569 0.000 | | | |  |  |
| .FRAS_51 0.276 0.027 10.392 0.000 | | | |  |  |
| .FRAS_2 0.226 0.022 10.199 0.000 | | | |  |  |
| .FRAS_33 0.229 0.022 10.271 0.000 | | | |  |  |
| .FRAS_37 0.053 0.007 8.019 0.000 | | | |  |  |
| .FRAS_45 0.361 0.035 10.380 0.000 | | | |  |  |
| .FRAS_47 0.084 0.010 8.846 0.000 | | | |  |  |
| .FRAS_50 0.313 0.030 10.387 0.000 | | | |  |  |
| .FRAS_12 0.066 0.007 9.337 0.000 | | | |  |  |
| .FRAS_35 0.091 0.009 9.825 0.000 | | | |  |  |
| .FRAS_42 0.118 0.012 10.242 0.000 | | | |  |  |
| .FRAS_44 0.292 0.028 10.414 0.000 | | | |  |  |
| .FRAS_3 0.042 0.005 8.070 0.000 | | | |  |  |
| .FRAS_4 0.037 0.005 7.385 0.000 | | | |  |  |
| .FRAS_5 0.057 0.007 8.350 0.000 | | | |  |  |
| D1 0.067 0.014 4.791 0.000 | | | |  |  |
| D2 0.077 0.014 5.602 0.000 | | | |  |  |
| D3 0.087 0.013 6.715 0.000 | | | |  |  |
| D4 0.055 0.015 3.564 0.000 | | | |  |  |
| D5 0.101 0.014 7.058 0.000 | | | |  |  |
| D6 0.094 0.013 7.469 0.000 | | | |  |  |
|  |  |  |  |  |  |
|  |  |  |  |  |  |
| Group 2 [2]: |  |  |  |  |  |
|  |  |  |  |  |  |
| Latent Variables: | |  |  |  |  |
| Estimate Std.Err z-value P(>\|z\|) | | | |  |  |
| D1 =~ | | | |  |  |
| FRAS_1 1.000 | | | |  |  |
| FRAS_6 (.p2.) 1.138 0.124 9.154 0.000 | | | |  |  |
| FRAS_7 (.p3.) 1.214 0.129 9.394 0.000 | | | |  |  |
| FRAS_8 (.p4.) 1.168 0.139 8.402 0.000 | | | |  |  |
| FRAS_9 (.p5.) 0.926 0.150 6.175 0.000 | | | |  |  |
| FRAS_10 (.p6.) 0.386 0.160 2.415 0.016 | | | |  |  |
| FRAS_14 (.p7.) 1.135 0.115 9.836 0.000 | | | |  |  |
| FRAS_15 (.p8.) 1.242 0.133 9.363 0.000 | | | |  |  |
| FRAS_16 (.p9.) 1.267 0.126 10.053 0.000 | | | |  |  |
| FRAS_17 (.10.) 1.198 0.124 9.683 0.000 | | | |  |  |
| FRAS_18 (.11.) 1.258 0.131 9.604 0.000 | | | |  |  |
| FRAS_20 (.12.) 1.278 0.128 9.987 0.000 | | | |  |  |
| FRAS_23 (.13.) 0.196 0.133 1.471 0.141 | | | |  |  |
| FRAS_24 (.14.) 1.052 0.114 9.196 0.000 | | | |  |  |
| FRAS_25 (.15.) 1.135 0.117 9.707 0.000 | | | |  |  |
| FRAS_26 (.16.) 1.184 0.120 9.861 0.000 | | | |  |  |
| FRAS_27 (.17.) 0.115 0.112 1.025 0.305 | | | |  |  |
| FRAS_28 (.18.) 1.017 0.110 9.260 0.000 | | | |  |  |
| FRAS_29 (.19.) 1.200 0.121 9.888 0.000 | | | |  |  |
| FRAS_30 (.20.) 1.113 0.128 8.720 0.000 | | | |  |  |
| FRAS_40 (.21.) 0.960 0.132 7.288 0.000 | | | |  |  |
| FRAS_41 (.22.) 0.953 0.117 8.124 0.000 | | | |  |  |
| FRAS_46 (.23.) 0.944 0.119 7.949 0.000 | | | |  |  |
| FRAS_48 (.24.) 1.012 0.110 9.163 0.000 | | | |  |  |
| FRAS_52 (.25.) 1.031 0.114 9.052 0.000 | | | |  |  |
| FRAS_53 (.26.) 1.056 0.115 9.191 0.000 | | | |  |  |
| FRAS_54 (.27.) 0.963 0.108 8.952 0.000 | | | |  |  |
| D2 =~ | | | |  |  |
| FRAS_11 1.000 | | | |  |  |
| FRAS_19 (.29.) 1.171 0.102 11.463 0.000 | | | |  |  |
| FRAS_31 (.30.) 1.081 0.097 11.152 0.000 | | | |  |  |
| FRAS_32 (.31.) 0.607 0.101 5.989 0.000 | | | |  |  |
| FRAS_38 (.32.) 0.510 0.145 3.527 0.000 | | | |  |  |
| FRAS_39 (.33.) 0.567 0.119 4.746 0.000 | | | |  |  |
| FRAS_43 (.34.) -0.057 0.132 -0.432 0.666 | | | |  |  |
| FRAS_49 (.35.) 0.949 0.091 10.388 0.000 | | | |  |  |
| D3 =~ | | | |  |  |
| FRAS_13 1.000 | | | |  |  |
| FRAS_21 (.37.) 1.135 0.077 14.720 0.000 | | | |  |  |
| FRAS_22 (.38.) 0.999 0.072 13.869 0.000 | | | |  |  |
| FRAS_34 (.39.) 0.075 0.119 0.624 0.533 | | | |  |  |
| FRAS_36 (.40.) 0.969 0.086 11.286 0.000 | | | |  |  |
| FRAS_51 (.41.) 0.030 0.107 0.283 0.777 | | | |  |  |
| D4 =~ | | | |  |  |
| FRAS_2 1.000 | | | |  |  |
| FRAS_33 (.43.) -0.809 0.168 -4.831 0.000 | | | |  |  |
| FRAS_37 (.44.) -1.350 0.188 -7.172 0.000 | | | |  |  |
| FRAS_45 (.45.) -0.330 0.167 -1.973 0.048 | | | |  |  |
| FRAS_47 (.46.) 1.456 0.206 7.060 0.000 | | | |  |  |
| FRAS_50 (.47.) -0.194 0.153 -1.265 0.206 | | | |  |  |
| D5 =~ | | | |  |  |
| FRAS_12 1.000 | | | |  |  |
| FRAS_35 (.49.) 1.006 0.070 14.316 0.000 | | | |  |  |
| FRAS_42 (.50.) 0.899 0.068 13.139 0.000 | | | |  |  |
| FRAS_44 (.51.) 0.461 0.103 4.488 0.000 | | | |  |  |
| D6 =~ | | | |  |  |
| FRAS_3 1.000 | | | |  |  |
| FRAS_4 (.53.) 1.059 0.065 16.238 0.000 | | | |  |  |
| FRAS_5 (.54.) 1.094 0.072 15.096 0.000 | | | |  |  |
|  |  |  |  |  |  |
| Covariances: |  |  |  |  |  |
| Estimate Std.Err z-value P(>\|z\|) | | | |  |  |
| D1 ~~ | | | |  |  |
| D2 0.060 0.013 4.748 0.000 | | | |  |  |
| D3 0.061 0.013 4.720 0.000 | | | |  |  |
| D4 0.040 0.010 4.041 0.000 | | | |  |  |
| D5 0.074 0.015 4.942 0.000 | | | |  |  |
| D6 0.056 0.012 4.576 0.000 | | | |  |  |
| D2 ~~ | | | |  |  |
| D3 0.061 0.013 4.699 0.000 | | | |  |  |
| D4 0.043 0.010 4.146 0.000 | | | |  |  |
| D5 0.078 0.015 5.097 0.000 | | | |  |  |
| D6 0.055 0.012 4.476 0.000 | | | |  |  |
| D3 ~~ | | | |  |  |
| D4 0.041 0.010 3.973 0.000 | | | |  |  |
| D5 0.079 0.016 5.043 0.000 | | | |  |  |
| D6 0.065 0.014 4.790 0.000 | | | |  |  |
| D4 ~~ | | | |  |  |
| D5 0.050 0.012 4.153 0.000 | | | |  |  |
| D6 0.043 0.011 4.063 0.000 | | | |  |  |
| D5 ~~ | | | |  |  |
| D6 0.065 0.014 4.524 0.000 | | | |  |  |
|  |  |  |  |  |  |
| Intercepts: |  |  |  |  |  |
| Estimate Std.Err z-value P(>\|z\|) | | | |  |  |
| .FRAS_1 (.130) 2.044 0.029 69.787 0.000 | | | |  |  |
| .FRAS_6 (.131) 1.914 0.026 73.005 0.000 | | | |  |  |
| .FRAS_7 (.132) 1.951 0.027 72.447 0.000 | | | |  |  |
| .FRAS_8 (.133) 1.985 0.030 65.507 0.000 | | | |  |  |
| .FRAS_9 (.134) 2.094 0.035 59.470 0.000 | | | |  |  |
| .FRAS_10 (.135) 2.314 0.040 57.931 0.000 | | | |  |  |
| .FRAS_14 (.136) 1.893 0.024 80.480 0.000 | | | |  |  |
| .FRAS_15 (.137) 1.939 0.028 70.044 0.000 | | | |  |  |
| .FRAS_16 (.138) 1.893 0.025 74.660 0.000 | | | |  |  |
| .FRAS_17 (.139) 1.931 0.025 76.044 0.000 | | | |  |  |
| .FRAS_18 (.140) 1.914 0.027 70.852 0.000 | | | |  |  |
| .FRAS_20 (.141) 1.925 0.026 74.531 0.000 | | | |  |  |
| .FRAS_23 (.142) 2.329 0.034 69.296 0.000 | | | |  |  |
| .FRAS_24 (.143) 1.923 0.024 79.891 0.000 | | | |  |  |
| .FRAS_25 (.144) 1.916 0.024 79.912 0.000 | | | |  |  |
| .FRAS_26 (.145) 1.921 0.024 78.716 0.000 | | | |  |  |
| .FRAS_27 (.146) 2.139 0.028 75.635 0.000 | | | |  |  |
| .FRAS_28 (.147) 1.922 0.023 83.451 0.000 | | | |  |  |
| .FRAS_29 (.148) 1.931 0.025 78.260 0.000 | | | |  |  |
| .FRAS_30 (.149) 1.910 0.027 69.471 0.000 | | | |  |  |
| .FRAS_40 (.150) 2.078 0.030 69.348 0.000 | | | |  |  |
| .FRAS_41 (.151) 1.970 0.026 76.248 0.000 | | | |  |  |
| .FRAS_46 (.152) 1.983 0.026 75.324 0.000 | | | |  |  |
| .FRAS_48 (.153) 1.920 0.023 82.412 0.000 | | | |  |  |
| .FRAS_52 (.154) 1.851 0.024 76.680 0.000 | | | |  |  |
| .FRAS_53 (.155) 1.909 0.024 79.017 0.000 | | | |  |  |
| .FRAS_54 (.156) 1.942 0.023 84.672 0.000 | | | |  |  |
| .FRAS_11 (.157) 1.951 0.028 70.905 0.000 | | | |  |  |
| .FRAS_19 (.158) 1.938 0.027 71.850 0.000 | | | |  |  |
| .FRAS_31 (.159) 1.923 0.026 73.867 0.000 | | | |  |  |
| .FRAS_32 (.160) 2.021 0.027 74.110 0.000 | | | |  |  |
| .FRAS_38 (.161) 2.475 0.039 63.343 0.000 | | | |  |  |
| .FRAS_39 (.162) 2.083 0.032 64.705 0.000 | | | |  |  |
| .FRAS_43 (.163) 2.384 0.036 66.637 0.000 | | | |  |  |
| .FRAS_49 (.164) 1.937 0.024 79.811 0.000 | | | |  |  |
| .FRAS_13 (.165) 1.923 0.026 74.357 0.000 | | | |  |  |
| .FRAS_21 (.166) 1.942 0.028 69.887 0.000 | | | |  |  |
| .FRAS_22 (.167) 1.890 0.025 76.438 0.000 | | | |  |  |
| .FRAS_34 (.168) 2.711 0.035 76.856 0.000 | | | |  |  |
| .FRAS_36 (.169) 1.882 0.028 66.933 0.000 | | | |  |  |
| .FRAS_51 (.170) 2.221 0.032 70.385 0.000 | | | |  |  |
| .FRAS_2 (.171) 2.113 0.032 65.022 0.000 | | | |  |  |
| .FRAS_33 (.172) 2.922 0.031 93.776 0.000 | | | |  |  |
| .FRAS_37 (.173) 3.113 0.026 118.718 0.000 | | | |  |  |
| .FRAS_45 (.174) 2.747 0.035 78.193 0.000 | | | |  |  |
| .FRAS_47 (.175) 1.955 0.030 65.722 0.000 | | | |  |  |
| .FRAS_50 (.176) 2.777 0.033 84.995 0.000 | | | |  |  |
| .FRAS_12 (.177) 1.934 0.027 72.438 0.000 | | | |  |  |
| .FRAS_35 (.178) 1.977 0.029 69.319 0.000 | | | |  |  |
| .FRAS_42 (.179) 1.994 0.028 72.456 0.000 | | | |  |  |
| .FRAS_44 (.180) 2.212 0.034 65.398 0.000 | | | |  |  |
| .FRAS_3 (.181) 1.923 0.025 78.053 0.000 | | | |  |  |
| .FRAS_4 (.182) 1.929 0.025 76.406 0.000 | | | |  |  |
| .FRAS_5 (.183) 1.956 0.027 71.655 0.000 | | | |  |  |
| D1 0.038 0.035 1.097 0.272 | | | |  |  |
| D2 0.023 0.038 0.601 0.548 | | | |  |  |
| D3 0.003 0.042 0.079 0.937 | | | |  |  |
| D4 0.026 0.031 0.847 0.397 | | | |  |  |
| D5 0.008 0.047 0.167 0.868 | | | |  |  |
| D6 0.052 0.042 1.253 0.210 | | | |  |  |
|  |  |  |  |  |  |
| Variances: |  |  |  |  |  |
| Estimate Std.Err z-value P(>\|z\|) | | | |  |  |
| .FRAS_1 0.139 0.024 5.811 0.000 | | | |  |  |
| .FRAS_6 0.087 0.015 5.789 0.000 | | | |  |  |
| .FRAS_7 0.085 0.015 5.782 0.000 | | | |  |  |
| .FRAS_8 0.121 0.021 5.800 0.000 | | | |  |  |
| .FRAS_9 0.280 0.048 5.823 0.000 | | | |  |  |
| .FRAS_10 0.428 0.073 5.830 0.000 | | | |  |  |
| .FRAS_14 0.024 0.004 5.665 0.000 | | | |  |  |
| .FRAS_15 0.070 0.012 5.768 0.000 | | | |  |  |
| .FRAS_16 0.033 0.006 5.683 0.000 | | | |  |  |
| .FRAS_17 0.156 0.027 5.806 0.000 | | | |  |  |
| .FRAS_18 0.048 0.008 5.734 0.000 | | | |  |  |
| .FRAS_20 0.069 0.012 5.763 0.000 | | | |  |  |
| .FRAS_23 0.338 0.058 5.831 0.000 | | | |  |  |
| .FRAS_24 0.050 0.009 5.767 0.000 | | | |  |  |
| .FRAS_25 0.043 0.007 5.743 0.000 | | | |  |  |
| .FRAS_26 0.095 0.016 5.790 0.000 | | | |  |  |
| .FRAS_27 0.214 0.037 5.831 0.000 | | | |  |  |
| .FRAS_28 0.041 0.007 5.758 0.000 | | | |  |  |
| .FRAS_29 0.028 0.005 5.669 0.000 | | | |  |  |
| .FRAS_30 0.144 0.025 5.807 0.000 | | | |  |  |
| .FRAS_40 0.220 0.038 5.820 0.000 | | | |  |  |
| .FRAS_41 0.099 0.017 5.806 0.000 | | | |  |  |
| .FRAS_46 0.108 0.019 5.808 0.000 | | | |  |  |
| .FRAS_48 0.069 0.012 5.790 0.000 | | | |  |  |
| .FRAS_52 0.086 0.015 5.797 0.000 | | | |  |  |
| .FRAS_53 0.032 0.006 5.726 0.000 | | | |  |  |
| .FRAS_54 0.189 0.032 5.818 0.000 | | | |  |  |
| .FRAS_11 0.096 0.015 6.291 0.000 | | | |  |  |
| .FRAS_19 0.083 0.013 6.343 0.000 | | | |  |  |
| .FRAS_31 0.047 0.008 6.275 0.000 | | | |  |  |
| .FRAS_32 0.198 0.033 5.966 0.000 | | | |  |  |
| .FRAS_38 0.438 0.075 5.877 0.000 | | | |  |  |
| .FRAS_39 0.287 0.048 5.915 0.000 | | | |  |  |
| .FRAS_43 0.365 0.063 5.832 0.000 | | | |  |  |
| .FRAS_49 0.083 0.013 6.303 0.000 | | | |  |  |
| .FRAS_13 0.067 0.012 5.523 0.000 | | | |  |  |
| .FRAS_21 0.011 0.004 2.736 0.006 | | | |  |  |
| .FRAS_22 0.055 0.010 5.453 0.000 | | | |  |  |
| .FRAS_34 0.348 0.060 5.831 0.000 | | | |  |  |
| .FRAS_36 0.204 0.036 5.739 0.000 | | | |  |  |
| .FRAS_51 0.306 0.052 5.831 0.000 | | | |  |  |
| .FRAS_2 0.202 0.034 5.992 0.000 | | | |  |  |
| .FRAS_33 0.207 0.035 5.961 0.000 | | | |  |  |
| .FRAS_37 0.112 0.020 5.759 0.000 | | | |  |  |
| .FRAS_45 0.291 0.050 5.852 0.000 | | | |  |  |
| .FRAS_47 0.103 0.018 5.574 0.000 | | | |  |  |
| .FRAS_50 0.265 0.045 5.839 0.000 | | | |  |  |
| .FRAS_12 0.028 0.006 4.880 0.000 | | | |  |  |
| .FRAS_35 0.085 0.015 5.769 0.000 | | | |  |  |
| .FRAS_42 0.056 0.010 5.710 0.000 | | | |  |  |
| .FRAS_44 0.306 0.052 5.841 0.000 | | | |  |  |
| .FRAS_3 0.077 0.014 5.382 0.000 | | | |  |  |
| .FRAS_4 0.049 0.010 4.882 0.000 | | | |  |  |
| .FRAS_5 0.067 0.013 5.143 0.000 | | | |  |  |
| D1 0.056 0.014 3.913 0.000 | | | |  |  |
| D2 0.056 0.014 3.890 0.000 | | | |  |  |
| D3 0.074 0.016 4.551 0.000 | | | |  |  |
| D4 0.021 0.009 2.248 0.025 | | | |  |  |
| D5 0.092 0.019 4.796 0.000 | | | |  |  |
| D6 0.066 0.016 4.204 0.000 | | | |  |  |
|  |  |  |  |  |  |
| lavaan 0.6-19 ended normally after 468 iterations | | | |  |  |
|  |  |  |  |  |  |
| Estimator ML | | | |  |  |
| Optimization method NLMINB | | | |  |  |
| Number of model parameters 360 | | | |  |  |
| Number of equality constraints 156 | | | |  |  |
|  |  |  |  |  |  |
| Number of observations per group: | | | |  |  |
| 1 216 | | | |  |  |
| 2 68 | | | |  |  |
|  |  |  |  |  |  |
| Model Test User Model: | |  |  |  |  |
|  | | | |  |  |
| Test statistic 8194.037 | | | |  |  |
| Degrees of freedom 2874 | | | |  |  |
| P-value (Chi-square) 0.000 | | | |  |  |
| Test statistic for each group: | | |  |  |  |
| 1 3894.782 | | | |  |  |
| 2 4299.255 | | | |  |  |
|  |  |  |  |  |  |
| Model Test Baseline Model: | |  |  |  |  |
|  |  |  |  |  |  |
| Test statistic 15694.224 | | | |  |  |
| Degrees of freedom 2862 | | | |  |  |
| P-value 0.000 | | | |  |  |
|  |  |  |  |  |  |
| User Model versus Baseline Model: | | |  |  |  |
|  |  |  |  |  |  |
| Comparative Fit Index (CFI) 0.585 | | | |  |  |
| Tucker-Lewis Index (TLI) 0.587 | | | |  |  |
|  |  |  |  |  |  |
| Loglikelihood and Information Criteria: | | |  |  |  |
|  |  |  |  |  |  |
| Loglikelihood user model (H0) -5274.239 | | | |  |  |
| Loglikelihood unrestricted model (H1) -1177.220 | | | |  |  |
|  | | | |  |  |
| Akaike (AIC) 10956.477 | | | |  |  |
| Bayesian (BIC) 11700.868 | | | |  |  |
| Sample-size adjusted Bayesian (SABIC) 11053.977 | | | |  |  |
|  |  |  |  |  |  |
| Root Mean Square Error of Approximation: | | |  |  |  |
|  |  |  |  |  |  |
| RMSEA 0.114 | | | |  |  |
| 90 Percent confidence interval - lower 0.111 | | | |  |  |
| 90 Percent confidence interval - upper 0.117 | | | |  |  |
| P-value H_0: RMSEA <= 0.050 0.000 | | | |  |  |
| P-value H_0: RMSEA >= 0.080 1.000 | | | |  |  |
|  |  |  |  |  |  |
| Standardized Root Mean Square Residual: | | |  |  |  |
|  |  |  |  |  |  |
| SRMR 0.097 | | | |  |  |
|  |  |  |  |  |  |
| Parameter Estimates: | |  |  |  |  |
|  |  |  |  |  |  |
| Standard errors Standard | | | |  |  |
| Information Expected | | | |  |  |
| Information saturated (h1) model Structured | | | |  |  |
|  |  |  |  |  |  |
|  |  |  |  |  |  |
| Group 1 [1]: |  |  |  |  |  |
|  |  |  |  |  |  |
| Latent Variables: | |  |  |  |  |
| Estimate Std.Err z-value P(>\|z\|) | | | |  |  |
| D1 =~ | | | |  |  |
| FRAS_1 1.000 | | | |  |  |
| FRAS_6 (.p2.) 1.152 0.126 9.130 0.000 | | | |  |  |
| FRAS_7 (.p3.) 1.228 0.132 9.338 0.000 | | | |  |  |
| FRAS_8 (.p4.) 1.189 0.142 8.395 0.000 | | | |  |  |
| FRAS_9 (.p5.) 0.938 0.152 6.179 0.000 | | | |  |  |
| FRAS_10 (.p6.) 0.400 0.161 2.483 0.013 | | | |  |  |
| FRAS_14 (.p7.) 1.126 0.117 9.657 0.000 | | | |  |  |
| FRAS_15 (.p8.) 1.254 0.135 9.302 0.000 | | | |  |  |
| FRAS_16 (.p9.) 1.264 0.127 9.946 0.000 | | | |  |  |
| FRAS_17 (.10.) 1.210 0.130 9.329 0.000 | | | |  |  |
| FRAS_18 (.11.) 1.256 0.132 9.512 0.000 | | | |  |  |
| FRAS_20 (.12.) 1.292 0.131 9.863 0.000 | | | |  |  |
| FRAS_23 (.13.) 0.210 0.135 1.560 0.119 | | | |  |  |
| FRAS_24 (.14.) 1.072 0.117 9.153 0.000 | | | |  |  |
| FRAS_25 (.15.) 1.137 0.118 9.612 0.000 | | | |  |  |
| FRAS_26 (.16.) 1.198 0.124 9.652 0.000 | | | |  |  |
| FRAS_27 (.17.) 0.107 0.113 0.950 0.342 | | | |  |  |
| FRAS_28 (.18.) 1.039 0.113 9.204 0.000 | | | |  |  |
| FRAS_29 (.19.) 1.215 0.124 9.781 0.000 | | | |  |  |
| FRAS_30 (.20.) 1.106 0.129 8.590 0.000 | | | |  |  |
| FRAS_40 (.21.) 0.974 0.134 7.252 0.000 | | | |  |  |
| FRAS_41 (.22.) 0.972 0.119 8.132 0.000 | | | |  |  |
| FRAS_46 (.23.) 0.962 0.121 7.964 0.000 | | | |  |  |
| FRAS_48 (.24.) 1.016 0.112 9.087 0.000 | | | |  |  |
| FRAS_52 (.25.) 1.050 0.117 9.001 0.000 | | | |  |  |
| FRAS_53 (.26.) 0.989 0.114 8.693 0.000 | | | |  |  |
| FRAS_54 (.27.) 0.918 0.111 8.286 0.000 | | | |  |  |
| D2 =~ | | | |  |  |
| FRAS_11 1.000 | | | |  |  |
| FRAS_19 (.29.) 1.176 0.105 11.223 0.000 | | | |  |  |
| FRAS_31 (.30.) 1.110 0.099 11.163 0.000 | | | |  |  |
| FRAS_32 (.31.) 0.619 0.103 5.998 0.000 | | | |  |  |
| FRAS_38 (.32.) 0.520 0.147 3.540 0.000 | | | |  |  |
| FRAS_39 (.33.) 0.585 0.121 4.817 0.000 | | | |  |  |
| FRAS_43 (.34.) -0.018 0.134 -0.132 0.895 | | | |  |  |
| FRAS_49 (.35.) 0.965 0.093 10.360 0.000 | | | |  |  |
| D3 =~ | | | |  |  |
| FRAS_13 1.000 | | | |  |  |
| FRAS_21 (.37.) 1.041 0.075 13.878 0.000 | | | |  |  |
| FRAS_22 (.38.) 0.983 0.070 14.027 0.000 | | | |  |  |
| FRAS_34 (.39.) 0.104 0.118 0.875 0.382 | | | |  |  |
| FRAS_36 (.40.) 0.907 0.085 10.700 0.000 | | | |  |  |
| FRAS_51 (.41.) 0.062 0.106 0.581 0.561 | | | |  |  |
| D4 =~ | | | |  |  |
| FRAS_2 1.000 | | | |  |  |
| FRAS_33 (.43.) -0.781 0.158 -4.936 0.000 | | | |  |  |
| FRAS_37 (.44.) -1.225 0.167 -7.318 0.000 | | | |  |  |
| FRAS_45 (.45.) -0.362 0.162 -2.236 0.025 | | | |  |  |
| FRAS_47 (.46.) 1.394 0.190 7.336 0.000 | | | |  |  |
| FRAS_50 (.47.) -0.194 0.148 -1.313 0.189 | | | |  |  |
| D5 =~ | | | |  |  |
| FRAS_12 1.000 | | | |  |  |
| FRAS_35 (.49.) 0.988 0.070 14.141 0.000 | | | |  |  |
| FRAS_42 (.50.) 0.871 0.070 12.492 0.000 | | | |  |  |
| FRAS_44 (.51.) 0.455 0.101 4.494 0.000 | | | |  |  |
| D6 =~ | | | |  |  |
| FRAS_3 1.000 | | | |  |  |
| FRAS_4 (.53.) 1.058 0.068 15.561 0.000 | | | |  |  |
| FRAS_5 (.54.) 1.109 0.075 14.761 0.000 | | | |  |  |
|  |  |  |  |  |  |
| Covariances: |  |  |  |  |  |
| Estimate Std.Err z-value P(>\|z\|) | | | |  |  |
| D1 ~~ | | | |  |  |
| D2 0.069 0.011 6.471 0.000 | | | |  |  |
| D3 0.081 0.012 6.942 0.000 | | | |  |  |
| D4 0.061 0.011 5.434 0.000 | | | |  |  |
| D5 0.085 0.012 7.064 0.000 | | | |  |  |
| D6 0.069 0.010 6.754 0.000 | | | |  |  |
| D2 ~~ | | | |  |  |
| D3 0.086 0.011 7.491 0.000 | | | |  |  |
| D4 0.064 0.011 5.670 0.000 | | | |  |  |
| D5 0.084 0.011 7.409 0.000 | | | |  |  |
| D6 0.074 0.010 7.241 0.000 | | | |  |  |
| D3 ~~ | | | |  |  |
| D4 0.077 0.013 6.037 0.000 | | | |  |  |
| D5 0.103 0.012 8.381 0.000 | | | |  |  |
| D6 0.079 0.010 7.601 0.000 | | | |  |  |
| D4 ~~ | | | |  |  |
| D5 0.075 0.013 5.967 0.000 | | | |  |  |
| D6 0.060 0.011 5.680 0.000 | | | |  |  |
| D5 ~~ | | | |  |  |
| D6 0.084 0.011 7.745 0.000 | | | |  |  |
|  |  |  |  |  |  |
| Intercepts: |  |  |  |  |  |
| Estimate Std.Err z-value P(>\|z\|) | | | |  |  |
| .FRAS_1 (.130) 2.044 0.029 69.795 0.000 | | | |  |  |
| .FRAS_6 (.131) 1.912 0.026 72.913 0.000 | | | |  |  |
| .FRAS_7 (.132) 1.950 0.027 72.108 0.000 | | | |  |  |
| .FRAS_8 (.133) 1.986 0.030 65.210 0.000 | | | |  |  |
| .FRAS_9 (.134) 2.094 0.035 59.396 0.000 | | | |  |  |
| .FRAS_10 (.135) 2.313 0.040 57.899 0.000 | | | |  |  |
| .FRAS_14 (.136) 1.888 0.024 80.105 0.000 | | | |  |  |
| .FRAS_15 (.137) 1.939 0.028 69.854 0.000 | | | |  |  |
| .FRAS_16 (.138) 1.893 0.025 74.967 0.000 | | | |  |  |
| .FRAS_17 (.139) 1.940 0.027 72.696 0.000 | | | |  |  |
| .FRAS_18 (.140) 1.915 0.027 71.195 0.000 | | | |  |  |
| .FRAS_20 (.141) 1.921 0.026 73.409 0.000 | | | |  |  |
| .FRAS_23 (.142) 2.329 0.034 69.221 0.000 | | | |  |  |
| .FRAS_24 (.143) 1.923 0.024 79.114 0.000 | | | |  |  |
| .FRAS_25 (.144) 1.916 0.024 79.890 0.000 | | | |  |  |
| .FRAS_26 (.145) 1.926 0.025 76.764 0.000 | | | |  |  |
| .FRAS_27 (.146) 2.140 0.028 75.652 0.000 | | | |  |  |
| .FRAS_28 (.147) 1.920 0.023 82.165 0.000 | | | |  |  |
| .FRAS_29 (.148) 1.929 0.025 77.342 0.000 | | | |  |  |
| .FRAS_30 (.149) 1.912 0.027 69.679 0.000 | | | |  |  |
| .FRAS_40 (.150) 2.079 0.030 68.923 0.000 | | | |  |  |
| .FRAS_41 (.151) 1.970 0.026 75.845 0.000 | | | |  |  |
| .FRAS_46 (.152) 1.984 0.026 75.030 0.000 | | | |  |  |
| .FRAS_48 (.153) 1.920 0.023 82.478 0.000 | | | |  |  |
| .FRAS_52 (.154) 1.850 0.024 75.833 0.000 | | | |  |  |
| .FRAS_53 (.155) 1.914 0.024 79.210 0.000 | | | |  |  |
| .FRAS_54 (.156) 1.946 0.024 81.261 0.000 | | | |  |  |
| .FRAS_11 (.157) 1.952 0.027 71.115 0.000 | | | |  |  |
| .FRAS_19 (.158) 1.937 0.027 71.071 0.000 | | | |  |  |
| .FRAS_31 (.159) 1.923 0.026 74.320 0.000 | | | |  |  |
| .FRAS_32 (.160) 2.021 0.027 73.855 0.000 | | | |  |  |
| .FRAS_38 (.161) 2.472 0.039 63.211 0.000 | | | |  |  |
| .FRAS_39 (.162) 2.085 0.032 64.596 0.000 | | | |  |  |
| .FRAS_43 (.163) 2.384 0.036 66.639 0.000 | | | |  |  |
| .FRAS_49 (.164) 1.938 0.024 79.674 0.000 | | | |  |  |
| .FRAS_13 (.165) 1.920 0.027 72.376 0.000 | | | |  |  |
| .FRAS_21 (.166) 1.948 0.027 72.141 0.000 | | | |  |  |
| .FRAS_22 (.167) 1.888 0.025 74.623 0.000 | | | |  |  |
| .FRAS_34 (.168) 2.711 0.035 76.813 0.000 | | | |  |  |
| .FRAS_36 (.169) 1.881 0.028 66.067 0.000 | | | |  |  |
| .FRAS_51 (.170) 2.218 0.032 70.204 0.000 | | | |  |  |
| .FRAS_2 (.171) 2.112 0.033 64.986 0.000 | | | |  |  |
| .FRAS_33 (.172) 2.921 0.031 94.139 0.000 | | | |  |  |
| .FRAS_37 (.173) 3.111 0.026 118.156 0.000 | | | |  |  |
| .FRAS_45 (.174) 2.739 0.035 77.579 0.000 | | | |  |  |
| .FRAS_47 (.175) 1.955 0.030 65.606 0.000 | | | |  |  |
| .FRAS_50 (.176) 2.773 0.033 84.632 0.000 | | | |  |  |
| .FRAS_12 (.177) 1.934 0.027 71.914 0.000 | | | |  |  |
| .FRAS_35 (.178) 1.977 0.029 68.559 0.000 | | | |  |  |
| .FRAS_42 (.179) 1.995 0.028 72.382 0.000 | | | |  |  |
| .FRAS_44 (.180) 2.214 0.034 65.301 0.000 | | | |  |  |
| .FRAS_3 (.181) 1.924 0.025 77.510 0.000 | | | |  |  |
| .FRAS_4 (.182) 1.930 0.025 76.603 0.000 | | | |  |  |
| .FRAS_5 (.183) 1.954 0.027 71.635 0.000 | | | |  |  |
|  |  |  |  |  |  |
| Variances: |  |  |  |  |  |
| Estimate Std.Err z-value P(>\|z\|) | | | |  |  |
| .FRAS_1 (.70.) 0.155 0.013 11.826 0.000 | | | |  |  |
| .FRAS_6 (.71.) 0.078 0.007 11.678 0.000 | | | |  |  |
| .FRAS_7 (.72.) 0.075 0.006 11.632 0.000 | | | |  |  |
| .FRAS_8 (.73.) 0.138 0.012 11.774 0.000 | | | |  |  |
| .FRAS_9 (.74.) 0.275 0.023 11.872 0.000 | | | |  |  |
| .FRAS_10 (.75.) 0.439 0.037 11.911 0.000 | | | |  |  |
| .FRAS_14 (.76.) 0.046 0.004 11.526 0.000 | | | |  |  |
| .FRAS_15 (.77.) 0.080 0.007 11.641 0.000 | | | |  |  |
| .FRAS_16 (.78.) 0.040 0.004 11.352 0.000 | | | |  |  |
| .FRAS_17 (.79.) 0.073 0.006 11.634 0.000 | | | |  |  |
| .FRAS_18 (.80.) 0.066 0.006 11.581 0.000 | | | |  |  |
| .FRAS_20 (.81.) 0.047 0.004 11.414 0.000 | | | |  |  |
| .FRAS_23 (.82.) 0.317 0.027 11.914 0.000 | | | |  |  |
| .FRAS_24 (.83.) 0.066 0.006 11.674 0.000 | | | |  |  |
| .FRAS_25 (.84.) 0.049 0.004 11.545 0.000 | | | |  |  |
| .FRAS_26 (.85.) 0.052 0.005 11.528 0.000 | | | |  |  |
| .FRAS_27 (.86.) 0.226 0.019 11.916 0.000 | | | |  |  |
| .FRAS_28 (.87.) 0.060 0.005 11.663 0.000 | | | |  |  |
| .FRAS_29 (.88.) 0.046 0.004 11.465 0.000 | | | |  |  |
| .FRAS_30 (.89.) 0.106 0.009 11.755 0.000 | | | |  |  |
| .FRAS_40 (.90.) 0.175 0.015 11.841 0.000 | | | |  |  |
| .FRAS_41 (.91.) 0.108 0.009 11.795 0.000 | | | |  |  |
| .FRAS_46 (.92.) 0.117 0.010 11.806 0.000 | | | |  |  |
| .FRAS_48 (.93.) 0.063 0.005 11.686 0.000 | | | |  |  |
| .FRAS_52 (.94.) 0.072 0.006 11.701 0.000 | | | |  |  |
| .FRAS_53 (.95.) 0.079 0.007 11.744 0.000 | | | |  |  |
| .FRAS_54 (.96.) 0.088 0.007 11.783 0.000 | | | |  |  |
| .FRAS_11 (.97.) 0.111 0.009 11.862 0.000 | | | |  |  |
| .FRAS_19 (.98.) 0.069 0.006 11.400 0.000 | | | |  |  |
| .FRAS_31 (.99.) 0.063 0.006 11.438 0.000 | | | |  |  |
| .FRAS_32 (.100) 0.173 0.015 11.934 0.000 | | | |  |  |
| .FRAS_38 (.101) 0.406 0.034 11.924 0.000 | | | |  |  |
| .FRAS_39 (.102) 0.260 0.022 11.929 0.000 | | | |  |  |
| .FRAS_43 (.103) 0.363 0.030 11.916 0.000 | | | |  |  |
| .FRAS_49 (.104) 0.072 0.006 11.743 0.000 | | | |  |  |
| .FRAS_13 (.105) 0.070 0.006 11.609 0.000 | | | |  |  |
| .FRAS_21 (.106) 0.066 0.006 11.480 0.000 | | | |  |  |
| .FRAS_22 (.107) 0.056 0.005 11.427 0.000 | | | |  |  |
| .FRAS_34 (.108) 0.352 0.030 11.917 0.000 | | | |  |  |
| .FRAS_36 (.109) 0.123 0.010 11.927 0.000 | | | |  |  |
| .FRAS_51 (.110) 0.283 0.024 11.917 0.000 | | | |  |  |
| .FRAS_2 (.111) 0.217 0.018 11.809 0.000 | | | |  |  |
| .FRAS_33 (.112) 0.223 0.019 11.870 0.000 | | | |  |  |
| .FRAS_37 (.113) 0.072 0.007 10.487 0.000 | | | |  |  |
| .FRAS_45 (.114) 0.343 0.029 11.913 0.000 | | | |  |  |
| .FRAS_47 (.115) 0.091 0.009 10.423 0.000 | | | |  |  |
| .FRAS_50 (.116) 0.301 0.025 11.915 0.000 | | | |  |  |
| .FRAS_12 (.117) 0.056 0.005 10.535 0.000 | | | |  |  |
| .FRAS_35 (.118) 0.091 0.008 11.435 0.000 | | | |  |  |
| .FRAS_42 (.119) 0.102 0.009 11.760 0.000 | | | |  |  |
| .FRAS_44 (.120) 0.296 0.025 11.944 0.000 | | | |  |  |
| .FRAS_3 (.121) 0.050 0.005 9.705 0.000 | | | |  |  |
| .FRAS_4 (.122) 0.041 0.005 8.801 0.000 | | | |  |  |
| .FRAS_5 (.123) 0.058 0.006 9.557 0.000 | | | |  |  |
| D1 0.067 0.014 4.758 0.000 | | | |  |  |
| D2 0.075 0.013 5.550 0.000 | | | |  |  |
| D3 0.095 0.014 6.829 0.000 | | | |  |  |
| D4 0.058 0.016 3.711 0.000 | | | |  |  |
| D5 0.107 0.015 7.310 0.000 | | | |  |  |
| D6 0.091 0.013 7.213 0.000 | | | |  |  |
|  |  |  |  |  |  |
|  |  |  |  |  |  |
| Group 2 [2]: |  |  |  |  |  |
|  |  |  |  |  |  |
| Latent Variables: | |  |  |  |  |
| Estimate Std.Err z-value P(>\|z\|) | | | |  |  |
| D1 =~ | | | |  |  |
| FRAS_1 1.000 | | | |  |  |
| FRAS_6 (.p2.) 1.152 0.126 9.130 0.000 | | | |  |  |
| FRAS_7 (.p3.) 1.228 0.132 9.338 0.000 | | | |  |  |
| FRAS_8 (.p4.) 1.189 0.142 8.395 0.000 | | | |  |  |
| FRAS_9 (.p5.) 0.938 0.152 6.179 0.000 | | | |  |  |
| FRAS_10 (.p6.) 0.400 0.161 2.483 0.013 | | | |  |  |
| FRAS_14 (.p7.) 1.126 0.117 9.657 0.000 | | | |  |  |
| FRAS_15 (.p8.) 1.254 0.135 9.302 0.000 | | | |  |  |
| FRAS_16 (.p9.) 1.264 0.127 9.946 0.000 | | | |  |  |
| FRAS_17 (.10.) 1.210 0.130 9.329 0.000 | | | |  |  |
| FRAS_18 (.11.) 1.256 0.132 9.512 0.000 | | | |  |  |
| FRAS_20 (.12.) 1.292 0.131 9.863 0.000 | | | |  |  |
| FRAS_23 (.13.) 0.210 0.135 1.560 0.119 | | | |  |  |
| FRAS_24 (.14.) 1.072 0.117 9.153 0.000 | | | |  |  |
| FRAS_25 (.15.) 1.137 0.118 9.612 0.000 | | | |  |  |
| FRAS_26 (.16.) 1.198 0.124 9.652 0.000 | | | |  |  |
| FRAS_27 (.17.) 0.107 0.113 0.950 0.342 | | | |  |  |
| FRAS_28 (.18.) 1.039 0.113 9.204 0.000 | | | |  |  |
| FRAS_29 (.19.) 1.215 0.124 9.781 0.000 | | | |  |  |
| FRAS_30 (.20.) 1.106 0.129 8.590 0.000 | | | |  |  |
| FRAS_40 (.21.) 0.974 0.134 7.252 0.000 | | | |  |  |
| FRAS_41 (.22.) 0.972 0.119 8.132 0.000 | | | |  |  |
| FRAS_46 (.23.) 0.962 0.121 7.964 0.000 | | | |  |  |
| FRAS_48 (.24.) 1.016 0.112 9.087 0.000 | | | |  |  |
| FRAS_52 (.25.) 1.050 0.117 9.001 0.000 | | | |  |  |
| FRAS_53 (.26.) 0.989 0.114 8.693 0.000 | | | |  |  |
| FRAS_54 (.27.) 0.918 0.111 8.286 0.000 | | | |  |  |
| D2 =~ | | | |  |  |
| FRAS_11 1.000 | | | |  |  |
| FRAS_19 (.29.) 1.176 0.105 11.223 0.000 | | | |  |  |
| FRAS_31 (.30.) 1.110 0.099 11.163 0.000 | | | |  |  |
| FRAS_32 (.31.) 0.619 0.103 5.998 0.000 | | | |  |  |
| FRAS_38 (.32.) 0.520 0.147 3.540 0.000 | | | |  |  |
| FRAS_39 (.33.) 0.585 0.121 4.817 0.000 | | | |  |  |
| FRAS_43 (.34.) -0.018 0.134 -0.132 0.895 | | | |  |  |
| FRAS_49 (.35.) 0.965 0.093 10.360 0.000 | | | |  |  |
| D3 =~ | | | |  |  |
| FRAS_13 1.000 | | | |  |  |
| FRAS_21 (.37.) 1.041 0.075 13.878 0.000 | | | |  |  |
| FRAS_22 (.38.) 0.983 0.070 14.027 0.000 | | | |  |  |
| FRAS_34 (.39.) 0.104 0.118 0.875 0.382 | | | |  |  |
| FRAS_36 (.40.) 0.907 0.085 10.700 0.000 | | | |  |  |
| FRAS_51 (.41.) 0.062 0.106 0.581 0.561 | | | |  |  |
| D4 =~ | | | |  |  |
| FRAS_2 1.000 | | | |  |  |
| FRAS_33 (.43.) -0.781 0.158 -4.936 0.000 | | | |  |  |
| FRAS_37 (.44.) -1.225 0.167 -7.318 0.000 | | | |  |  |
| FRAS_45 (.45.) -0.362 0.162 -2.236 0.025 | | | |  |  |
| FRAS_47 (.46.) 1.394 0.190 7.336 0.000 | | | |  |  |
| FRAS_50 (.47.) -0.194 0.148 -1.313 0.189 | | | |  |  |
| D5 =~ | | | |  |  |
| FRAS_12 1.000 | | | |  |  |
| FRAS_35 (.49.) 0.988 0.070 14.141 0.000 | | | |  |  |
| FRAS_42 (.50.) 0.871 0.070 12.492 0.000 | | | |  |  |
| FRAS_44 (.51.) 0.455 0.101 4.494 0.000 | | | |  |  |
| D6 =~ | | | |  |  |
| FRAS_3 1.000 | | | |  |  |
| FRAS_4 (.53.) 1.058 0.068 15.561 0.000 | | | |  |  |
| FRAS_5 (.54.) 1.109 0.075 14.761 0.000 | | | |  |  |
|  |  |  |  |  |  |
| Covariances: |  |  |  |  |  |
| Estimate Std.Err z-value P(>\|z\|) | | | |  |  |
| D1 ~~ | | | |  |  |
| D2 0.059 0.013 4.706 0.000 | | | |  |  |
| D3 0.057 0.012 4.670 0.000 | | | |  |  |
| D4 0.039 0.010 4.034 0.000 | | | |  |  |
| D5 0.072 0.015 4.859 0.000 | | | |  |  |
| D6 0.055 0.012 4.566 0.000 | | | |  |  |
| D2 ~~ | | | |  |  |
| D3 0.058 0.012 4.654 0.000 | | | |  |  |
| D4 0.044 0.011 4.151 0.000 | | | |  |  |
| D5 0.079 0.016 5.044 0.000 | | | |  |  |
| D6 0.054 0.012 4.435 0.000 | | | |  |  |
| D3 ~~ | | | |  |  |
| D4 0.038 0.010 3.901 0.000 | | | |  |  |
| D5 0.078 0.015 5.058 0.000 | | | |  |  |
| D6 0.055 0.012 4.462 0.000 | | | |  |  |
| D4 ~~ | | | |  |  |
| D5 0.052 0.012 4.173 0.000 | | | |  |  |
| D6 0.042 0.010 4.046 0.000 | | | |  |  |
| D5 ~~ | | | |  |  |
| D6 0.065 0.015 4.480 0.000 | | | |  |  |
|  |  |  |  |  |  |
| Intercepts: |  |  |  |  |  |
| Estimate Std.Err z-value P(>\|z\|) | | | |  |  |
| .FRAS_1 (.130) 2.044 0.029 69.795 0.000 | | | |  |  |
| .FRAS_6 (.131) 1.912 0.026 72.913 0.000 | | | |  |  |
| .FRAS_7 (.132) 1.950 0.027 72.108 0.000 | | | |  |  |
| .FRAS_8 (.133) 1.986 0.030 65.210 0.000 | | | |  |  |
| .FRAS_9 (.134) 2.094 0.035 59.396 0.000 | | | |  |  |
| .FRAS_10 (.135) 2.313 0.040 57.899 0.000 | | | |  |  |
| .FRAS_14 (.136) 1.888 0.024 80.105 0.000 | | | |  |  |
| .FRAS_15 (.137) 1.939 0.028 69.854 0.000 | | | |  |  |
| .FRAS_16 (.138) 1.893 0.025 74.967 0.000 | | | |  |  |
| .FRAS_17 (.139) 1.940 0.027 72.696 0.000 | | | |  |  |
| .FRAS_18 (.140) 1.915 0.027 71.195 0.000 | | | |  |  |
| .FRAS_20 (.141) 1.921 0.026 73.409 0.000 | | | |  |  |
| .FRAS_23 (.142) 2.329 0.034 69.221 0.000 | | | |  |  |
| .FRAS_24 (.143) 1.923 0.024 79.114 0.000 | | | |  |  |
| .FRAS_25 (.144) 1.916 0.024 79.890 0.000 | | | |  |  |
| .FRAS_26 (.145) 1.926 0.025 76.764 0.000 | | | |  |  |
| .FRAS_27 (.146) 2.140 0.028 75.652 0.000 | | | |  |  |
| .FRAS_28 (.147) 1.920 0.023 82.165 0.000 | | | |  |  |
| .FRAS_29 (.148) 1.929 0.025 77.342 0.000 | | | |  |  |
| .FRAS_30 (.149) 1.912 0.027 69.679 0.000 | | | |  |  |
| .FRAS_40 (.150) 2.079 0.030 68.923 0.000 | | | |  |  |
| .FRAS_41 (.151) 1.970 0.026 75.845 0.000 | | | |  |  |
| .FRAS_46 (.152) 1.984 0.026 75.030 0.000 | | | |  |  |
| .FRAS_48 (.153) 1.920 0.023 82.478 0.000 | | | |  |  |
| .FRAS_52 (.154) 1.850 0.024 75.833 0.000 | | | |  |  |
| .FRAS_53 (.155) 1.914 0.024 79.210 0.000 | | | |  |  |
| .FRAS_54 (.156) 1.946 0.024 81.261 0.000 | | | |  |  |
| .FRAS_11 (.157) 1.952 0.027 71.115 0.000 | | | |  |  |
| .FRAS_19 (.158) 1.937 0.027 71.071 0.000 | | | |  |  |
| .FRAS_31 (.159) 1.923 0.026 74.320 0.000 | | | |  |  |
| .FRAS_32 (.160) 2.021 0.027 73.855 0.000 | | | |  |  |
| .FRAS_38 (.161) 2.472 0.039 63.211 0.000 | | | |  |  |
| .FRAS_39 (.162) 2.085 0.032 64.596 0.000 | | | |  |  |
| .FRAS_43 (.163) 2.384 0.036 66.639 0.000 | | | |  |  |
| .FRAS_49 (.164) 1.938 0.024 79.674 0.000 | | | |  |  |
| .FRAS_13 (.165) 1.920 0.027 72.376 0.000 | | | |  |  |
| .FRAS_21 (.166) 1.948 0.027 72.141 0.000 | | | |  |  |
| .FRAS_22 (.167) 1.888 0.025 74.623 0.000 | | | |  |  |
| .FRAS_34 (.168) 2.711 0.035 76.813 0.000 | | | |  |  |
| .FRAS_36 (.169) 1.881 0.028 66.067 0.000 | | | |  |  |
| .FRAS_51 (.170) 2.218 0.032 70.204 0.000 | | | |  |  |
| .FRAS_2 (.171) 2.112 0.033 64.986 0.000 | | | |  |  |
| .FRAS_33 (.172) 2.921 0.031 94.139 0.000 | | | |  |  |
| .FRAS_37 (.173) 3.111 0.026 118.156 0.000 | | | |  |  |
| .FRAS_45 (.174) 2.739 0.035 77.579 0.000 | | | |  |  |
| .FRAS_47 (.175) 1.955 0.030 65.606 0.000 | | | |  |  |
| .FRAS_50 (.176) 2.773 0.033 84.632 0.000 | | | |  |  |
| .FRAS_12 (.177) 1.934 0.027 71.914 0.000 | | | |  |  |
| .FRAS_35 (.178) 1.977 0.029 68.559 0.000 | | | |  |  |
| .FRAS_42 (.179) 1.995 0.028 72.382 0.000 | | | |  |  |
| .FRAS_44 (.180) 2.214 0.034 65.301 0.000 | | | |  |  |
| .FRAS_3 (.181) 1.924 0.025 77.510 0.000 | | | |  |  |
| .FRAS_4 (.182) 1.930 0.025 76.603 0.000 | | | |  |  |
| .FRAS_5 (.183) 1.954 0.027 71.635 0.000 | | | |  |  |
| D1 0.038 0.034 1.114 0.265 | | | |  |  |
| D2 0.025 0.038 0.643 0.520 | | | |  |  |
| D3 0.011 0.041 0.272 0.786 | | | |  |  |
| D4 0.030 0.032 0.947 0.344 | | | |  |  |
| D5 0.009 0.048 0.193 0.847 | | | |  |  |
| D6 0.053 0.042 1.270 0.204 | | | |  |  |
|  |  |  |  |  |  |
| Variances: |  |  |  |  |  |
| Estimate Std.Err z-value P(>\|z\|) | | | |  |  |
| .FRAS_1 (.70.) 0.155 0.013 11.826 0.000 | | | |  |  |
| .FRAS_6 (.71.) 0.078 0.007 11.678 0.000 | | | |  |  |
| .FRAS_7 (.72.) 0.075 0.006 11.632 0.000 | | | |  |  |
| .FRAS_8 (.73.) 0.138 0.012 11.774 0.000 | | | |  |  |
| .FRAS_9 (.74.) 0.275 0.023 11.872 0.000 | | | |  |  |
| .FRAS_10 (.75.) 0.439 0.037 11.911 0.000 | | | |  |  |
| .FRAS_14 (.76.) 0.046 0.004 11.526 0.000 | | | |  |  |
| .FRAS_15 (.77.) 0.080 0.007 11.641 0.000 | | | |  |  |
| .FRAS_16 (.78.) 0.040 0.004 11.352 0.000 | | | |  |  |
| .FRAS_17 (.79.) 0.073 0.006 11.634 0.000 | | | |  |  |
| .FRAS_18 (.80.) 0.066 0.006 11.581 0.000 | | | |  |  |
| .FRAS_20 (.81.) 0.047 0.004 11.414 0.000 | | | |  |  |
| .FRAS_23 (.82.) 0.317 0.027 11.914 0.000 | | | |  |  |
| .FRAS_24 (.83.) 0.066 0.006 11.674 0.000 | | | |  |  |
| .FRAS_25 (.84.) 0.049 0.004 11.545 0.000 | | | |  |  |
| .FRAS_26 (.85.) 0.052 0.005 11.528 0.000 | | | |  |  |
| .FRAS_27 (.86.) 0.226 0.019 11.916 0.000 | | | |  |  |
| .FRAS_28 (.87.) 0.060 0.005 11.663 0.000 | | | |  |  |
| .FRAS_29 (.88.) 0.046 0.004 11.465 0.000 | | | |  |  |
| .FRAS_30 (.89.) 0.106 0.009 11.755 0.000 | | | |  |  |
| .FRAS_40 (.90.) 0.175 0.015 11.841 0.000 | | | |  |  |
| .FRAS_41 (.91.) 0.108 0.009 11.795 0.000 | | | |  |  |
| .FRAS_46 (.92.) 0.117 0.010 11.806 0.000 | | | |  |  |
| .FRAS_48 (.93.) 0.063 0.005 11.686 0.000 | | | |  |  |
| .FRAS_52 (.94.) 0.072 0.006 11.701 0.000 | | | |  |  |
| .FRAS_53 (.95.) 0.079 0.007 11.744 0.000 | | | |  |  |
| .FRAS_54 (.96.) 0.088 0.007 11.783 0.000 | | | |  |  |
| .FRAS_11 (.97.) 0.111 0.009 11.862 0.000 | | | |  |  |
| .FRAS_19 (.98.) 0.069 0.006 11.400 0.000 | | | |  |  |
| .FRAS_31 (.99.) 0.063 0.006 11.438 0.000 | | | |  |  |
| .FRAS_32 (.100) 0.173 0.015 11.934 0.000 | | | |  |  |
| .FRAS_38 (.101) 0.406 0.034 11.924 0.000 | | | |  |  |
| .FRAS_39 (.102) 0.260 0.022 11.929 0.000 | | | |  |  |
| .FRAS_43 (.103) 0.363 0.030 11.916 0.000 | | | |  |  |
| .FRAS_49 (.104) 0.072 0.006 11.743 0.000 | | | |  |  |
| .FRAS_13 (.105) 0.070 0.006 11.609 0.000 | | | |  |  |
| .FRAS_21 (.106) 0.066 0.006 11.480 0.000 | | | |  |  |
| .FRAS_22 (.107) 0.056 0.005 11.427 0.000 | | | |  |  |
| .FRAS_34 (.108) 0.352 0.030 11.917 0.000 | | | |  |  |
| .FRAS_36 (.109) 0.123 0.010 11.927 0.000 | | | |  |  |
| .FRAS_51 (.110) 0.283 0.024 11.917 0.000 | | | |  |  |
| .FRAS_2 (.111) 0.217 0.018 11.809 0.000 | | | |  |  |
| .FRAS_33 (.112) 0.223 0.019 11.870 0.000 | | | |  |  |
| .FRAS_37 (.113) 0.072 0.007 10.487 0.000 | | | |  |  |
| .FRAS_45 (.114) 0.343 0.029 11.913 0.000 | | | |  |  |
| .FRAS_47 (.115) 0.091 0.009 10.423 0.000 | | | |  |  |
| .FRAS_50 (.116) 0.301 0.025 11.915 0.000 | | | |  |  |
| .FRAS_12 (.117) 0.056 0.005 10.535 0.000 | | | |  |  |
| .FRAS_35 (.118) 0.091 0.008 11.435 0.000 | | | |  |  |
| .FRAS_42 (.119) 0.102 0.009 11.760 0.000 | | | |  |  |
| .FRAS_44 (.120) 0.296 0.025 11.944 0.000 | | | |  |  |
| .FRAS_3 (.121) 0.050 0.005 9.705 0.000 | | | |  |  |
| .FRAS_4 (.122) 0.041 0.005 8.801 0.000 | | | |  |  |
| .FRAS_5 (.123) 0.058 0.006 9.557 0.000 | | | |  |  |
| D1 0.055 0.014 3.886 0.000 | | | |  |  |
| D2 0.056 0.014 3.873 0.000 | | | |  |  |
| D3 0.060 0.015 4.136 0.000 | | | |  |  |
| D4 0.025 0.010 2.554 0.011 | | | |  |  |
| D5 0.085 0.020 4.236 0.000 | | | |  |  |
| D6 0.070 0.016 4.479 0.000 |  |  |  |  |  |
