# Supplementary material for: Factor structure and reliability of the Family Resilience Scale (FRAS): adaptation with Colombian families exposed to stressful events
Source: Front Psychol. 2025 Sep 24;16:1568139. doi: 10.3389/fpsyg.2025.1568139 (PMC12506929; doi:10.3389/fpsyg.2025.1568139)
Supplement: Supplementary file 4 [file Table_1.docx]

**Factor Structure and Reliability of the Family Resilience Scale (FRAS): Adaptation with Colombian Families Exposed to Stressful Events**

The exploratory factor analysis (EFA) using the oblimin rotation identified three factors explaining 59% of the cumulative variance. Most items showed strong loadings (≥0.40) on their respective factors, with minimal cross-loadings, although FRAS_37 exhibited a negative loading. Factor correlations were low to moderate, indicating related but distinct dimensions. The results support a three-factor solution for the dataset.

Table 1. Exploratory factor analysis (EFA)

| **Item** | **Dimension 1** | **Dimension 2** | **Dimension 3** |
| --- | --- | --- | --- |
| FRAS_1 | 0,70 |  |  |
| FRAS_3 | 0,73 |  |  |
| FRAS_4 | 0,79 |  |  |
| FRAS_5 | 0,81 |  |  |
| FRAS_6 | 0,77 |  |  |
| FRAS_7 | 0,83 |  |  |
| FRAS_9 |  |  | 0,69 |
| FRAS_10 |  |  | 0,72 |
| FRAS_12 | 0,85 |  |  |
| FRAS_13 | 0,84 |  |  |
| FRAS_14 | 0,84 |  |  |
| FRAS_15 | 0,90 |  |  |
| FRAS_16 | 0,88 |  |  |
| FRAS_19 | 0,85 |  |  |
| FRAS_20 | 0,85 |  |  |
| FRAS_21 | 0,86 |  |  |
| FRAS_22 | 0,82 |  |  |
| FRAS_23 |  | 0,52 |  |
| FRAS_24 | 0,83 |  |  |
| FRAS_25 | 0,83 |  |  |
| FRAS_26 | 0,86 |  |  |
| FRAS_28 | 0,81 |  |  |
| FRAS_29 | 0,87 |  |  |
| FRAS_30 | 0,75 |  |  |
| FRAS_31 | 0,75 |  |  |
| FRAS_34 |  | 0,68 |  |
| FRAS_35 | 0,84 |  |  |
| FRAS_37 | -0,80 |  |  |
| FRAS_38 |  |  |  |
| FRAS_42 | 0,78 |  |  |
| FRAS_43 |  | 0,43 |  |
| FRAS_46 | 0,61 |  |  |
| FRAS_47 | 0,72 |  |  |
| FRAS_48 | 0,69 |  |  |
| FRAS_49 | 0,72 |  |  |
| SS loadings | 18,96 | 1,85 | 1,67 |
| Proportion Var | 0,50 | 0,05 | 0,04 |
| Cumulative Var | 0,50 | 0,55 | 0,59 |
| Proportion Explained | 0,84 | 0,08 | 0,07 |
| Cumulative Proportion | 0,84 | 0,93 | 1,00 |

KMO: 0.94 – Bartlett: p value 0.000
